# Is social connectedness still in decline after the Covid-19 pandemic? Cohort trends in secondary school students in Finland between 2017 and 2023

**DOI:** 10.1186/s40359-025-03394-5

**Published:** 2025-09-29

**Authors:** Sanna Read, Noona Kiuru, Jenni Helenius, Niina Junttila, Katariina Salmela-Aro

**Affiliations:** 1 Care Policy and Evaluation Centre, London School of Economics and Political Science, London, UK; 2 Faculty of Educational Sciences, University of Helsinki, Helsinki, Finland; 3 Departent of Psychology, University of Jyväskylä, Jyväskylä, Finland; 4 Finnish Institute for Health and Welfare, Helsinki, Finland; 5 Faculty of Educational Sciences, University of Turku, Turku, Finland

**Keywords:** Loneliness, Close friends, Belonging at school, Trends, Sociodemographic factors, Immigration, secondary school students

## Abstract

Several previous cohort studies suggest declining social connectedness in young people since 2000, the pattern accelerated during the covid-19 pandemic. Little is known whether social connectedness had recovered to the pre-pandemic levels. We investigated whether the declining pattern in experienced social connectedness in secondary school students between 2017 and 2021 changed in 2023. We also investigated the role of individual and school characteristics in these trends. We used nationally representative Finnish data of students in lower and upper secondary education (analytic *n* = 556,424–557,391). Social connectedness was measured by number of close friends, feelings of loneliness and sense of belonging at school. Regression analyses included the fixed effects and the interactions of year, gender, school level, parental education, immigration status of the student and urban-rural location. The results showed that the overall declining trend of social connectedness between 2017 and 2021 slowed down between 2021 and 2023: number of close friends further declined at a slower rate (1% decline), while there was no change in sense of belonging at school and a slight recovery (2% reduction) in feelings of loneliness. In some subgroups, such as students in general upper secondary schools and students with immigration background had a faster recovery than other student groups. The results point to little overall improvement in social connectedness in secondary school students in Finland after the pandemic.

**Supplementary Information:**

The online version contains supplementary material available at 10.1186/s40359-025-03394-5.

## Introduction

Several previous studies have revealed declining social connectedness in school students in the recent years [1–3]. It appears that the declining trend had already started before the covid-19 pandemic, but further accelerated during it. Considering that close and supportive social relationships are pivotal for young people’s development and wellbeing, it is important to study whether there has been any change in the trends after the pandemic and understand the role of underlying background characteristics, such as sociodemographic factors, in these trends.

It is well-known that close social relationships are important to individuals’ adaptive functioning and wellbeing as they meet human’s fundamental need of relatedness and belonging [4, 5]. Interpersonal relationships are especially important during the years of formal education when young people search their place in their social groups, family, school, work and wider society. They lay basis for learning, coping with stress and wellbeing [5, 6], whereas a persistent lack of meaningful social relationships may lead to mental health problems and feelings of loneliness and isolation [7]. In the present study, we examine three key indicators - number of close friends, feelings of loneliness and sense of belonging at school - to map the trends in young people’s social connectedness.

### Social connectedness in young people

Social connectedness refers to the feelings of closeness to others and a sense of belonging to a group, such as family, school and community [8, 9]. Over time, social connectedness can generate social capital which refers to resources derived from social relationships. Social capital contributes to individuals’ health and wellbeing. Social connectedness is a broad concept which can be measured in many ways. It can include components of relationship, feelings of bond or connection to others and feelings of being valued within a relationship [9], referring to meaningful relationships and not feeling lonely [4]. Social connectedness may be also defined as feelings of being accepted, respected and included in the groups, for instance school class [10]. There are also other aspects of social connectedness, such as diversity and frequency on contacts or perceived satisfaction, support or strain related to the social relationships. Despite the variations in measurements, the overarching theme is to include components of connection or bond to others and feelings that the person’s individuality is validated by these social connections [9].

In the context of young people, close relationships with friends [11] and feelings of loneliness are important [12–14], as well as the sense of belonging in the school community [15–17]. These different dimensions of social connectedness are often interconnected [14, 17], for instance those with close friendships may be less likely to feel lonely. Sense of belonging can be also defined from a wider perspective of ‘multiple constellations across social, national, and cultural borders’ [18] or as a feeling of being ‘at-home’ [19] while contrasted against feelings of ‘non-belonging’ [20]. These definitions point to the importance of investigating the role of contextual factors, such as family socioeconomic status, immigrations status and urban-rural location, in nationally representative consecutive cohorts of young people.

### Cohort trends in social connectedness

Several previous studies, many of them carried out in the Nordic context, have pointed to the declining trends of social connectedness in school students in the recent years. For instance, perceived global loneliness increased in a cross-sectional cohort sample of about 20,000 11-15-year-olds in Finland between 2006 and 2018 [21]. In a large multinational cross-sectional dataset (PISA) of about one million 15-16-year-olds, school loneliness was measured with six items since 2000 and showed an overall increase between 2012 and 2018 [1], also reported between 2015 and 2018 by OECD [22]. Based on the same international comparison [22], there was an overall decline in all countries in school belongingness (a sum of six items) between 2015 and 2018. Using the same outcome and a subset of the PISA cohort of about 18,000 students, a Swedish study found declining trends of school belonging between 2000 and 2018 among 15–16-year-old students [2].

A declining trend in social connectedness have been reported during the covid-19 pandemic, i.e. increased social isolation and loneliness and decreased sense of connectedness [23–25]. These studies included various age groups and sample sizes of children and young people and used a range of methods from qualitative to quantitative. No changes in the feelings of social isolation or quality of friendship during the pandemic have also been reported [26], based on a longitudinal analysis of nearly 800 10-17-year-olds in Western Australia.

An accelerated decline in social connectedness after 2019 has been found in studies that cover time before and during the covid-19 pandemic. In a study of nearly 220,000 respondents from the United States, social engagement and companionship, measured as minutes spent with friends and other people or anyone, declined most in the youngest group (15-24-years) relative to all other ages between 2002 and 2020 [27]. This change was especially fast between 2018 and 2020. This study also showed that the decline in social engagement with friends and other people in the youngest age group was not replaced by more social engagement with family. Evidence from five European countries show that the youngest age groups of adults (age 18–39) were the loneliest - a trend that intensified during the pandemic between April 2020 and November 2022 [28]. The same study highlighted the adverse impact of the pandemic policies on loneliness: the rates of loneliness rising with containment policies and falling with relaxation of the social restrictions and increased government economic support. Meanwhile, the intensity of the pandemic (measured as number of recent covid-19 deaths) had only a minor impact.

A study in Finland used a cross-sectional sample of about 450,000 students in lower, general upper and vocational secondary schools. This study showed an increase in loneliness, and a decline in having three or more close friends and in sense of belonging at school between 2017 and 2021 [3]. The number of close friends declined, and feelings of loneliness increased most between 2019 and 2021, suggesting a potential impact of the pandemic on accelerated decline in social connectedness. Another study using partly the same data reported increased loneliness in Finnish secondary schools between 2019 and 2021 [29]. In belonging at school, the decline was sharp already between 2017 and 2019 and continued further to 2021 [3]. Another study using about 150,000 Danish students aged 11–15 revealed a similar trend of school connectedness (a 7-item scale) already declining before the pandemic (2015–2019) when compared to the consecutive cohorts of students during the pandemic (2017–2021 and 2019–2023) [30]. The patterns of trends in this study suggested little pandemic-specific cohort impacts on school connectedness. Apart from the social restrictions related to the pandemic, other possible reasons for the above-mentioned trends of increasing loneliness and declining sense of belonging may be increasing use of social media and smartphones, general declining wellbeing and economic trends [1, 31] and changes in national school policies [2, 3].

### Inequalities in social connectedness

It is well-known that social capital itself can be a factor reproducing inequality [32, 33]. Families with more resources such as a higher parental education level may initially provide more beneficial social networks for young people to build on [34, 35]. On the other hand, people with lower socioeconomic background and those migrated to a new environment may find it harder to make the necessary social ties to support their needs [35–37]. Lower socioeconomic status or being a migrant may however in some cases be associated with characteristics that are beneficial for social connectedness, e.g. prosocial behaviour [35, 38] and stronger familial and community support networks [39–41]. Far less is known about the cohort trends in social connectedness in urban and rural environments. Urban environment with larger population may provide more chances of forming social relationships compared to rural locations with fewer people [42]. However, urban environments may lack the opportunities for close and deeper social interactions and feelings of belonging to a community which may be easier in smaller settings where people know each other better [43].

Previous studies suggest that the trends of social connectedness differ between the students, depending on their background characteristics. It was generally assumed that there would be growing inequalities related to for instance low income, non-white ethnicity and urban residency during the pandemic [44–47]. These inequalities were evident – already before the pandemic - in mental health, learning and wellbeing of the school students but were also expected to erode social capital, while affecting the equal rights for social participation and inclusion [45, 47]. Few studies have been published on the possible moderating background characteristics. In a review on children and adolescents’ social connectedness and wellbeing during the pandemic, the authors concluded that many background characteristics such as immigration status and geographic regions were not systematically reported [48].

These above-mentioned findings point to the under-examined role of social determinants, such as socioeconomic factors and ethnic background. Although social capital itself can be a factor reproducing inequality [32, 33], these pathways may be different for young people who are at the early stages of forming their social networks. Moreover, the expected outcomes of the role of inequalities may not follow the same route in exceptional circumstances, such as nationally or internationally imposed social restrictions and school closures, and they may be shaped by other individual and family characteristics [40]. Hence, the findings of the role of inequalities in social connectedness or wellbeing may not be consistent.

For instance, there are many studies showing that lower socioeconomic status, such as lower educational, occupational or income level in the family, is associated with less social connectedness in adolescents [22, 49, 50]. However, when looking at the cohort trends over time, the picture is more complex: While school belongingness declined most in foreign-born students, students from disadvantaged background and low achieving students in Sweden between 2000 and 2018, initially in 2000 there were little differences in school belongingness by these background characteristics [2]. The changes in background demographical factors or school environment during this period were not associated with the decline.

In Danish adolescents, social inequality decreased in perceived global loneliness in the successive cohorts of nearly 20,000 11–15-year-olds from 1991 to 2014 [36]. The results in Finnish secondary schools showed a similar pattern: the social gradient related to parental higher education, urban-rural location and immigration status of the student appeared to diminish over time between the advantaged and disadvantaged groups of students [3]. For instance, between 2019 and 2021 (co-occuring with the pandemic) social connectedness in those with immigration background declined less than those with no immigration background, resulting in higher levels of school belonging in 2021 in some groups of students with immigration background compared to those with no immigration background. Overall, the results suggested that while some socio-demographically disadvantaged groups, such as those having lower parental education and immigrants, showed lower levels of social connectedness, there were differences by gender, school level and year. Many of these differences diminished because the more advantaged groups declined faster, i.e. moved towards the less advantaged groups.

### The role of gender and school level in social connectedness

The previous work shows the important role of gender and school level along with socioeconomic background in shaping the trends of social connectedness [3]. In some studies, social connectedness has been found to be higher in girls compared to boys [51], while others show no gender differences when the geographic sampling was taken into account and when looking at the more recent studies [52].

Several recent studies report higher social connectedness in boys compared to girls, suggesting a widening gender gap over the last 20 years. For instance, boys aged 15–16 reported higher school belonging compared to their female counterparts in Finland in 2015 [53]. When following up the cohorts of 15-16-year-olds over time in a multinational study, girls reported more loneliness between 2015 and 2018 compared to boys [1]. In a Swedish study, school belonging was higher in boys compared to girls between 2000 and 2018 with a sharper decline in girls compared to boys between 2015 and 2018 [2]. Another study in Norway, focusing only on upper secondary school students showed higher levels of loneliness in girls, but a faster increase in loneliness in boys between 2014 and 2018 [54]. Sense of social isolation was consistently higher in girls compared to boys in a follow-up between 2018 and 2021 (before and during pandemic) in Western Australia [26].

In secondary school (lower, general upper and vocational) in Finland between 2017 and 2021, girls generally reported having fewer close friends, more loneliness and lower school belonging compared to boys [3]. Students in lower secondary school reported having fewer close friends than students in general upper and vocational schools. The decline in social connectedness during the pandemic was especially large in girls and general upper secondary school: the number of close friends declined faster in girls compared to boys, while loneliness increased faster in students in general upper secondary school compared to lower secondary and vocational schools. These groups, especially girls, might have been most affected by the pandemic as they tend to have fewer social connections to start with. Moreover, these groups often have higher academic aspirations but simultaneously experience school-related stress [55], which especially in the pandemic-induced upheaval in learning and social support may impact social relationships [3]. The results above point to the importance of taking into account gender and school level and their interactions in the models of social connectedness.

### The current study

The research mentioned above highlights the declining trends of social connectedness in the recent years. Little is however known if the declining trend continued, had stalled or even improved after the covid-19 pandemic. Only few studies have followed up the trends of social connectedness for a longer period including time before and during the pandemic and used nationally representative samples of students [3, 27, 29]. None of these covered the years after 2021. The role of background characteristics of the students and school in these trends is little known [48]. The current study investigated the following research questions:

1. Does the declining trend of social connectedness (having fewer close friends, increased feelings of loneliness and lower sense of belonging at school) in secondary school students in Finland between 2017 and 2021 continue, stop or reverse to better between 2021 and 2023 as the pandemic period with social restrictions is over? Based on the previous trends found in Finland between 2017 and 2021 suggesting an accelerated decline in close friends and increase in loneliness during the pandemic [3], these measures of social connectedness may show slowdown or even recovery between 2021 and 2023 if the accelerated decline between 2019 and 2021 was related to pandemic. There may be differences in the rates of change between the dimensions of social connectedness: lifting the social restrictions may have an immediate impact on feelings of loneliness [28], whereas number of close friends may take a longer time to recover. In sense of belonging at school, there was a faster decline between 2017 and 2019 and a further smaller decline between 2019 and 2021, suggesting that there may be also other societal factors than pandemic-related factors at play. A similar finding has also been reported in student cohorts in Denmark [30]. We therefore expect that that the declining trend in school belonging may continue between 2021 and 2023, although not as strong if it follows the slower rate of decline found between 2019 and 2021.
2. Does any subgroup of students by gender, school level, higher parental education, immigration status of the student or urban-rural location show recovery in social connectedness by 2023, and are there differences in recovery between the subgroups (e.g. poorer recovery related to female gender, lower parental education, being an immigrant)? Pandemic had been expected to hit hardest of those with the least social and socioeconomic resources (girls, lower educated, immigrants, densely populated areas) or those who experienced more social restrictions in school (older students at higher school levels). Consequently, their return back to normal would be also challenging [44–47] which would suggest poorer recovery for the disadvantaged groups. The previous cohort trend studies in Finland and Denmark show declining social gradient, i.e. differences between the subgroups getting smaller, in social connectedness before and during the pandemic [3, 36]. We expect the diminishing differences by subgroups to continue.

## Materials and methods

### Sample and data collection

We used the data from the Finnish School Health Promotion Study (SHP). The SHP is a bi-annual anonymous classroom census survey on health and wellbeing of Finnish 14 − 20-year-old adolescents [56, 57]. The national survey targets the school year cohorts in the participating age groups. The survey was anonymous, confidential, and participation was voluntary. The students filled in the online questionnaire in a classroom setting. They gave informed consent by answering the survey and had the opportunity to decline to respond. Parents were informed about the study in advance and had the right to withdraw their child under the age of 15 from participating in the survey. Those students who were not at school on the day the survey was conducted did not respond to the survey. The survey follows the principle of passive consent, based on the rationale that it is a broad population-level survey. In this case it is sufficient to inform the parent or carer of a child under the age of 15 about the research so that they can refuse their child’s participation in the research if they wish. The number of respondents is large if the survey targets at least 400 people. Informing the parent or carer is also sufficient in research that does not involve the processing of the personal data of the minor participant. This procedure following the Finnish National Board of Research Integrity (TENK) ethical principles [58] was included in the research plan approved by the Institutional review board (IRB) of the Finnish Institute for Health and Welfare (THL). A description of the survey has been reported elsewhere [56, 57]. The details about the data collection can be found in SHP Scientific Research Privacy notices [59]. The present study was conducted in compliance with the 1964 Helsinki declaration.

The current study used data from four time points: 2017, 2019, 2021 and 2023. At each time point the collection period was between the 1st March and 12th May. The school levels included lower secondary school (basic education), general upper secondary schools with a matriculation exam at the end (here refereed as “upper secondary school” to keep short) and vocational secondary schools which provide an alternative of vocational track to general upper secondary school with a vocational qualification at the end (here refereed as “vocational school” to keep short) [60]. The data included 339,600 students in 832 lower secondary, 171,390 students in 367 upper secondary and 91,098 students in 120 vocational schools. See the *n* for each variable in Tables 1 and 2. The analytic sample sizes in the models were *n* = 556,754 for having close friends, *n* = 557,391 for loneliness and *n* = 556,424 for belonging at school. The participation rates in 2017, 2019, 2021 and 2023 were the following: 63%, 73%, 75% and 70% for lower secondary, 54%, 69%, 71% and 68% for upper secondary and about 30% in all years for vocational schools. The students in lower secondary school were 14–16 years (class 8 and 9), and in upper secondary and vocational school 16–20 years (1st and 2nd year students).

**Table 1:** Distributions of individual and school characteristics (%) in the school health promotion study 2017–2023 in girls and boys in lower and upper secondary and vocational school

	Girls - Lower	Girls - Upper	Girls - Vocational	Boys - Lower	Boys - Upper	Boys - Vocational
Year (%)	*n* = 171,651	*n* = 100,689	*n* = 37,273	*n* = 166,709	*n* = 70,286	*n* = 53,484
2017	21.5	19.9	28.6	21.8	20.4	29.5
2019	25.7	26.1	25.1	25.8	26.0	26.2
2021	27.2	28.0	24.5	26.8	27.2	23.7
2023	25.7	26.0	21.8	25.7	26.4	20.6
Parent(s) with higher education degree (%)	*n* = 162,170 53.9	*n* = 99,147 66.4	*n* = 35,591 33.6	*n* = 149,708 53.9	*n* = 68,085 72.3	*n* = 48,932 38.1
Immigration status (%)	*n* = 167,232	*n* = 99,831	*n* = 36,396	*n* = 154,145	*n* = 68,652	*n* = 50,128
Student and parents born in Finland	86.4	87.7	88.3	85.9	87.5	88.6
One parent foreign-born^a^	8.0	7.4	6.7	7.2	7.2	5.9
Born in Finland with foreign-born parents	2.4	2.2	1.6	2.2	2.2	1.6
Born abroad with foreign-born parents	3.3	2.8	3.3	4.7	3.1	3.9
Urban-rural area (%)	*n* = 171,650	*n* = 100,689	*n* = 37,273	*n* = 166,709	*n* = 70,286	*n* = 53,484
Urban	67.9	75.3	79.5	67.5	74.9	75.7
Semi-urban	18.1	14.4	14.7	18.1	15.1	19.2
Rural	14.0	10.3	5.8	14.4	10.1	5.1

^a^ In addition to the students with one parent foreign-born, this category also includes students born abroad and a parent born in Finland. The proportions of these two groups within this category are 76% and 24%, respectively

**Table 2:** Distributions of the social connectedness variables shown as % or mean and standard deviation (SD) in the school health promotion study 2017–2021 in girls and boys in lower and upper secondary and vocational school

Variable	Girls - Lower	Girls - Upper	Girls - Vocational	Boys - Lower	Boys - Upper	Boys - Vocational
Close friends (%)	*n* = 170,234	*n* = 100,335	*n* = 36,954	*n* = 162,582	*n* = 69,590	*n* = 52,470
None	7.6	6.7	6.7	11.3	10.2	9.1
One	21.6	19.3	22.6	16.4	17.0	16.2
Two	24.3	23.4	26.3	16.2	17.2	15.4
Three or more	46.5	50.7	44.4	56.1	55.7	59.3
Loneliness (%)	*n* = 170,416	*n* = 100,397	*n* = 36,996	*n* = 162,966	*n* = 69,697	*n* = 52,539
Never	16.1	11.9	14.3	40.6	28.7	41.4
Very seldom	32.3	33.2	29.3	34.3	38.6	32.4
Sometimes	33.6	36.8	35.8	17.4	23.0	18.5
Quite often	13.2	13.9	15.2	4.8	6.4	5.2
All the time	4.8	4.2	5.5	2.9	2.2	2.5
Belonging at school (mean, SD)	*n* = 169,641	*n* = 100,271	*n* = 36,701	*n* = 162,109	*n* = 69,636	*n* = 51,944
	3.1 (1.0)	3.3 (1.0)	3.4 (1.0)	3.5 (1.0)	3.6 (1.0)	3.7 (0.9)

### Measures

#### Social connectedness

Social connectedness can be conceptualized and measured in various ways. In the present study, a combination of indicators — number of close friends, loneliness, and school belonging— was based on prior research using the same variables, which enables comparability over time and supports the monitoring of trends in social connectedness across studies, see [3]. Items were selected based on the previous experiences so that they were suitable for large-scale population data collection. They were tested in focus groups before administering the survey.

*Number of close friends* was asked with a single item: “At the moment, do you have a close friend with whom you can talk confidentially about almost everything concerning yourself?”. It had four categories: 0 = no close friends, 1 = one close friend, 2 = two close friends, 3 = several close friends (three or more). The indicator measuring the number of close friends was included in the survey since its inception in 1996 and has remained unchanged to ensure the continuity of long-term trend monitoring.

In 2017, a measure for *feelings of loneliness* was added, inspired by the short UCLA Loneliness Scale to measure social and emotional loneliness in a large-scale population survey [61, 62]. A single-item question (“Do you ever feel lonely?“) was measured with five categories: 1 = never, 2 = very rarely, 3 = sometimes, 4 = fairly often, 5 = all the time (for validity information, see also [21, 63, 64].

Belonging at school, also introduced in 2017, was a mean of two items: “I feel I am an important member of my: 1) classroom community, 2) school community”. The statements were rated on a 5-point scale from 1 = fully disagree to 5 = fully agree. The correlation between the school belonging items was 0.84 in 2017, 0.75 in 2019 and 2021, and 0.76 in 2023. The measure is conceptually grounded in established models of school connectedness, with items similar to what have been used in the international PISA data collections [65], but its validity may vary depending on school context and student interpretation.

#### Individual and school characteristics

Gender was a dichotomous indicator for (0) boy and (1) girl. The level of school had three categories: (1) lower secondary, (2) upper secondary and (3) vocational school. A dichotomous measure was used for the educational level of the parents: (0) below degree in higher education, (1) at least one parent had a degree in higher education. Immigration status was based on the student-reported country in which they and their parents were born [66]. Four categories were created: (1) student and parents born in Finland, i.e. no immigration background, (2) one parent foreign-born, or the student born abroad and a parent born in Finland, i.e. partial immigration background (3) foreign-born parents, student born in Finland, i.e. second-generation immigrant, (4) foreign-born parents, student born abroad, i.e. first-generation immigrant. Urban-rural characteristics of the area the school was located were measured using three categories: (1) urban, (2) semi-urban, (3) rural [66].

### Analysis

First, we illustrated the general trends of the three social connectedness outcomes (number of close friends, loneliness, belonging at school) between 2017 and 2023. Second, we carried out regression analyses to assess the effect of time so that we included an interaction term which allowed the variation between the study year, school level and gender (Model 1 in the regressions). Gender and school level have been found to shape the trend in the social connectedness items and further modify the associations of other characteristics with social connectedness trends [3]. Consequently, as a third step, we investigated the variation in these trends by other individual and school characteristics (parental education, immigrations status of the student and urban-rural location) (Model 2 in the regressions). This was done by adding these variables in the interaction term with the study year, school level and gender and dropping the higher-order interactions if they were not significant. We focused especially on the changes in trend between the two latter time points, 2021 and 2023, to investigate if there was any change compared to the trend between 2019 and 2021.

We used Poisson regression for the number of close friends as this variable is a count with about half of the students reporting three or more close friends (the highest category). Loneliness was slightly skewed with fewer students reporting being quite often or all the time lonely (see Table 2). For this reason, we carried out the regressions for loneliness using Generalised Linear Models (GLM) with log link and gamma distribution. Belonging at school was approximately normal, and therefore we carried out regressions for this variable using GLM with identity link and gaussian distribution.

To determine whether an interaction term was necessary to keep in the model, the Wald test for the interaction term was carried out. A *p*-value smaller than 0.05 was used as an indication of a significant interaction effect. The model estimates and Wald tests for the interactions in each step and the unstandardized estimates, predictive margins for the interactions and estimation for the final models are shown in Supplementary material 1. Using the margins from the final models (Supplementary material 1), the change in percentage was calculated between 2019 and 2021 and compared to change between 2021 and 2023 and the overall change between 2019 and 2023 (Supplementary material 2). The description of correlations between the variables and missingness are shown in Supplementary material 3.

The three measures of social connectedness were moderately correlated: the polychoric correlations for ordered categorical variables were 0.33 between sense of belonging at school and having close friends, -0.43 between sense of belonging at school and loneliness, and − 0.43 between having close friends and loneliness. The measures were used separately as an outcome in each model as they were only partially overlapping. Separate modelling allowed the results and interpretation to vary by outcome. The associations between the background factors (year, gender, school level, parental education, immigration status of the student and urban-rural location) were small and did not show collinearity (Supplementary material 3).

Missingness was low: 0.3% of the values were missing for the gender of the student, 6% for parental education, 4% for immigration status, 2% for belonging at school and 1% for having close friends and loneliness. There was no missingness for school level and urban-rural classification. In sense of belonging at school, missingness was 5.7% in 2017 compared to 0.7%, 0.7% and 0.4% in 2019, 2021 and 2023, respectively. In other measures, the differences in the proportion of missingness between the categories of the background factors were 2% points or less (Supplementary material 3). Missingness was not completely at random (not MCAR) (Supplementary material 3). Because missingness was low and the sample size large, complete cases were used in the models.

## Results

### Descriptive results

Table 1 shows the distributions of individual and school characteristics in girls and boys in lower and upper secondary and vocational school. More than half of the students in lower secondary school had at least one parent with higher education degree. In upper secondary school the proportion was even higher, 66% in girls and 72% in boys. In vocational schools about a third of the students had a parent with higher education degree. About 6–8% of the students had a foreign-born parent or were born abroad with one parent born in Finland. A smaller proportion were born in Finland (2%) to foreign-born parents or both the student and parents were born abroad (3–5%). About 68% of the students in lower secondary school, 75% in upper secondary school and 78% in vocational schools were in an urban area.

Table 2 shows the distributions of social connectedness in girls and boys in lower and upper secondary and vocational school. Between 44 and 51% of girls and 56–59% of boys reported having three or more close friends, while 7–8% of girls and 9–11% of boys had no close friends. About a third of girls and less than a quarter of boys felt sometimes lonely. Those quite often or all the time lonely was higher in girls (about 18–20%) compared to boys (about 8–10%). Sense of belonging at school was somewhat greater in boys and in vocational schools.

### General trends in social connectedness since 2017 when year 2023 was added

**Fig. 1:**
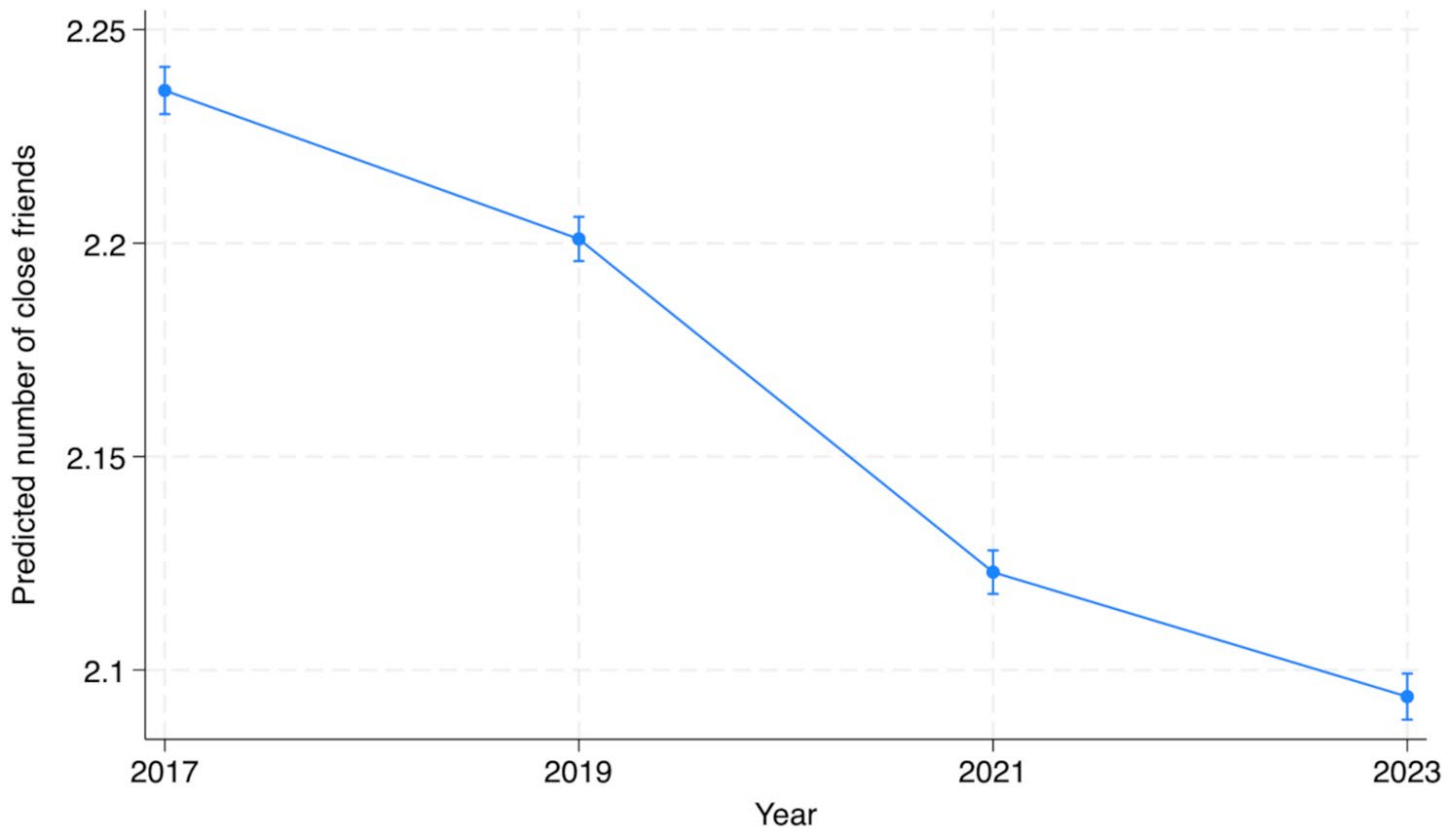
Number of close friends in secondary (lower, upper and vocational) school students in Finland between 2017 and 2023. The estimated mean is shown from a model adjusted for school level, gender, parental education, immigration status of the student and urban/rural location. Spikes indicate the 95% confidence interval

The overall declining trend of number of close friends between 2017 and 2021 slowed down between 2021 and 2023. Number of close friends declined by 4% between 2019 and 2021, followed by a small decline of 1% between 2021 and 2023. The estimated means were 2.24 in 2017, 2.20 in 2019, 2.12 in 2021 and 2.09 in 2023 (Fig. 1; see the estimated margins and confidence intervals (CIs) in Supplementary material 1). Having more close friends was reported by boys, student in vocational and upper secondary schools (compared to lower secondary school), schools in more urban locations, students with higher parental education degree and no immigration background (Supplementary material 1).

**Fig. 2:**
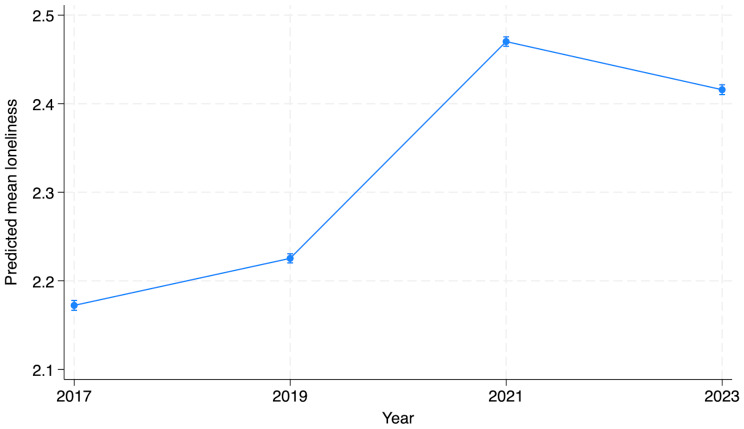
Feelings of loneliness in secondary (lower, upper and vocational) school students in Finland between 2017 and 2023. The estimated mean is shown from a model adjusted for school level, gender, parental education, immigration status of the student and urban/rural location. Spikes indicate the 95% confidence interval

The overall increasing trend of loneliness between 2017 and 2021 slightly recovered between 2021 and 2023. Feelings of loneliness increased by 11% between 2019 and 2021 and was followed by a slight decline between 2021 and 2023 amounting to a 2% decrease overall. The estimated means were 2.17 in 2017, 2.22 in 2019, 2.47 in 2021 and 2.42 in 2023 (Fig. 2 and Supplementary material 1). Feelings of loneliness were reported more often by girls, students in vocational and upper secondary schools (compared to lower secondary school), schools in more urban locations, students with higher parental education, those with no immigration background, one foreign-born parent or student and parents born abroad compared to students born in Finland to foreign-born parents (Supplementary material 1).

**Fig. 3:**
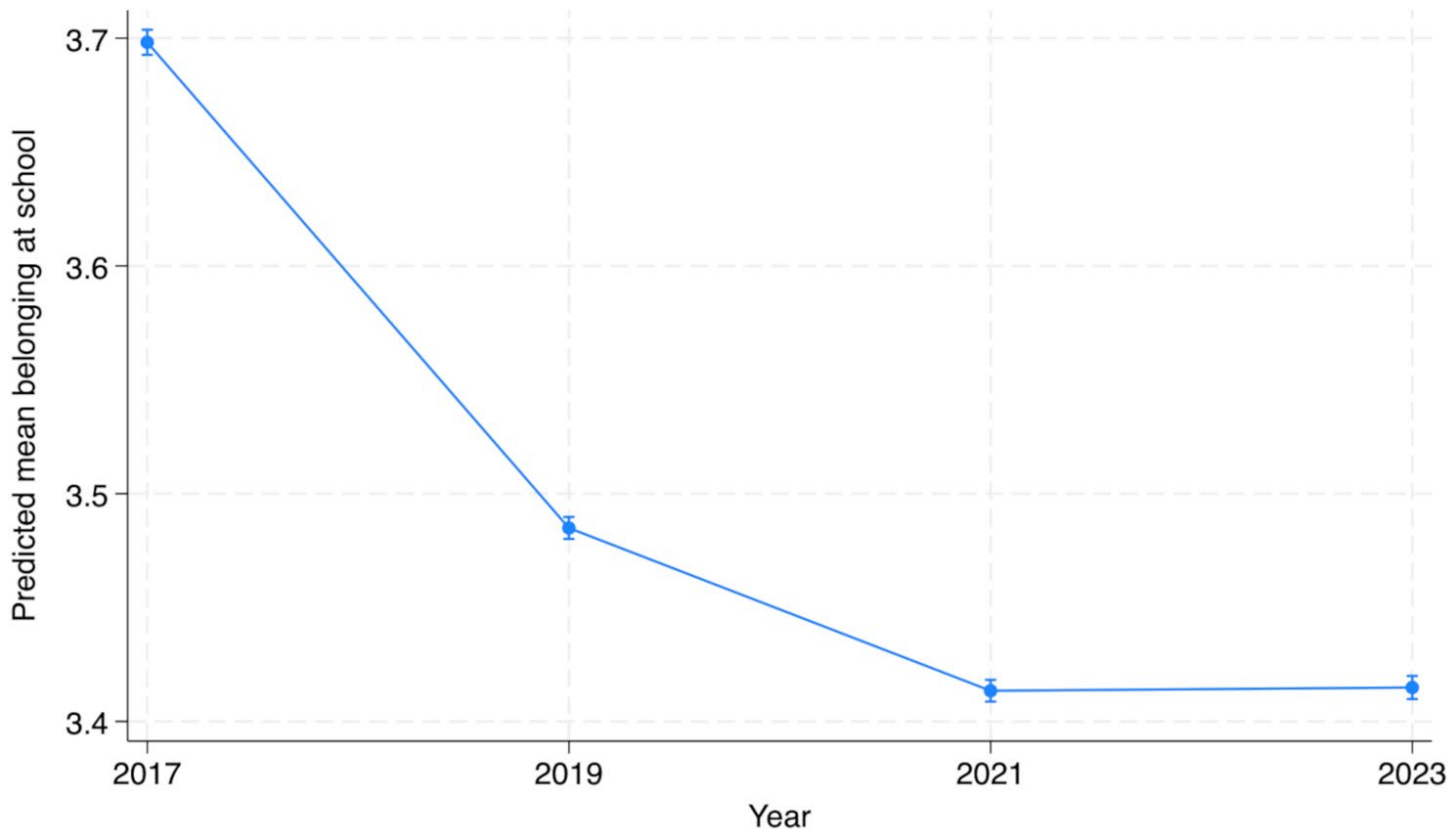
Sense of belonging at school in secondary (lower, upper and vocational) school students in Finland between 2017 and 2023. The estimated mean is shown from a model adjusted for school level, gender, parental education, immigration status of the student and urban/rural location. Spikes indicate the 95% confidence interval

The overall decreasing trend of belonging at school between 2017 and 2021 stopped between 2021 and 2023. Sense of belonging at school declined by 2% between 2019 and 2021 and was followed by a very slight (non-significant) increase between 2021 and 2023. The estimated means were 3.70 in 2017, 3.49 in 2019, 3.41 in 2021 and 3.42 in 2023 (Fig. 3 and Supplementary material 1). Belonging at school was reported more often by boys, students in upper secondary and vocational schools (compared to lower secondary school), schools in more rural locations, students with higher parental education and those with no immigration background compared to those with one foreign-born parent or students and parents born abroad (Supplementary material 1). However, students born in Finland to foreign-born parents reported higher school belonging compared to students with no immigration background.

### Identification of the subgroups (inequalities) in recovery to pre-pandemic levels

The interactions in the multivariable models showed differences in social connectedness by subgroups of students (Table 3). The magnitudes and confidence intervals of the changes between 2019 and 2021 and 2021-23 calculated using margins from the models are shown in Supplementary material 2. In the following, we summarise the key findings.

**Table 3:** Multivariable regressions for number of close friends, loneliness and belonging at school in secondary school students in Finland

	Number of close friends ^a^		Loneliness ^b^		Belonging at school ^c^	
	Model 1	Model 2	Model 1	Model 2	Model 1	Model 2
*Main effects*						
Year of survey (ref = 2017)						
2019	-0.01	0.00	0.00	0.02***	-0.32***	-0.31***
2021	-0.02***	-0.01*	0.11***	0.12***	-0.33***	-0.33***
2023	-0.03***	-0.02***	0.08***	0.11***	-0.36***	-0.39***
School level (ref = lower secondary school)						
Upper secondary	0.00	-0.00	0.09***	0.06***	-0.13***	-0.10***
Vocational	0.05***	0.04	-0.00	0.02***	0.12***	0.16***
Female	-0.00	-0.01*	0.25***	0.27***	-0.41***	-0.35***
Parent(s) with higher education degree	0.03***	0.03***	-0.01***	-0.01***	0.09***	0.08***
Immigration status (ref = Student and parents born in Finland)						
One parent foreign-born^d^	-0.04***	-0.03**	0.05***	0.04***	-0.06***	-0.05***
Born in Finland with foreign-born parents	-0.07***	-0.07**	-0.03***	-0.04***	0.04**	-0.02
Born abroad with foreign-born parents	-0.18***	-0.26***	0.10***	0.13***	-0.10***	-0.24***
Urban-rural (ref = urban)						
Semi-urban	-0.01***	-0.01***	-0.01***	-0.01	0.03***	0.06***
Rural	-0.02***	-0.02***	-0.00	-0.00	0.06***	0.08***
*Wald tests for interactions*:	Wald (df)	Wald (df)	Wald (df)	Wald (df)	Wald (df)	Wald (df)
Year*gender*school level	37.52 (6)***	-	27.08 (6)***	-	59.51 (6)***	-
Year*gender*school level*immigration	-	32.01 (18)*	-	-	-	-
Year*gender*parental education	-	11.21 (3)*	-	-	-	13.85 (3)**
Year*gender*immigration	-	-	-	-	-	-
Year*gender*urban-rural	-	-	-	-	-	16.72 (6)*
Year*school level*parental education	-	-	-	-	-	14.65 (6)*
Year*school level*immigration	-	-	-	-	-	-
Year*school level*urban-rural	-	-	-	29.96 (12)**	-	59.94 (12)***
Year*parental education	-	-	-	-	-	-
Year*immigration	-	-	-	47.02 (9)***	-	130.71 (9)***
Year* urban-rural	-	-	-	-	-	-

Model 1: interactions for year*gender* school level only; Model 2: interactions for year*gender* school level + parental education, immigration or urban-rural indicator

Notice: Unstandardized estimates for the final model shown; higher-order non-significant interactions removed from the model shown; see Supplementary material 1 for the estimates and Wald testing at each step. ^a^ Poisson regression, *n* = 556,754; ^b^ Generalized linear model, *n* = 557,391; ^c^ Generalized linear model, *n* = 556,424; ^d^ In addition to the students with one parent foreign-born, this category also includes students born abroad and a parent born in Finland. df = degrees of freedom. *** *p* < 0.001, ** *p* < 0.01, * *p* < 0.05

In number of close friends, female compared to male gender was in general associated with larger overall declines in number of close friends between 2019 and 2023. However, gender did not appear to play a role in trends between 2021 and 2023 and there were exceptions by other characteristics: the largest recovery (increase) in number of close friends (although with overlapping CIs) was in girls in vocational school who were born in Finland to foreign-born parents. Boys born abroad generally showed the smallest changes in number of friends and were the only other groups that reach the pre-pandemic (2019) levels in number of close friends. Despite the faster recovery (increase) in number of close friends in many students with immigrations background, these groups still reported on average fewer close friends compared to the students without immigration background in 2023. The role of parental education seemed to have a variable association with the trend in having close friends.

In loneliness, the largest overall increase was in students, especially boys, in vocational school in urban locations between 2019 and 2023. Students in upper secondary schools, especially in urban locations, showed somewhat faster recovery (decrease) in loneliness between 2021 and 2023 compared to the school level groups. The largest reduction in feelings of loneliness was in students born abroad. None of the studied groups recovered to the pre-pandemic lower levels of loneliness in 2019.

The largest overall decline in sense of belonging at school was in girls in lower secondary school between 2019 and 2023. Students (both boys and girls) in upper secondary school showed the fastest recovery (increase) in belonging at school between 2021 and 2023. Some groups reached the pre-pandemic levels of sense of school belonging: students in upper secondary school in urban locations and in vocational school in semi-urban locations, boys in upper secondary and vocational schools, and boys in semi-urban and rural locations. However, all these groups apart from the boys in (urban) upper secondary school did not show much change over the whole period 2019–2023. The only group that showed an increased sense of belonging at school in 2023 compared to the pre-pandemic levels in 2019 was students born abroad. Sense of belonging at school was higher in this group compared to the students without immigration background in 2023. Parental education appeared to have mixed associations with the trends in sense of belonging at school.

Apart from a faster overall recovery (increase) in social connectedness between 2021 and 2023 in upper secondary schools and some immigrant groups, the results did not support the assumption that recovery was systematically dependent on the background characteristics. There was little evidence of increasing social inequalities in post-pandemic trends of social connectedness between those subgroups of students thought to be advantaged and disadvantaged.

## Discussion

In summary, the results showed that the declining trend of social connectedness between 2017 and 2021 slowed down in number of close friends and sense of belonging at school between 2021 and 2023. There was a slight recovery in the feelings of loneliness. In some subgroups, such as students in general upper secondary schools and students with immigration background had a faster recovery than other student groups. The patterns of recovery did not point to systematic differences between the groups due to inequalities. The results concerning the research questions are discussed in detail below.

### General trends in social connectedness since 2017 when year 2023 was added

Although the overall declining trend slowed down in all three social connectedness outcomes, the pattern for each was slightly different. These three measures represent different aspects of social connectedness that are relevant in young people’s life. They appear to differ from each other in the ways how and when each of them might be shaped by events such as the pandemic period. Number of close friends kept on declining at a slower rate between 2021 and 2023, while the level of sense of belonging at school stalled in 2023. Feelings of loneliness was the only measure of social connectedness that showed an overall slight improvement (1–2% decrease) between 2021 and 2023. Even feelings of loneliness remained far from the pre-pandemic levels in 2019 and none of the subgroups of students in our study recovered to the pre-pandemic levels of loneliness, unlike in number of close friends and sense of belonging at school where some groups of students either maintained or recovered to the pre-pandemic levels.

The current results add to the previous analysis on the trends up till year 2021 [3], which were in line with some other previous studies showing faster rates of social isolation, loneliness and decreased sense of connectedness during the pandemic [23, 24]. Our study also points to the declining trend of social connectedness in young people that had already started well before 2019 [1–3, 21, 22, 27, 30]. The shape of the change between 2021 and 2023 suggest that in all these three measures the return to “normal” by 2023 meant slower declining, maintaining or some recovering of social connections, but no outright bouncing back.

The slow return to normal has been discussed in the context of school and wellbeing [28, 67–69]. It is possible that when the social restrictions were lifted, the immediate feelings of loneliness were the first to somewhat recover [28], whereas finding new close friendships takes more time and can be challenging, especially when the disruption has been during the sensitive period of adolescence for forming social relationships [11, 13]. Close friendships and sense of school belonging can also be difficult to restore when large parts of society including school have moved to digital and virtual provision of learning and interaction. The current study, as the observational studies in general, cannot rule out the impact of other societal changes during the same period. These can be the increasing use of social media and smartphones, general declining wellbeing and economic trends [1, 27, 31] and changes in national school policies [2, 3]. These factors may have contributed to the declining trends of social connectedness already before the pandemic.

In the era of downward psychosocial wellbeing trends in young people [23, 24, 70–72], there may be some consolation in the finding that the rate of social connectedness has slowed down with some recovery. However, the situation in 2023 is a far cry from the levels of social connectedness in 2017, which was our first measurement point. Keeping in mind that the pandemic restriction measures were directly targeting social connections [73], a larger immediate improvement might have been expected. The current results suggest that lifting the restrictions and returning back to the usual school environment and everyday life have had some positive impact, e.g. a slight overall reduction in loneliness, but generally the recovery of social connections in young people has been slow. In terms of having close friends the trend continued to decline. It has been pointed out previously that when it comes to social connectedness, returning to “normal” after the pandemic may take several years [27]. The reducing number of close friends but some improvement in loneliness may implicate those friendships have been replaced by some other forms of interactions, possibly virtual interactions or relationships with family members.

Whatever the mechanisms, the results are worrying and call for action. It is well-established that social connections are vital for young people’s development and wellbeing [6, 44, 74–76]. They are the building blocks of social capital and networking later in life [32]. If the current generations of young people are missing out the essential social contacts, they have little to build upon later in life. Previous research suggest that increased smartphone and internet use was associated with feelings of loneliness in the cohorts of young people between 2000 and 2018 [1] and replacing social contacts with increased social media and online contacts was associated with poorer wellbeing in young people during the pandemic [77].

The experiences of the students during the pandemic also highlight the dissatisfaction with online school environment [78–80]. However technically well-organised it might have been, online classes lacked the social aspect crucial for human learning. It is therefore surprising to see the current global push to move everyone to an online, virtual and digital world, especially concerning the lives of young people, while claiming to provide a solution to social inclusion [81]. As the Nordic report on the lessons learnt from the pandemic puts it: “youth cannot be lived from a distance” [73]. This is especially problematic as the previous research points that social connections in adolescence have more long-term associations with mental health in the later life than for instance academic achievement [82]. Therefore, the declining and stagnated trends of social connectedness should not be overlooked.

### Identifying subgroups of students whose social connectedness recovered to the pre-pandemic levels and potential inequalities in recovery

The results are in line with consistently higher loneliness in girls compared to boys before and during the pandemic [3, 26, 29], and the trends of increased feelings of loneliness and lower school belonging in girls compared to boys before 2019 [1–3, 21, 54]. However, between 2021 and 2023, gender alone was a lesser defining factor in the changes, whereas school level appeared to play a more important role. For the students in general upper secondary school, the changes were more positive than for those in lower secondary or vocational school. It is important to keep in mind that the starting point for students in general upper secondary school was in most cases lower in 2019 and they experienced a larger decline in school belonginess between 2019 and 2021. The results may point to a greater vulnerability of girls and students in general upper secondary school students to unpredictable changes in school procedures as was the case during the pandemic. Feelings of being connected to school might have been especially at risk due to the pandemic restrictions, as these groups often show higher educational aspirations and prepare for the competition to enter higher education [55].

We found higher levels of social connectedness in students with higher parental educational level and no immigration background in 2019. This is in line with other findings on the differences by socioeconomic and immigrations status [2, 22]. We also found that the diminishing differences between the advantaged and less advantaged groups when looking at the trends up to 2021 [3] continued further to 2023. The current results suggest that there were changes that brought these groups closer to each other. This was mainly due to that the expected protective factors such as higher parental education and no immigration background were not associated with a faster recovery, but often with the opposite. In most of the cases, parental education appeared to have little association with the changes between 2021 and 2023, possibly pointing to the waning role of family protective factors after 2021.

The role of urban-rural location appeared to also become less clear between 2021 and 2023. This seemed to continue the trend that the differences by location started to disappear between 2017 and 2021 [3]. Even though the rural schools were less affected by the pandemic-related social restrictions [83, 84], there seemed to be little difference in the trends of social connectedness between the students in urban compared to rural locations during this period. Although the urban-rural location appeared to become a less defining factor in the trends of social connectedness, the results still supported the pattern that urban environment was associated with having more close friends, whereas rural locations were characterised by a stronger sense of belonging at school [35–37]. Schools in rural communities tend to be smaller, with fewer students and smaller class sizes. This can shape students’ social connectedness differently than in urban locations.

In terms of immigration status, some groups made considerable improvement in social connectedness between 2021 and 2023. For instance, foreign-born students in lower secondary school and girls born in Finland to foreign-born parents showed better maintenance or increases in number of close friends, while foreign-born students showed decreased feelings of loneliness and increased belonging at school between 2021 and 2023, compared to those with no immigration background. The results point to a continuing trend started already between 2017 and 2021 [3] that the differences in social connectedness by immigration status reversed further between 2021 and 2023. The results of declining and even reversing trend differences by immigration status and urban-rural location are in line with the findings in a Danish data between 1991 and 2014 [36]. The Danish study showed decreasing social inequality in loneliness because of those with more advantageous background getting lonelier.

There may be several reasons for declining social connectedness and narrowing social gradient. Although it is possible that the institutional and societal events may hit those with the least advantaged socioeconomic position and least resources hardest [2], it is also possible that they affect more those families who have more socioeconomic resources and higher expectations of the future opportunities [36]. Social upheavals may particularly affect those who have more to lose. For instance, the opportunities for social interactions in paid activities and hobbies which were closed during the pandemic would have impacted more those young people whose families would have been able to afford the activities. Even school-related extra curriculum activities, which mostly are free, are often joined more by those students who come from a more advantaged background, and the uptake of these activities has not been the same as before the pandemic [85]. In Finland, there were initiatives to provide for free activities to children and young people during the pandemic [73]. Many of the organised activities were either outdoor or online which cannot necessarily replace the type of interaction young people preferred. The same report [73] reveals that young people’s rights for participation and their own voice was mostly overlooked, especially during the early stages of the pandemic. Generally, children’s rights were not considered during the pandemic, and their voices were not heard in decision making [47].

The enhanced school support in secondary schools in Finland during the pandemic may have however had a positive impact although not directly targeting social connectedness. Students’ coping was supported by promoting psychological wellbeing, increasing teacher support and interaction, and strengthening student welfare services, particularly for those in vulnerable positions [86]. Learning support was enhanced, and students’ self-directed learning was encouraged through various pedagogical approaches. Special arrangements were implemented for students in need of special support as well as for those with immigrant backgrounds, which could have indirectly boost wider wellbeing such as social connectedness of immigrant students. This needs-based approach based on the student may be more beneficial than for instance the support based on the parents’ keyworker position that was used in some other countries. A recent review [69] mentions that secondary school students perceived that the selected students in keyworker families were getting better support during the pandemic and felt that all students should have deserved a better-quality support.

What appears to be declining inequalities may reflect larger macro-level changes that may have had more impact on those with more advantageous positions. The question is whether the measures taken to help the recovery after the pandemic have ignored most of the students, the so-called “mainstream” young people. As we see from the distributions of individual and school characteristics in the current study, those with stagnated trends of social connectedness form the majority of the students - i.e. most students lived in a family where at least one parent had higher education degree, most students were not immigrants. The social position of these groups may have been shifted related to reduced feelings of belonging, in this case ‘school belonging’.

These feelings may reflect changes in experiencing belonging across social, national, and cultural borders [18], or being ‘at-home’ in the present society [19, 20]. From 2017 to 2021, most of the students viewed themselves less often as an important member of the classroom and school community [3]. By 2023, the different rate of changes by immigration status had changed the order so that students with foreign background exhibited a higher sense of school belonging than students with no immigration background. Immigrant families may pose several resources such as having healthy and intact families, strong work ethic and aspirations, community cohesion and greater resilience in the strategies to acquire and maintain social ties [39–41] which may help to navigate in uncertain and changing environments, such as the changes during the pandemic. Nonetheless, foreign-born students overall reported to have fewer close friends. There may be factors related to school composition, language skills and age at arrival in addition to resources related to the immigration experience that shape social connectedness.

The result may also reflect wider social and cultural shifts influenced by neoliberalism and identity politics that has redefined who is vulnerable, and in terms of social settings who is in and out [87]. In this context, the individual is eventually required to fit into the system rather than that the system is modified to serve the individual. This can result in “othering” and alienation, usually understood to affect groups that do not align with the dominant culture attached to minority groups [88], but in this case could make school a place where the majority does not feel they are an important, valued member. Turning the focus on vulnerability during the pandemic can be also seen in normalising social isolation [31]. Although a large bulk of literature during and after the pandemic highlighted widening inequity and increased vulnerability which many cases are real and justified, there should be a balanced attention to the overarching trends. Only few studies looked at the changes over time in the general population of young people [89]. Rather than finding the victims and labelling the vulnerable (which would be the most students in this case), it is important to focus on the strengths and how to build them in the population level. The take-home message from this is that the pandemic-induced social changes had a wide-spread impact, and therefore the focus needs to be on the whole generation [68].

### Limitations of the study

Although the study includes a large representative sample of secondary school students in Finland, the survey focuses on health-related topics and has a limited set of socioeconomic variables and measures of social connectedness. Use of short single- or two-item measures may not fully capture the quality or depth of social connectedness and may limit their construct validity compared to multi-item scales. It is possible that for instance the interpretation of what close friendship and talking confidentially about almost everything mean may vary from one person to another. Because of the selected measures used, some of the findings may be difficult to compare with other studies using more comprehensive or different measures for social connectedness or individual and environmental background factors. Parental education and immigration status of the student were based on the students’ self-reports, which may contain reporting error [56]. To fill in the survey, the student needed sufficient language skills in Finnish or Swedish (two official languages in Finland) or any of the three other languages the questionnaire was translated into (Russian, English, North Sami). The questionnaire was also available in plain Finnish and Swedish. Despite these options, it is possible that some newly immigrated students or other students with language barriers were not included. This may impact the representativeness of the sample in some of the subgroups and may over- or underestimate the levels of social connectedness in these groups.

The category ‘partial foreign-born background‘ was mixed including primarily students with one parent foreign-born but also those born abroad and one parent born in Finland. The latter of the subgroups was a quarter of the size of this category. Because of its small size this subgroup could not be analysed as a separate category. It is possible that the combined category impacted the results. However, there were no differences between these two subgroups in terms of social connectedness outcomes. Excluding the smaller subgroup (1.6% of the total sample) did not change the overall results for this immigration category (results not shown).

The participation rates varied by year in lower and general upper secondary schools. In vocational schools they also varied by the field of study and were lower than in lower and general upper secondary schools. In vocational schools, the age limit of less than 21 years for inclusion made it challenging to reach and motivate the students in the target group to participate when older students didn’t participate. Those older than 18 years made up 2.3% of the students in vocational schools. Excluding them from the analysis did not change the findings (results not shown). Moreover, some students were doing work placements as part of the vocational qualification and were not available at the time of the data collection. The selection of the vocational school students may have impacted the results. It is however important to note that all the models were adjusted for the key background factors, and therefore any differences because of them were adjusted for in the models. The study investigated cross-sectional cohort trends at the population level. By design, it does not follow up longitudinal changes within individuals. It also does not contain a control group, so the changes between 2019 and 2021 and between 2021 and 2023 may be attributed to the pandemic or other events in the same period.

## Conclusions

Our findings suggest that the declining trend in social connectedness in secondary school students since 2017 and especially during the pandemic (between 2019 and 2023) has slowed down by 2023. Overall, there is very little if any recovery. However, there were some subgroups of student whose social connectedness bounced back: especially evident in sense of belonging at school in students in general upper secondary school and in some groups of students with immigration background. The protective role of higher parental education and urban-rural location (protective depending on the outcome) seemed to become increasingly ambiguous in the changes of social connectedness between 2021 and 2023, continuing the trend found between 2017 and 2021 [3]. The patterns suggest that there were no differences between the disadvantaged and advantaged groups or that the groups often considered to be disadvantaged such as students with foreign background recovered faster than their more advantaged counterparts by 2023. Moreover, our results also showed that gender although still an important factor in the levels of social connectedness made little difference in the rate of change between 2021 and 2023.

Feelings of social disconnect to others is a serious challenge for young people’s mental and physical wellbeing, increase social inequality and enforce further erosion of social capital in society [5, 6, 8, 90]. Therefore, the promotion of child and youth wellbeing through encouraging close friends, decreasing loneliness and strengthening sense of belonging to learning groups and school is vital. In many countries, including Finland, the government has published a national action plan for reducing loneliness and increasing social connections [91]. There may be a range of school-specific interventions related to changes in the national core curricula, education practices, students’ wellbeing related services, interprofessional training, service integration and public policy-related formulations.

The wider community may also be involved. To increase social connections asset-based approaches could be applied to strengthen communities, enhance volunteering, peer roles collaborations and partnerships and utilise access to community resources [92]. These approaches have a potential to increase social connectedness, empowerment, participation, cohesion, resilience and social capital not only in the school environment, but in the nested systems around it. These approaches, which often involved young people, proved positive impact during the pandemic in community settings in England [93]. Asset-based approach has been designed to reaching out those groups of people who may otherwise been left out, e.g. those with low socioeconomic status or immigrants. Both groups are diverse in their background and social resources. This approach may benefit those immigrant students who lack the social connections in the new country [94, 95]. However, some of those with immigration background may already have the networks to support them. The interesting question is whether asset-based approach would be a useful tool to target the sluggish recovery in the “mainstream” students. According to our findings, those with no immigration background showed declining social connectedness already years before the pandemic - a pattern which has also been seen globally [31]. This suggests eroding communities and supporting networks well before the pandemic, potentially leading to a break-down of social cohesion for those who were assumed to be the “advantaged” or “all right”. There may be a need for a community-wide approach supporting both its established and new members with the ways that are appropriate to each. After side-lining young people’s interests and rights to social life in decision making during the pandemic [47, 73], it is important to listen to young peoples’ own voice and respect their needs now and in the future.

## Supplementary Information

Below is the link to the electronic supplementary material.

Supplementary material 1: Stata code and outputs for the regression models on social connectedness

Supplementary material 2: Supplementary tables for estimated means and changes in social connectedness between 2019–2023

Supplementary material 3: Data diagnostics and missing data

## Data Availability

Finnish Institute for Health and Welfare (THL) has collected the data and has the rights for the data. THL is committed to complying with the established code of conduct in its field. THL produces public statistical reports and interactive reports, but the data is confidential. Researchers can apply the data from Findata (https://findata.fi/en/) or the 2019 data from Finnish Social Science Data Archive (https://www.fsd.tuni.fi/en/).
