# Supplementary material for: Is social connectedness still in decline after the Covid-19 pandemic? Cohort trends in secondary school students in Finland between 2017 and 2023
Source: BMC Psychol. 2025 Sep 29;13:1071. doi: 10.1186/s40359-025-03394-5 (PMC12482009; doi:10.1186/s40359-025-03394-5)
Supplement: Supplementary file 1 — Supplementary material 1: Stata code and outputs for the regression models on social connectedness [file 40359_2025_3394_MOESM1_ESM.doc]

Supplementary material 1.

Stata code and outputs for the regression models on social connectedness

Table of Contents

[1. Description on the online resource 1](#__RefHeading___Toc196214601)

[2. Multivariable regressions with main effects only, unstandardized estimates and margins for study year 3](#__RefHeading___Toc196214603)

[2.1. Number of close friends 3](#__RefHeading___Toc196214604)

[2.2. Loneliness 3](#__RefHeading___Toc196214605)

[2.3. Belonging at school 4](#__RefHeading___Toc196214606)

[3. Multivariable regressions with interactions, unstandardized estimates and margins 5](#__RefHeading___Toc196214607)

[3.1. Number of close friends 5](#__RefHeading___Toc196214608)

[3.2. Loneliness 23](#__RefHeading___Toc196214609)

[3.3. Belonging at school 36](#__RefHeading___Toc196214610)

# 1. Description on the online resource

This file shows the original model results as Stata outputs.

* Section 2 shows the multivariable regressions with unstandardized estimates for three outcomes (number of close friends, loneliness and belonging at school). For each outcome, the main effect models with the predicted margins for study year are shown (2.1., 2.2. and 2.3). These models were used for the general illustration of the trends over time (”**General trends in social connectedness since 2017 when year 2023 was added**” in the results section of the main text and **Figures 1-3**).

* Section 3 shows the models with the interactions. First, the interaction year*gender*school level was added to the models (3.1., 3.2. and 3.3.) and the predicted margins were calculated for the significant interaction (or lower-level interaction with year) in the model. Second, the interactions of parental education, urban-rural location of the school and immigration status of the student were added to the model with any relevant interactions with year, and gender or school level and the predicted margins were calculated for the significant interaction in the final model. The models are presented in the result section of the main text (“**Identification of the subgroups (inequalities) in recovery to pre-pandemic levels”** and **Table 3**).

# 2. Multivariable regressions with main effects only, unstandardized estimates and margins for study year

## 2.1. Number of close friends

. poisson closefriend3 i.year i.gender i.schoollevel i.degree i.urbanrural i.immigrant, vce(robust)

Iteration 0: Log pseudolikelihood = -872868.18

Iteration 1: Log pseudolikelihood = -872868.18

Poisson regression Number of obs = 556,754

Wald chi2(12) = 5278.69

Prob > chi2 = 0.0000

Log pseudolikelihood = -872868.18 Pseudo R2 = 0.0017

--------------------------------------------------------------------------------------------------------

| Robust

closefriend3 | Coefficient std. err. z P>|z| [95% conf. interval]

---------------------------------------+----------------------------------------------------------------

year |

2019 | -.0156776 .0017408 -9.01 0.000 -.0190895 -.0122657

2021 | -.0517796 .0017591 -29.44 0.000 -.0552273 -.0483319

2023 | -.0656109 .0018285 -35.88 0.000 -.0691946 -.0620271

|

gender |

girl | -.0340052 .001262 -26.95 0.000 -.0364786 -.0315318

|

schoollevel |

upper secondary | .0148892 .0014266 10.44 0.000 .012093 .0176853

vocational | .0136809 .0018305 7.47 0.000 .0100931 .0172687

|

1.degree | .031436 .0013147 23.91 0.000 .0288592 .0340127

|

urbanrural |

semi-urban | -.0088919 .0017024 -5.22 0.000 -.0122286 -.0055552

rural | -.0240392 .0020486 -11.73 0.000 -.0280544 -.020024

|

immigrant |

One foreign-born parent | -.0390663 .0025284 -15.45 0.000 -.0440218 -.0341109

Born in Finland, foreign-born parents | -.0667399 .0049261 -13.55 0.000 -.0763949 -.057085

Student and parents born abroad | -.1791872 .004676 -38.32 0.000 -.1883519 -.1700224

|

_cons | .8123805 .0018142 447.78 0.000 .8088247 .8159364

--------------------------------------------------------------------------------------------------------

. margins year

Predictive margins Number of obs = 556,754

Model VCE: Robust

Expression: Predicted number of events, predict()

------------------------------------------------------------------------------

| Delta-method

| Margin std. err. z P>|z| [95% conf. interval]

-------------+----------------------------------------------------------------

year |

2017 | 2.235753 .0028211 792.52 0.000 2.230224 2.241282

2019 | 2.200975 .0026419 833.10 0.000 2.195797 2.206153

2021 | 2.122933 .0025963 817.68 0.000 2.117844 2.128022

2023 | 2.093772 .0027569 759.46 0.000 2.088369 2.099176

------------------------------------------------------------------------------

## 2.2. Loneliness

. glm loneliness i.year i.gender i.schoollevel i.degree i.urbanrural i.immigrant, family(gamma) link (log) vce(robust) nolog

Generalized linear models Number of obs = 557,391

Optimization : ML Residual df = 557,378

Scale parameter = .1958252

Deviance = 113868.3582 (1/df) Deviance = .2042929

Pearson = 109148.6499 (1/df) Pearson = .1958252

Variance function: V(u) = u^2 [Gamma]

Link function : g(u) = ln(u) [Log]

AIC = 3.667761

Log pseudolikelihood = -1022175.427 BIC = -7260812

--------------------------------------------------------------------------------------------------------

| Robust

loneliness | Coefficient std. err. z P>|z| [95% conf. interval]

---------------------------------------+----------------------------------------------------------------

year |

2019 | .0241614 .0017635 13.70 0.000 .0207051 .0276178

2021 | .1285058 .0017175 74.82 0.000 .1251396 .131872

2023 | .1062519 .001764 60.23 0.000 .1027945 .1097094

|

gender |

girl | .2686721 .0012057 222.84 0.000 .266309 .2710352

|

schoollevel |

upper secondary | .0581069 .0013095 44.37 0.000 .0555404 .0606735

vocational | .0251593 .0018254 13.78 0.000 .0215816 .028737

|

1.degree | -.0100204 .0012455 -8.05 0.000 -.0124617 -.0075792

|

urbanrural |

semi-urban | -.0127527 .0016276 -7.84 0.000 -.0159427 -.0095627

rural | -.0023678 .0018927 -1.25 0.211 -.0060774 .0013418

|

immigrant |

One foreign-born parent | .0455711 .0022746 20.03 0.000 .0411129 .0500292

Born in Finland, foreign-born parents | -.0250333 .0047795 -5.24 0.000 -.0344011 -.0156656

Student and parents born abroad | .0946633 .0040486 23.38 0.000 .0867281 .1025985

|

_cons | .6049241 .0018341 329.83 0.000 .6013294 .6085188

--------------------------------------------------------------------------------------------------------

. margins year

Predictive margins Number of obs = 557,391

Model VCE: Robust

Expression: Predicted mean loneliness, predict()

------------------------------------------------------------------------------

| Delta-method

| Margin std. err. z P>|z| [95% conf. interval]

-------------+----------------------------------------------------------------

year |

2017 | 2.172272 .002836 765.97 0.000 2.166714 2.177831

2019 | 2.225397 .0026147 851.11 0.000 2.220272 2.230521

2021 | 2.470151 .0027161 909.44 0.000 2.464828 2.475475

2023 | 2.415788 .0028154 858.07 0.000 2.41027 2.421306

------------------------------------------------------------------------------

## 2.3. Belonging at school

. glm belonging i.year i.gender i.schoollevel i.degree i.urbanrural i.immigrant, family(gaussian) link (identity) vce(robust

> ) nolog

Generalized linear models Number of obs = 556,424

Optimization : ML Residual df = 556,411

Scale parameter = .9119939

Deviance = 507443.4176 (1/df) Deviance = .9119939

Pearson = 507443.4176 (1/df) Pearson = .9119939

Variance function: V(u) = 1 [Gaussian]

Link function : g(u) = u [Identity]

AIC = 2.745778

Log pseudolikelihood = -763895.5036 BIC = -6853477

--------------------------------------------------------------------------------------------------------

| Robust

belonging | Coefficient std. err. z P>|z| [95% conf. interval]

---------------------------------------+----------------------------------------------------------------

year |

2019 | -.2132241 .0037283 -57.19 0.000 -.2205315 -.2059167

2021 | -.2846802 .0037263 -76.40 0.000 -.2919836 -.2773768

2023 | -.2832619 .0038304 -73.95 0.000 -.2907694 -.2757544

|

gender |

girl | -.3537608 .002585 -136.85 0.000 -.3588274 -.3486942

|

schoollevel |

upper secondary | .0734015 .0029273 25.07 0.000 .067664 .0791389

vocational | .2038861 .0037067 55.00 0.000 .1966211 .2111511

|

1.degree | .091996 .0026893 34.21 0.000 .0867251 .097267

|

urbanrural |

semi-urban | .0301299 .0034709 8.68 0.000 .023327 .0369327

rural | .0585494 .0041401 14.14 0.000 .0504351 .0666638

|

immigrant |

One foreign-born parent | -.0549985 .0050822 -10.82 0.000 -.0649595 -.0450376

Born in Finland, foreign-born parents | .0431654 .0095064 4.54 0.000 .0245331 .0617976

Student and parents born abroad | -.0932426 .0084487 -11.04 0.000 -.1098018 -.0766834

|

_cons | 3.775833 .0038414 982.93 0.000 3.768304 3.783362

--------------------------------------------------------------------------------------------------------

. margins year

Predictive margins Number of obs = 556,424

Model VCE: Robust

Expression: Predicted mean belonging, predict()

------------------------------------------------------------------------------

| Delta-method

| Margin std. err. z P>|z| [95% conf. interval]

-------------+----------------------------------------------------------------

year |

2017 | 3.698152 .0028113 1315.48 0.000 3.692642 3.703662

2019 | 3.484928 .0024564 1418.71 0.000 3.480113 3.489742

2021 | 3.413472 .0024435 1396.97 0.000 3.408683 3.418261

2023 | 3.41489 .0025866 1320.24 0.000 3.40982 3.41996

------------------------------------------------------------------------------

# 3. Multivariable regressions with interactions, unstandardized estimates and margins

## 3.1. Number of close friends

. poisson closefriend3 i.year##i.gender##i.schoollevel i.degree i.urbanrural i.immigrant, vce(robust)

Iteration 0: Log pseudolikelihood = -872659.69

Iteration 1: Log pseudolikelihood = -872659.69

Poisson regression Number of obs = 556,754

Wald chi2(29) = 6161.49

Prob > chi2 = 0.0000

Log pseudolikelihood = -872659.69 Pseudo R2 = 0.0019

--------------------------------------------------------------------------------------------------------

| Robust

closefriend3 | Coefficient std. err. z P>|z| [95% conf. interval]

---------------------------------------+----------------------------------------------------------------

year |

2019 | -.0052001 .0036405 -1.43 0.153 -.0123353 .0019352

2021 | -.0169724 .0036087 -4.70 0.000 -.0240452 -.0098996

2023 | -.0307594 .0037067 -8.30 0.000 -.0380243 -.0234945

|

gender |

girl | -.0036952 .003544 -1.04 0.297 -.0106412 .0032509

|

year#gender |

2019#girl | -.0102499 .0048351 -2.12 0.034 -.0197265 -.0007734

2021#girl | -.06046 .0048554 -12.45 0.000 -.0699765 -.0509435

2023#girl | -.0711504 .0050332 -14.14 0.000 -.0810152 -.0612856

|

schoollevel |

upper secondary | .0000541 .0047467 0.01 0.991 -.0092493 .0093574

vocational | .0466844 .0045128 10.34 0.000 .0378394 .0555294

|

year#schoollevel |

2019#upper secondary | -.0107811 .0064281 -1.68 0.094 -.02338 .0018178

2019#vocational | -.0223898 .0065428 -3.42 0.001 -.0352134 -.0095662

2021#upper secondary | -.0144532 .0063906 -2.26 0.024 -.0269784 -.0019279

2021#vocational | -.0194731 .0066216 -2.94 0.003 -.0324513 -.0064949

2023#upper secondary | -.0075898 .0065273 -1.16 0.245 -.0203832 .0052036

2023#vocational | -.0244584 .0070102 -3.49 0.000 -.0381982 -.0107185

|

gender#schoollevel |

girl#upper secondary | .0227737 .0060276 3.78 0.000 .0109599 .0345875

girl#vocational | -.0506395 .0065994 -7.67 0.000 -.0635741 -.0377048

|

year#gender#schoollevel |

2019#girl#upper secondary | .0059141 .0081872 0.72 0.470 -.0101325 .0219607

2019#girl#vocational | .0129028 .0096325 1.34 0.180 -.0059765 .031782

2021#girl#upper secondary | .0259491 .0081982 3.17 0.002 .009881 .0420173

2021#girl#vocational | -.0056536 .0100093 -0.56 0.572 -.0252714 .0139642

2023#girl#upper secondary | .0374786 .00844 4.44 0.000 .0209365 .0540206

2023#girl#vocational | .0309162 .0105126 2.94 0.003 .0103118 .0515206

|

1.degree | .0318399 .001314 24.23 0.000 .0292646 .0344152

|

urbanrural |

semi-urban | -.0092198 .0017018 -5.42 0.000 -.0125553 -.0058843

rural | -.0239319 .0020474 -11.69 0.000 -.0279448 -.0199191

|

immigrant |

One foreign-born parent | -.0388323 .0025278 -15.36 0.000 -.0437868 -.0338779

Born in Finland, foreign-born parents | -.0667783 .0049243 -13.56 0.000 -.0764297 -.0571268

Student and parents born abroad | -.1796874 .0046759 -38.43 0.000 -.188852 -.1705228

|

_cons | .7945608 .0028344 280.33 0.000 .7890055 .800116

--------------------------------------------------------------------------------------------------------

.

. testparm year#gender#schoollevel

( 1) [closefriend3]2019.year#2.gender#20.schoollevel = 0

( 2) [closefriend3]2019.year#2.gender#30.schoollevel = 0

( 3) [closefriend3]2021.year#2.gender#20.schoollevel = 0

( 4) [closefriend3]2021.year#2.gender#30.schoollevel = 0

( 5) [closefriend3]2023.year#2.gender#20.schoollevel = 0

( 6) [closefriend3]2023.year#2.gender#30.schoollevel = 0

chi2( 6) = 37.52

Prob > chi2 = 0.0000

. margins year#gender#schoollevel

Predictive margins Number of obs = 556,754

Model VCE: Robust

Expression: Predicted number of events, predict()

--------------------------------------------------------------------------------------------

| Delta-method

| Margin std. err. z P>|z| [95% conf. interval]

---------------------------+----------------------------------------------------------------

year#gender#schoollevel |

2017#boy#lower secondary | 2.221698 .005959 372.83 0.000 2.210018 2.233377

2017#boy#upper secondary | 2.221818 .0086959 255.50 0.000 2.204774 2.238862

2017#boy#vocational | 2.327876 .0084512 275.45 0.000 2.311312 2.34444

2017#girl#lower secondary | 2.213503 .0051374 430.86 0.000 2.203434 2.223572

2017#girl#upper secondary | 2.264614 .0066024 343.00 0.000 2.251673 2.277554

2017#girl#vocational | 2.204766 .0093685 235.34 0.000 2.186404 2.223128

2019#boy#lower secondary | 2.210175 .00544 406.28 0.000 2.199512 2.220837

2019#boy#upper secondary | 2.186593 .0078359 279.05 0.000 2.171235 2.201951

2019#boy#vocational | 2.264528 .0092164 245.71 0.000 2.246464 2.282592

2019#girl#lower secondary | 2.179568 .0047549 458.38 0.000 2.170248 2.188887

2019#girl#upper secondary | 2.219068 .0059207 374.80 0.000 2.207464 2.230672

2019#girl#vocational | 2.150466 .0101015 212.89 0.000 2.130667 2.170264

2021#boy#lower secondary | 2.184308 .0052698 414.49 0.000 2.17398 2.194637

2021#boy#upper secondary | 2.153082 .007647 281.56 0.000 2.138094 2.168069

2021#boy#vocational | 2.244562 .0094764 236.86 0.000 2.225989 2.263136

2021#girl#lower secondary | 2.048574 .0046673 438.92 0.000 2.039427 2.057722

2021#girl#upper secondary | 2.12011 .005751 368.65 0.000 2.108838 2.131381

2021#girl#vocational | 1.989856 .0105263 189.04 0.000 1.969225 2.010487

2023#boy#lower secondary | 2.1544 .0055067 391.23 0.000 2.143607 2.165193

2023#boy#upper secondary | 2.138226 .0079077 270.40 0.000 2.122727 2.153725

2023#boy#vocational | 2.20282 .0104178 211.45 0.000 2.182401 2.223239

2023#girl#lower secondary | 1.999039 .004991 400.53 0.000 1.989257 2.008822

2023#girl#upper secondary | 2.107249 .0061775 341.12 0.000 2.095141 2.119357

2023#girl#vocational | 2.004049 .0113261 176.94 0.000 1.98185 2.026248

--------------------------------------------------------------------------------------------

.

. poisson closefriend3 i.year##i.schoollevel##i.gender##i.degree i.urbanrural i.immigrant, vce(robust)

Iteration 0: Log pseudolikelihood = -872633.06

Iteration 1: Log pseudolikelihood = -872633.06

Poisson regression Number of obs = 556,754

Wald chi2(52) = 6315.31

Prob > chi2 = 0.0000

Log pseudolikelihood = -872633.06 Pseudo R2 = 0.0019

--------------------------------------------------------------------------------------------------------

| Robust

closefriend3 | Coefficient std. err. z P>|z| [95% conf. interval]

---------------------------------------+----------------------------------------------------------------

year |

2019 | -.0013046 .0053326 -0.24 0.807 -.0117562 .009147

2021 | -.012736 .0053254 -2.39 0.017 -.0231735 -.0022984

2023 | -.0242404 .0055208 -4.39 0.000 -.0350609 -.0134199

|

schoollevel |

upper secondary | -.0148527 .0081715 -1.82 0.069 -.0308685 .001163

vocational | .0401707 .0059728 6.73 0.000 .0284643 .0518772

|

year#schoollevel |

2019#upper secondary | .0001594 .0113416 0.01 0.989 -.0220696 .0223885

2019#vocational | -.0135748 .0086987 -1.56 0.119 -.0306239 .0034743

2021#upper secondary | -.0233088 .0116635 -2.00 0.046 -.0461688 -.0004487

2021#vocational | -.0181492 .0088915 -2.04 0.041 -.0355763 -.0007221

2023#upper secondary | -.0246926 .0121783 -2.03 0.043 -.0485616 -.0008236

2023#vocational | -.0257089 .0095808 -2.68 0.007 -.0444869 -.006931

|

gender |

girl | -.0096398 .005046 -1.91 0.056 -.0195299 .0002502

|

year#gender |

2019#girl | -.0194924 .0070828 -2.75 0.006 -.0333744 -.0056105

2021#girl | -.0622206 .0071533 -8.70 0.000 -.0762408 -.0482003

2023#girl | -.0785472 .007527 -10.44 0.000 -.0932999 -.0637945

|

schoollevel#gender |

upper secondary#girl | .0331059 .01004 3.30 0.001 .0134279 .0527838

vocational#girl | -.0445899 .0085415 -5.22 0.000 -.061331 -.0278488

|

year#schoollevel#gender |

2019#upper secondary#girl | -.0051203 .0139979 -0.37 0.715 -.0325557 .0223151

2019#vocational#girl | .0106617 .0125448 0.85 0.395 -.0139256 .0352489

2021#upper secondary#girl | .0249686 .0143899 1.74 0.083 -.0032351 .0531724

2021#vocational#girl | -.0026979 .0130914 -0.21 0.837 -.0283566 .0229608

2023#upper secondary#girl | .0487838 .0152136 3.21 0.001 .0189657 .0786018

2023#vocational#girl | .0370665 .0140834 2.63 0.008 .0094635 .0646695

|

1.degree | .0225413 .0053665 4.20 0.000 .0120232 .0330593

|

year#degree |

2019 1 | -.0063087 .0072986 -0.86 0.387 -.0206137 .0079964

2021 1 | -.0065076 .0072457 -0.90 0.369 -.020709 .0076938

2023 1 | -.0100397 .0074581 -1.35 0.178 -.0246574 .004578

|

schoollevel#degree |

upper secondary#1 | .0245467 .0101102 2.43 0.015 .0047311 .0443623

vocational#1 | .0147522 .0091967 1.60 0.109 -.003273 .0327773

|

year#schoollevel#degree |

2019#upper secondary#1 | -.0152395 .0138688 -1.10 0.272 -.0424219 .011943

2019#vocational#1 | -.0272243 .0133845 -2.03 0.042 -.0534574 -.0009913

2021#upper secondary#1 | .0111633 .0140633 0.79 0.427 -.0164003 .038727

2021#vocational#1 | -.0083046 .0134818 -0.62 0.538 -.0347285 .0181193

2023#upper secondary#1 | .0219684 .0145598 1.51 0.131 -.0065683 .0505051

2023#vocational#1 | -.0031178 .014199 -0.22 0.826 -.0309474 .0247118

|

gender#degree |

girl#1 | .0120009 .0070866 1.69 0.090 -.0018886 .0258905

|

year#gender#degree |

2019#girl#1 | .0158517 .0097011 1.63 0.102 -.0031622 .0348656

2021#girl#1 | .0017408 .0097557 0.18 0.858 -.0173799 .0208615

2023#girl#1 | .0110488 .0101502 1.09 0.276 -.0088453 .0309429

|

schoollevel#gender#degree |

upper secondary#girl#1 | -.0176156 .0126619 -1.39 0.164 -.0424324 .0072012

vocational#girl#1 | -.011658 .0136657 -0.85 0.394 -.0384422 .0151262

|

year#schoollevel#gender#degree |

2019#upper secondary#girl#1 | .0133562 .0173902 0.77 0.442 -.0207279 .0474404

2019#vocational#girl#1 | .0136227 .0199549 0.68 0.495 -.0254883 .0527336

2021#upper secondary#girl#1 | .0032215 .0176721 0.18 0.855 -.0314152 .0378583

2021#vocational#girl#1 | -.0070234 .0206832 -0.34 0.734 -.0475617 .033515

2023#upper secondary#girl#1 | -.0152702 .0184673 -0.83 0.408 -.0514654 .020925

2023#vocational#girl#1 | -.0092685 .0214903 -0.43 0.666 -.0513888 .0328518

|

urbanrural |

semi-urban | -.0092118 .001702 -5.41 0.000 -.0125477 -.0058759

rural | -.0238453 .0020487 -11.64 0.000 -.0278608 -.0198299

|

immigrant |

One foreign-born parent | -.0386887 .0025279 -15.30 0.000 -.0436433 -.0337341

Born in Finland, foreign-born parents | -.0662345 .004927 -13.44 0.000 -.0758913 -.0565778

Student and parents born abroad | -.1794892 .0046776 -38.37 0.000 -.188657 -.1703213

|

_cons | .7991506 .0038986 204.98 0.000 .7915094 .8067917

--------------------------------------------------------------------------------------------------------

. testparm year#schoollevel#gender#degree

( 1) [closefriend3]2019.year#20.schoollevel#2.gender#1.degree = 0

( 2) [closefriend3]2019.year#30.schoollevel#2.gender#1.degree = 0

( 3) [closefriend3]2021.year#20.schoollevel#2.gender#1.degree = 0

( 4) [closefriend3]2021.year#30.schoollevel#2.gender#1.degree = 0

( 5) [closefriend3]2023.year#20.schoollevel#2.gender#1.degree = 0

( 6) [closefriend3]2023.year#30.schoollevel#2.gender#1.degree = 0

chi2( 6) = 3.40

Prob > chi2 = 0.7571

.

. poisson closefriend3 i.year##i.schoollevel##i.gender##i.urbanrural i.degree i.immigrant, vce(robust)

Iteration 0: Log pseudolikelihood = -872619.08

Iteration 1: Log pseudolikelihood = -872619.08

Poisson regression Number of obs = 556,754

Wald chi2(75) = 6349.53

Prob > chi2 = 0.0000

Log pseudolikelihood = -872619.08 Pseudo R2 = 0.0020

--------------------------------------------------------------------------------------------------------

| Robust

closefriend3 | Coefficient std. err. z P>|z| [95% conf. interval]

---------------------------------------+----------------------------------------------------------------

year |

2019 | -.0092739 .0044711 -2.07 0.038 -.0180371 -.0005107

2021 | -.0192061 .0044131 -4.35 0.000 -.0278557 -.0105565

2023 | -.0335187 .0045351 -7.39 0.000 -.0424072 -.0246301

|

schoollevel |

upper secondary | .0024305 .005645 0.43 0.667 -.0086335 .0134946

vocational | .043577 .0053114 8.20 0.000 .0331669 .053987

|

year#schoollevel |

2019#upper secondary | -.0058832 .0075898 -0.78 0.438 -.020759 .0089926

2019#vocational | -.0165308 .0076694 -2.16 0.031 -.0315625 -.0014992

2021#upper secondary | -.0125373 .0075385 -1.66 0.096 -.0273124 .0022378

2021#vocational | -.0175796 .0077665 -2.26 0.024 -.0328017 -.0023576

2023#upper secondary | -.0023857 .0076616 -0.31 0.756 -.0174022 .0126308

2023#vocational | -.0266383 .0083437 -3.19 0.001 -.0429916 -.0102851

|

gender |

girl | .0007389 .0043607 0.17 0.865 -.007808 .0092857

|

year#gender |

2019#girl | -.0066821 .0059113 -1.13 0.258 -.0182681 .0049039

2021#girl | -.0645066 .0059314 -10.88 0.000 -.0761319 -.0528812

2023#girl | -.0674036 .0061224 -11.01 0.000 -.0794034 -.0554039

|

schoollevel#gender |

upper secondary#girl | .0233529 .0071644 3.26 0.001 .0093109 .037395

vocational#girl | -.0447968 .0076134 -5.88 0.000 -.0597188 -.0298747

|

year#schoollevel#gender |

2019#upper secondary#girl | .0015159 .0096599 0.16 0.875 -.0174173 .020449

2019#vocational#girl | .0061794 .0110798 0.56 0.577 -.0155367 .0278955

2021#upper secondary#girl | .0304367 .0096643 3.15 0.002 .011495 .0493783

2021#vocational#girl | -.0025951 .0115213 -0.23 0.822 -.0251765 .0199863

2023#upper secondary#girl | .0332789 .0098876 3.37 0.001 .0138996 .0526582

2023#vocational#girl | .028214 .0121906 2.31 0.021 .0043208 .0521073

|

urbanrural |

semi-urban | -.0055804 .0069993 -0.80 0.425 -.0192989 .0081381

rural | -.0150834 .0075834 -1.99 0.047 -.0299465 -.0002203

|

year#urbanrural |

2019#semi-urban | .0152143 .0094632 1.61 0.108 -.0033333 .0337618

2019#rural | .0106153 .010504 1.01 0.312 -.0099722 .0312029

2021#semi-urban | .0094995 .0094766 1.00 0.316 -.0090742 .0280732

2021#rural | .0054376 .0104212 0.52 0.602 -.0149876 .0258627

2023#semi-urban | .008405 .0097347 0.86 0.388 -.0106747 .0274847

2023#rural | .0108313 .0106803 1.01 0.311 -.0101016 .0317643

|

schoollevel#urbanrural |

upper secondary#semi-urban | -.0052911 .0128072 -0.41 0.680 -.0303927 .0198105

upper secondary#rural | -.0087837 .0149078 -0.59 0.556 -.0380025 .0204351

vocational#semi-urban | .0203923 .0115297 1.77 0.077 -.0022054 .0429901

vocational#rural | .0071278 .0191753 0.37 0.710 -.030455 .0447107

|

year#schoollevel#urbanrural |

2019#upper secondary#semi-urban | -.0195751 .0176095 -1.11 0.266 -.0540891 .0149388

2019#upper secondary#rural | -.0124454 .020711 -0.60 0.548 -.0530382 .0281473

2019#vocational#semi-urban | -.0335753 .0169272 -1.98 0.047 -.0667521 -.0003986

2019#vocational#rural | .0194473 .0268382 0.72 0.469 -.0331546 .0720493

2021#upper secondary#semi-urban | -.0006492 .0174289 -0.04 0.970 -.0348091 .0335108

2021#upper secondary#rural | -.0166159 .0207938 -0.80 0.424 -.0573709 .0241391

2021#vocational#semi-urban | -.010787 .0169416 -0.64 0.524 -.043992 .0224179

2021#vocational#rural | .0018225 .0280097 0.07 0.948 -.0530756 .0567205

2023#upper secondary#semi-urban | -.0196124 .0182327 -1.08 0.282 -.0553479 .016123

2023#upper secondary#rural | -.0211829 .0213399 -0.99 0.321 -.0630084 .0206426

2023#vocational#semi-urban | .0024563 .0174339 0.14 0.888 -.0317135 .036626

2023#vocational#rural | .0153855 .0284674 0.54 0.589 -.0404096 .0711806

|

gender#urbanrural |

girl#semi-urban | -.0111188 .009263 -1.20 0.230 -.029274 .0070364

girl#rural | -.0147294 .0100907 -1.46 0.144 -.0345068 .005048

|

year#gender#urbanrural |

2019#girl#semi-urban | -.0170195 .0126443 -1.35 0.178 -.0418019 .0077629

2019#girl#rural | -.0056665 .0140697 -0.40 0.687 -.0332426 .0219096

2021#girl#semi-urban | .005492 .0127441 0.43 0.667 -.019486 .03047

2021#girl#rural | .0194028 .0140902 1.38 0.168 -.0082135 .047019

2023#girl#semi-urban | -.01278 .013296 -0.96 0.336 -.0388396 .0132796

2023#girl#rural | -.014605 .0147356 -0.99 0.322 -.0434862 .0142761

|

schoollevel#gender#urbanrural |

upper secondary#girl#semi-urban | .0102766 .0162668 0.63 0.528 -.0216057 .0421589

upper secondary#girl#rural | -.0281686 .0190729 -1.48 0.140 -.0655507 .0092135

vocational#girl#semi-urban | -.0393606 .0182201 -2.16 0.031 -.0750713 -.0036498

vocational#girl#rural | -.0281658 .0280457 -1.00 0.315 -.0831343 .0268027

|

year#schoollevel#gender#urbanrural |

2019#upper secondary#girl#semi-urban | .0067874 .0224217 0.30 0.762 -.0371583 .0507332

2019#upper secondary#girl#rural | .0231346 .0264157 0.88 0.381 -.0286392 .0749083

2019#vocational#girl#semi-urban | .0405118 .0266209 1.52 0.128 -.0116642 .0926878

2019#vocational#girl#rural | -.009555 .0397328 -0.24 0.810 -.08743 .0683199

2021#upper secondary#girl#semi-urban | -.0444 .0225002 -1.97 0.048 -.0884996 -.0003004

2021#upper secondary#girl#rural | .0258694 .0265747 0.97 0.330 -.0262161 .0779548

2021#vocational#girl#semi-urban | .0166786 .0271715 0.61 0.539 -.0365765 .0699338

2021#vocational#girl#rural | -.0460329 .0427344 -1.08 0.281 -.1297907 .037725

2023#upper secondary#girl#semi-urban | .0063464 .0235587 0.27 0.788 -.0398278 .0525205

2023#upper secondary#girl#rural | .0156728 .027918 0.56 0.575 -.0390455 .0703911

2023#vocational#girl#semi-urban | .0381513 .0279885 1.36 0.173 -.016705 .0930077

2023#vocational#girl#rural | -.0334433 .0447233 -0.75 0.455 -.1210993 .0542127

|

1.degree | .0318605 .0013145 24.24 0.000 .0292841 .0344368

|

immigrant |

One foreign-born parent | -.0388331 .0025276 -15.36 0.000 -.0437871 -.0338791

Born in Finland, foreign-born parents | -.0668858 .0049262 -13.58 0.000 -.076541 -.0572306

Student and parents born abroad | -.1794417 .0046765 -38.37 0.000 -.1886075 -.170276

|

_cons | .7924278 .0034029 232.87 0.000 .7857583 .7990974

--------------------------------------------------------------------------------------------------------

. testparm year#schoollevel#gender#urbanrural

( 1) [closefriend3]2019.year#20.schoollevel#2.gender#2.urbanrural = 0

( 2) [closefriend3]2019.year#20.schoollevel#2.gender#3.urbanrural = 0

( 3) [closefriend3]2019.year#30.schoollevel#2.gender#2.urbanrural = 0

( 4) [closefriend3]2019.year#30.schoollevel#2.gender#3.urbanrural = 0

( 5) [closefriend3]2021.year#20.schoollevel#2.gender#2.urbanrural = 0

( 6) [closefriend3]2021.year#20.schoollevel#2.gender#3.urbanrural = 0

( 7) [closefriend3]2021.year#30.schoollevel#2.gender#2.urbanrural = 0

( 8) [closefriend3]2021.year#30.schoollevel#2.gender#3.urbanrural = 0

( 9) [closefriend3]2023.year#20.schoollevel#2.gender#2.urbanrural = 0

(10) [closefriend3]2023.year#20.schoollevel#2.gender#3.urbanrural = 0

(11) [closefriend3]2023.year#30.schoollevel#2.gender#2.urbanrural = 0

(12) [closefriend3]2023.year#30.schoollevel#2.gender#3.urbanrural = 0

chi2( 12) = 14.21

Prob > chi2 = 0.2877

.

. poisson closefriend3 i.year##i.schoollevel##i.gender##i.immigrant i.degree i.urbanrural, vce(robust)

Iteration 0: Log pseudolikelihood = -872584.26

Iteration 1: Log pseudolikelihood = -872584.26

Poisson regression Number of obs = 556,754

Wald chi2(98) = 6329.35

Prob > chi2 = 0.0000

Log pseudolikelihood = -872584.26 Pseudo R2 = 0.0020

------------------------------------------------------------------------------------------------------------------------

| Robust

closefriend3 | Coefficient std. err. z P>|z| [95% conf. interval]

-------------------------------------------------------+----------------------------------------------------------------

year |

2019 | -.0052761 .0037961 -1.39 0.165 -.0127164 .0021642

2021 | -.0140808 .0037589 -3.75 0.000 -.0214481 -.0067134

2023 | -.0283089 .0038731 -7.31 0.000 -.0359001 -.0207177

|

schoollevel |

upper secondary | -.0031309 .0049427 -0.63 0.526 -.0128184 .0065567

vocational | .0443599 .0046687 9.50 0.000 .0352094 .0535104

|

year#schoollevel |

2019#upper secondary | -.0125964 .0067207 -1.87 0.061 -.0257688 .000576

2019#vocational | -.022415 .006789 -3.30 0.001 -.0357212 -.0091088

2021#upper secondary | -.0195854 .0066851 -2.93 0.003 -.0326879 -.0064829

2021#vocational | -.0213038 .0068627 -3.10 0.002 -.0347546 -.0078531

2023#upper secondary | -.0097476 .0068419 -1.42 0.154 -.0231575 .0036623

2023#vocational | -.0265447 .0073061 -3.63 0.000 -.0408644 -.012225

|

gender |

girl | -.0073136 .0037032 -1.97 0.048 -.0145716 -.0000555

|

year#gender |

2019#girl | -.0098376 .0050567 -1.95 0.052 -.0197485 .0000733

2021#girl | -.0631456 .0050827 -12.42 0.000 -.0731074 -.0531837

2023#girl | -.0751873 .005287 -14.22 0.000 -.0855497 -.064825

|

schoollevel#gender |

upper secondary#girl | .0246248 .0062849 3.92 0.000 .0123065 .0369431

vocational#girl | -.0476441 .0068624 -6.94 0.000 -.0610941 -.0341941

|

year#schoollevel#gender |

2019#upper secondary#girl | .0062308 .0085696 0.73 0.467 -.0105653 .023027

2019#vocational#girl | .0098833 .0100425 0.98 0.325 -.0097996 .0295662

2021#upper secondary#girl | .0326168 .0085883 3.80 0.000 .015784 .0494497

2021#vocational#girl | -.0071198 .0104441 -0.68 0.495 -.0275899 .0133503

2023#upper secondary#girl | .0393303 .0088713 4.43 0.000 .0219429 .0567176

2023#vocational#girl | .0289158 .0110384 2.62 0.009 .0072809 .0505507

|

immigrant |

One foreign-born parent | -.0377223 .0108488 -3.48 0.001 -.0589856 -.0164589

Born in Finland, foreign-born parents | -.0705434 .022804 -3.09 0.002 -.1152384 -.0258485

Student and parents born abroad | -.2585342 .020516 -12.60 0.000 -.2987448 -.2183235

|

year#immigrant |

2019#One foreign-born parent | -.0127178 .0149581 -0.85 0.395 -.0420352 .0165996

2019#Born in Finland, foreign-born parents | .0136332 .0299493 0.46 0.649 -.0450664 .0723327

2019#Student and parents born abroad | .0179542 .0275977 0.65 0.515 -.0361363 .0720447

2021#One foreign-born parent | -.0374381 .0149846 -2.50 0.012 -.0668074 -.0080689

2021#Born in Finland, foreign-born parents | .0039766 .0288814 0.14 0.890 -.0526299 .060583

2021#Student and parents born abroad | -.0082656 .0271625 -0.30 0.761 -.061503 .0449719

2023#One foreign-born parent | -.0422708 .0151735 -2.79 0.005 -.0720103 -.0125313

2023#Born in Finland, foreign-born parents | -.0113569 .0296082 -0.38 0.701 -.0693879 .046674

2023#Student and parents born abroad | .0356133 .0263497 1.35 0.177 -.016031 .0872577

|

schoollevel#immigrant |

upper secondary#One foreign-born parent | .0002838 .0199072 0.01 0.989 -.0387337 .0393013

upper secondary#Born in Finland, foreign-born parents | -.0111453 .0423457 -0.26 0.792 -.0941414 .0718508

upper secondary#Student and parents born abroad | .1128836 .0413607 2.73 0.006 .0318181 .193949

vocational#One foreign-born parent | -.0007137 .0204587 -0.03 0.972 -.040812 .0393847

vocational#Born in Finland, foreign-born parents | .0767283 .0384732 1.99 0.046 .0013221 .1521344

vocational#Student and parents born abroad | .0255254 .0378358 0.67 0.500 -.0486313 .0996821

|

year#schoollevel#immigrant |

2019#upper secondary#One foreign-born parent | .0348534 .0264023 1.32 0.187 -.0168942 .0866009

2019 #|

upper secondary #|

Born in Finland, foreign-born parents | .0036191 .0543279 0.07 0.947 -.1028616 .1100999

2019#upper secondary#Student and parents born abroad | -.0240428 .0534746 -0.45 0.653 -.1288511 .0807654

2019#vocational#One foreign-born parent | .018215 .0289823 0.63 0.530 -.0385893 .0750194

2019#vocational#Born in Finland, foreign-born parents | -.0607611 .056406 -1.08 0.281 -.1713147 .0497926

2019#vocational#Student and parents born abroad | -.0007614 .0530293 -0.01 0.989 -.1046968 .1031741

2021#upper secondary#One foreign-born parent | .0590544 .0263374 2.24 0.025 .007434 .1106749

2021 #|

upper secondary #|

Born in Finland, foreign-born parents | .0250708 .0525667 0.48 0.633 -.0779581 .1280997

2021#upper secondary#Student and parents born abroad | .0060401 .0522981 0.12 0.908 -.0964623 .1085425

2021#vocational#One foreign-born parent | .01731 .0300293 0.58 0.564 -.0415464 .0761663

2021#vocational#Born in Finland, foreign-born parents | -.1132922 .0596611 -1.90 0.058 -.2302258 .0036414

2021#vocational#Student and parents born abroad | .0669269 .0521874 1.28 0.200 -.0353585 .1692123

2023#upper secondary#One foreign-born parent | .0212094 .0269531 0.79 0.431 -.0316178 .0740365

2023 #|

upper secondary #|

Born in Finland, foreign-born parents | .030127 .0534681 0.56 0.573 -.0746687 .1349226

2023#upper secondary#Student and parents born abroad | -.025478 .0499192 -0.51 0.610 -.1233179 .0723619

2023#vocational#One foreign-born parent | .0432948 .0303439 1.43 0.154 -.0161781 .1027678

2023#vocational#Born in Finland, foreign-born parents | -.0748366 .0584801 -1.28 0.201 -.1894555 .0397822

2023#vocational#Student and parents born abroad | .0018997 .05164 0.04 0.971 -.0993129 .1031123

|

gender#immigrant |

girl#One foreign-born parent | -.0038112 .0143188 -0.27 0.790 -.0318755 .0242531

girl#Born in Finland, foreign-born parents | .012092 .029392 0.41 0.681 -.0455153 .0696994

girl#Student and parents born abroad | .1188877 .0278063 4.28 0.000 .0643884 .1733871

|

year#gender#immigrant |

2019#girl#One foreign-born parent | .025505 .019515 1.31 0.191 -.0127437 .0637538

2019#girl#Born in Finland, foreign-born parents | -.0351136 .039383 -0.89 0.373 -.1123028 .0420757

2019#girl#Student and parents born abroad | -.0553716 .0378817 -1.46 0.144 -.1296184 .0188751

2021#girl#One foreign-born parent | .0464202 .019708 2.36 0.019 .0077932 .0850473

2021#girl#Born in Finland, foreign-born parents | -.0103947 .0378478 -0.27 0.784 -.0845749 .0637855

2021#girl#Student and parents born abroad | -.025038 .0372008 -0.67 0.501 -.0979502 .0478742

2023#girl#One foreign-born parent | .0521668 .0202037 2.58 0.010 .0125683 .0917653

2023#girl#Born in Finland, foreign-born parents | .0199674 .038928 0.51 0.608 -.0563301 .0962649

2023#girl#Student and parents born abroad | -.0265261 .0361924 -0.73 0.464 -.0974619 .0444096

|

schoollevel#gender#immigrant |

upper secondary#girl#One foreign-born parent | .0128979 .0253584 0.51 0.611 -.0368036 .0625995

upper secondary #|

girl #|

Born in Finland, foreign-born parents | .0175222 .05289 0.33 0.740 -.0861403 .1211847

upper secondary#girl#Student and parents born abroad | -.0700504 .0517333 -1.35 0.176 -.1714458 .0313451

vocational#girl#One foreign-born parent | .0363607 .0284389 1.28 0.201 -.0193785 .0920999

vocational#girl#Born in Finland, foreign-born parents | -.1572084 .0594611 -2.64 0.008 -.2737499 -.0406668

vocational#girl#Student and parents born abroad | -.0912504 .054727 -1.67 0.095 -.1985133 .0160125

|

year#schoollevel#gender#immigrant |

2019#upper secondary#girl#One foreign-born parent | -.0386863 .0335066 -1.15 0.248 -.1043579 .0269854

2019 #|

upper secondary #|

girl #|

Born in Finland, foreign-born parents | -.0048487 .068957 -0.07 0.944 -.1400019 .1303044

2019 #|

upper secondary #|

girl #|

Student and parents born abroad | .0733077 .067401 1.09 0.277 -.0587958 .2054112

2019#vocational#girl#One foreign-born parent | -.0553716 .0410165 -1.35 0.177 -.1357624 .0250192

2019 #|

vocational #|

girl #|

Born in Finland, foreign-born parents | .0919779 .0899397 1.02 0.306 -.0843008 .2682565

2019#vocational#girl#Student and parents born abroad | .1795592 .0749833 2.39 0.017 .0325945 .3265238

2021#upper secondary#girl#One foreign-born parent | -.0804792 .0336249 -2.39 0.017 -.1463828 -.0145755

2021 #|

upper secondary #|

girl #|

Born in Finland, foreign-born parents | -.073222 .0673692 -1.09 0.277 -.2052633 .0588193

2021 #|

upper secondary #|

girl #|

Student and parents born abroad | .003181 .0662417 0.05 0.962 -.1266504 .1330123

2021#vocational#girl#One foreign-born parent | -.0327057 .0425744 -0.77 0.442 -.1161499 .0507386

2021 #|

vocational #|

girl #|

Born in Finland, foreign-born parents | .1462298 .0947177 1.54 0.123 -.0394136 .3318732

2021#vocational#girl#Student and parents born abroad | .0688583 .0765088 0.90 0.368 -.0810963 .2188128

2023#upper secondary#girl#One foreign-born parent | -.0127403 .0344274 -0.37 0.711 -.0802167 .0547362

2023 #|

upper secondary #|

girl #|

Born in Finland, foreign-born parents | -.0467651 .0676726 -0.69 0.490 -.1794009 .0858707

2023 #|

upper secondary #|

girl #|

Student and parents born abroad | -.0105714 .0637505 -0.17 0.868 -.13552 .1143773

2023#vocational#girl#One foreign-born parent | -.0746954 .0434942 -1.72 0.086 -.1599425 .0105516

2023 #|

vocational #|

girl #|

Born in Finland, foreign-born parents | .2586207 .0857484 3.02 0.003 .090557 .4266844

2023#vocational#girl#Student and parents born abroad | .1003703 .0753566 1.33 0.183 -.047326 .2480665

|

1.degree | .0318733 .0013139 24.26 0.000 .0292981 .0344484

|

urbanrural |

semi-urban | -.0092021 .0017016 -5.41 0.000 -.0125372 -.005867

rural | -.0240535 .002047 -11.75 0.000 -.0280656 -.0200414

|

_cons | .7972572 .0029355 271.59 0.000 .7915038 .8030107

------------------------------------------------------------------------------------------------------------------------

. testparm year#schoollevel#gender#immigrant

( 1) [closefriend3]2019.year#20.schoollevel#2.gender#2.immigrant = 0

( 2) [closefriend3]2019.year#20.schoollevel#2.gender#3.immigrant = 0

( 3) [closefriend3]2019.year#20.schoollevel#2.gender#4.immigrant = 0

( 4) [closefriend3]2019.year#30.schoollevel#2.gender#2.immigrant = 0

( 5) [closefriend3]2019.year#30.schoollevel#2.gender#3.immigrant = 0

( 6) [closefriend3]2019.year#30.schoollevel#2.gender#4.immigrant = 0

( 7) [closefriend3]2021.year#20.schoollevel#2.gender#2.immigrant = 0

( 8) [closefriend3]2021.year#20.schoollevel#2.gender#3.immigrant = 0

( 9) [closefriend3]2021.year#20.schoollevel#2.gender#4.immigrant = 0

(10) [closefriend3]2021.year#30.schoollevel#2.gender#2.immigrant = 0

(11) [closefriend3]2021.year#30.schoollevel#2.gender#3.immigrant = 0

(12) [closefriend3]2021.year#30.schoollevel#2.gender#4.immigrant = 0

(13) [closefriend3]2023.year#20.schoollevel#2.gender#2.immigrant = 0

(14) [closefriend3]2023.year#20.schoollevel#2.gender#3.immigrant = 0

(15) [closefriend3]2023.year#20.schoollevel#2.gender#4.immigrant = 0

(16) [closefriend3]2023.year#30.schoollevel#2.gender#2.immigrant = 0

(17) [closefriend3]2023.year#30.schoollevel#2.gender#3.immigrant = 0

(18) [closefriend3]2023.year#30.schoollevel#2.gender#4.immigrant = 0

chi2( 18) = 32.02

Prob > chi2 = 0.0219

.

. poisson closefriend3 i.year##i.schoollevel##i.degree i.year##i.gender##i.degree i.urbanrural i.immigrant, vce(robust)

Iteration 0: Log pseudolikelihood = -872737.45

Iteration 1: Log pseudolikelihood = -872737.45

Poisson regression Number of obs = 556,754

Wald chi2(36) = 5874.86

Prob > chi2 = 0.0000

Log pseudolikelihood = -872737.45 Pseudo R2 = 0.0018

--------------------------------------------------------------------------------------------------------

| Robust

closefriend3 | Coefficient std. err. z P>|z| [95% conf. interval]

---------------------------------------+----------------------------------------------------------------

year |

2019 | -.003261 .0045994 -0.71 0.478 -.0122756 .0057536

2021 | -.0165293 .0046321 -3.57 0.000 -.0256079 -.0074506

2023 | -.0344643 .004847 -7.11 0.000 -.0439641 -.0249644

|

schoollevel |

upper secondary | .0065829 .0047718 1.38 0.168 -.0027696 .0159354

vocational | .0207972 .0042788 4.86 0.000 .0124109 .0291835

|

year#schoollevel |

2019#upper secondary | -.0030477 .0066655 -0.46 0.648 -.0161119 .0100165

2019#vocational | -.0089336 .0062644 -1.43 0.154 -.0212116 .0033445

2021#upper secondary | -.0078386 .0068475 -1.14 0.252 -.0212595 .0055822

2021#vocational | -.018512 .0065131 -2.84 0.004 -.0312775 -.0057465

2023#upper secondary | .0027748 .0073022 0.38 0.704 -.0115373 .0170869

2023#vocational | -.008616 .0070153 -1.23 0.219 -.0223657 .0051337

|

1.degree | .0205495 .0045729 4.49 0.000 .0115868 .0295123

|

year#degree |

2019 1 | -.0082112 .0062682 -1.31 0.190 -.0204965 .0040742

2021 1 | -.0091429 .0062642 -1.46 0.144 -.0214204 .0031347

2023 1 | -.0089475 .0064853 -1.38 0.168 -.0216586 .0037635

|

schoollevel#degree |

upper secondary#1 | .0119789 .0061012 1.96 0.050 .0000207 .0239371

vocational#1 | .012479 .006784 1.84 0.066 -.0008173 .0257753

|

year#schoollevel#degree |

2019#upper secondary#1 | -.0076073 .0083807 -0.91 0.364 -.0240332 .0088187

2019#vocational#1 | -.0211961 .0099091 -2.14 0.032 -.0406176 -.0017745

2021#upper secondary#1 | .0111376 .008516 1.31 0.191 -.0055534 .0278286

2021#vocational#1 | -.0097548 .0101789 -0.96 0.338 -.029705 .0101954

2023#upper secondary#1 | .0125665 .0089484 1.40 0.160 -.004972 .0301051

2023#vocational#1 | -.0079473 .0106308 -0.75 0.455 -.0287833 .0128888

|

gender |

girl | -.0149992 .0036785 -4.08 0.000 -.022209 -.0077894

|

year#gender |

2019#girl | -.01581 .0052422 -3.02 0.003 -.0260845 -.0055355

2021#girl | -.0549472 .0053641 -10.24 0.000 -.0654607 -.0444337

2023#girl | -.0582684 .0056907 -10.24 0.000 -.069422 -.0471148

|

gender#degree |

girl#1 | .0157787 .0050942 3.10 0.002 .0057943 .0257631

|

year#gender#degree |

2019#girl#1 | .0195931 .0070807 2.77 0.006 .0057151 .033471

2021#girl#1 | .0070052 .0071666 0.98 0.328 -.007041 .0210514

2023#girl#1 | .008571 .007482 1.15 0.252 -.0060934 .0232354

|

urbanrural |

semi-urban | -.0088844 .0017023 -5.22 0.000 -.0122209 -.0055479

rural | -.023969 .0020493 -11.70 0.000 -.0279856 -.0199523

|

immigrant |

One foreign-born parent | -.0388685 .0025282 -15.37 0.000 -.0438236 -.0339134

Born in Finland, foreign-born parents | -.0663278 .0049287 -13.46 0.000 -.0759878 -.0566678

Student and parents born abroad | -.1793291 .0046774 -38.34 0.000 -.1884966 -.1701616

|

_cons | .8019352 .0033462 239.66 0.000 .7953769 .8084936

--------------------------------------------------------------------------------------------------------

. testparm year#gender#degree

( 1) [closefriend3]2019.year#2.gender#1.degree = 0

( 2) [closefriend3]2021.year#2.gender#1.degree = 0

( 3) [closefriend3]2023.year#2.gender#1.degree = 0

chi2( 3) = 7.92

Prob > chi2 = 0.0476

. testparm year#schoollevel#degree

( 1) [closefriend3]2019.year#20.schoollevel#1.degree = 0

( 2) [closefriend3]2019.year#30.schoollevel#1.degree = 0

( 3) [closefriend3]2021.year#20.schoollevel#1.degree = 0

( 4) [closefriend3]2021.year#30.schoollevel#1.degree = 0

( 5) [closefriend3]2023.year#20.schoollevel#1.degree = 0

( 6) [closefriend3]2023.year#30.schoollevel#1.degree = 0

chi2( 6) = 11.83

Prob > chi2 = 0.0660

.

. poisson closefriend3 i.year##i.schoollevel##i.urbanrural i.year##i.gender#i.urbanrural i.degree i.immigrant, vce(robust)

Iteration 0: Log pseudolikelihood = -872737.32

Iteration 1: Log pseudolikelihood = -872737.32

Poisson regression Number of obs = 556,754

Wald chi2(51) = 5838.11

Prob > chi2 = 0.0000

Log pseudolikelihood = -872737.32 Pseudo R2 = 0.0018

--------------------------------------------------------------------------------------------------------

| Robust

closefriend3 | Coefficient std. err. z P>|z| [95% conf. interval]

---------------------------------------+----------------------------------------------------------------

year |

2019 | -.0117378 .0037787 -3.11 0.002 -.0191438 -.0043318

2021 | -.0261747 .0037595 -6.96 0.000 -.0335432 -.0188063

2023 | -.0437152 .0038737 -11.29 0.000 -.0513075 -.0361228

|

schoollevel |

upper secondary | .0164965 .003498 4.72 0.000 .0096405 .0233524

vocational | .0242665 .0038128 6.36 0.000 .0167934 .0317395

|

year#schoollevel |

2019#upper secondary | -.0051881 .004713 -1.10 0.271 -.0144254 .0040492

2019#vocational | -.0134186 .0055328 -2.43 0.015 -.0242628 -.0025744

2021#upper secondary | .0045482 .0047262 0.96 0.336 -.004715 .0138114

2021#vocational | -.0170329 .0057223 -2.98 0.003 -.0282484 -.0058175

2023#upper secondary | .0154831 .0048477 3.19 0.001 .0059819 .0249843

2023#vocational | -.0134577 .0060782 -2.21 0.027 -.0253707 -.0015447

|

urbanrural |

semi-urban | -.0042034 .0060149 -0.70 0.485 -.0159924 .0075856

rural | -.0133642 .0067517 -1.98 0.048 -.0265974 -.000131

|

year#urbanrural |

2019#semi-urban | .0115318 .0082218 1.40 0.161 -.0045826 .0276461

2019#rural | .0099004 .0093761 1.06 0.291 -.0084764 .0282772

2021#semi-urban | .0154361 .0082408 1.87 0.061 -.0007155 .0315877

2021#rural | .006422 .0093575 0.69 0.493 -.0119185 .0247624

2023#semi-urban | .0075134 .0085242 0.88 0.378 -.0091937 .0242206

2023#rural | .0145802 .0096371 1.51 0.130 -.0043081 .0334686

|

schoollevel#urbanrural |

upper secondary#semi-urban | .0007034 .007936 0.09 0.929 -.0148509 .0162577

upper secondary#rural | -.0251055 .0093347 -2.69 0.007 -.0434011 -.0068099

vocational#semi-urban | .0102632 .0088464 1.16 0.246 -.0070755 .0276019

vocational#rural | -.005892 .0140307 -0.42 0.675 -.0333917 .0216078

|

year#schoollevel#urbanrural |

2019#upper secondary#semi-urban | -.0162851 .0109482 -1.49 0.137 -.0377432 .0051729

2019#upper secondary#rural | .0011144 .012899 0.09 0.931 -.0241672 .026396

2019#vocational#semi-urban | -.0189255 .0129863 -1.46 0.145 -.0443781 .0065271

2019#vocational#rural | .0142692 .019826 0.72 0.472 -.0245891 .0531274

2021#upper secondary#semi-urban | -.0264813 .0110445 -2.40 0.016 -.0481281 -.0048345

2021#upper secondary#rural | -.0009942 .0129822 -0.08 0.939 -.0264388 .0244505

2021#vocational#semi-urban | -.0067985 .0131512 -0.52 0.605 -.0325744 .0189774

2021#vocational#rural | -.0214735 .0212367 -1.01 0.312 -.0630967 .0201496

2023#upper secondary#semi-urban | -.0169641 .0115622 -1.47 0.142 -.0396256 .0056974

2023#upper secondary#rural | -.0122779 .0137593 -0.89 0.372 -.0392456 .0146898

2023#vocational#semi-urban | .0153616 .0135387 1.13 0.257 -.0111737 .0418969

2023#vocational#rural | .0034452 .0219121 0.16 0.875 -.0395017 .046392

|

gender#urbanrural |

girl#urban | -.0024556 .0030213 -0.81 0.416 -.0083772 .003466

girl#semi-urban | -.0163441 .006099 -2.68 0.007 -.0282979 -.0043902

girl#rural | -.0207845 .0074571 -2.79 0.005 -.0354001 -.006169

|

year#gender#urbanrural |

2019#girl#urban | -.0019624 .0041614 -0.47 0.637 -.0101186 .0061938

2019#girl#semi-urban | -.0117676 .0084983 -1.38 0.166 -.0284239 .0048888

2019#girl#rural | -.006079 .0104345 -0.58 0.560 -.0265303 .0143722

2021#girl#urban | -.0509106 .0041927 -12.14 0.000 -.0591281 -.0426932

2021#girl#semi-urban | -.0570404 .0085695 -6.66 0.000 -.0738363 -.0402446

2021#girl#rural | -.0332508 .010515 -3.16 0.002 -.0538598 -.0126417

2023#girl#urban | -.0475305 .0043279 -10.98 0.000 -.0560131 -.0390479

2023#girl#semi-urban | -.0580629 .0089592 -6.48 0.000 -.0756227 -.0405031

2023#girl#rural | -.0690056 .0110508 -6.24 0.000 -.0906647 -.0473464

|

1.degree | .0315753 .0013149 24.01 0.000 .0289981 .0341525

|

immigrant |

One foreign-born parent | -.0389963 .002528 -15.43 0.000 -.0439511 -.0340414

Born in Finland, foreign-born parents | -.0669654 .0049276 -13.59 0.000 -.0766234 -.0573074

Student and parents born abroad | -.1791488 .0046758 -38.31 0.000 -.1883132 -.1699843

|

_cons | .7942705 .0028887 274.95 0.000 .7886086 .7999323

--------------------------------------------------------------------------------------------------------

. testparm year#gender#urbanrural

( 1) [closefriend3]2019.year#2.gender#1b.urbanrural = 0

( 2) [closefriend3]2019.year#2.gender#2.urbanrural = 0

( 3) [closefriend3]2019.year#2.gender#3.urbanrural = 0

( 4) [closefriend3]2021.year#2.gender#1b.urbanrural = 0

( 5) [closefriend3]2021.year#2.gender#2.urbanrural = 0

( 6) [closefriend3]2021.year#2.gender#3.urbanrural = 0

( 7) [closefriend3]2023.year#2.gender#1b.urbanrural = 0

( 8) [closefriend3]2023.year#2.gender#2.urbanrural = 0

( 9) [closefriend3]2023.year#2.gender#3.urbanrural = 0

chi2( 9) = 385.93

Prob > chi2 = 0.0000

. testparm year#schoollevel#urbanrural

( 1) [closefriend3]2019.year#20.schoollevel#2.urbanrural = 0

( 2) [closefriend3]2019.year#20.schoollevel#3.urbanrural = 0

( 3) [closefriend3]2019.year#30.schoollevel#2.urbanrural = 0

( 4) [closefriend3]2019.year#30.schoollevel#3.urbanrural = 0

( 5) [closefriend3]2021.year#20.schoollevel#2.urbanrural = 0

( 6) [closefriend3]2021.year#20.schoollevel#3.urbanrural = 0

( 7) [closefriend3]2021.year#30.schoollevel#2.urbanrural = 0

( 8) [closefriend3]2021.year#30.schoollevel#3.urbanrural = 0

( 9) [closefriend3]2023.year#20.schoollevel#2.urbanrural = 0

(10) [closefriend3]2023.year#20.schoollevel#3.urbanrural = 0

(11) [closefriend3]2023.year#30.schoollevel#2.urbanrural = 0

(12) [closefriend3]2023.year#30.schoollevel#3.urbanrural = 0

chi2( 12) = 17.04

Prob > chi2 = 0.1482

.

. poisson closefriend3 i.year##i.schoollevel##i.immigrant i.year##i.gender##i.immigrant i.degree i.urbanrural , vce(robust)

Iteration 0: Log pseudolikelihood = -872711.89

Iteration 1: Log pseudolikelihood = -872711.89

Poisson regression Number of obs = 556,754

Wald chi2(66) = 5795.72

Prob > chi2 = 0.0000

Log pseudolikelihood = -872711.89 Pseudo R2 = 0.0019

------------------------------------------------------------------------------------------------------------------------

| Robust

closefriend3 | Coefficient std. err. z P>|z| [95% conf. interval]

-------------------------------------------------------+----------------------------------------------------------------

year |

2019 | -.008433 .0032587 -2.59 0.010 -.01482 -.0020461

2021 | -.0204698 .0032479 -6.30 0.000 -.0268356 -.0141041

2023 | -.0384614 .0033607 -11.44 0.000 -.0450482 -.0318747

|

schoollevel |

upper secondary | .0116349 .0030752 3.78 0.000 .0056077 .0176622

vocational | .0247108 .00343 7.20 0.000 .0179882 .0314334

|

year#schoollevel |

2019#upper secondary | -.009233 .0041839 -2.21 0.027 -.0174334 -.0010327

2019#vocational | -.0178711 .0049966 -3.58 0.000 -.0276643 -.0080779

2021#upper secondary | -.001352 .0042038 -0.32 0.748 -.0095912 .0068873

2021#vocational | -.022681 .0051588 -4.40 0.000 -.0327921 -.01257

2023#upper secondary | .011333 .0043562 2.60 0.009 .002795 .019871

2023#vocational | -.0131351 .0054674 -2.40 0.016 -.023851 -.0024193

|

immigrant |

One foreign-born parent | -.0432472 .0093998 -4.60 0.000 -.0616705 -.0248239

Born in Finland, foreign-born parents | -.0585781 .0194027 -3.02 0.003 -.0966068 -.0205494

Student and parents born abroad | -.2441025 .0179117 -13.63 0.000 -.2792088 -.2089962

|

year#immigrant |

2019#One foreign-born parent | -.0020402 .0128579 -0.16 0.874 -.0272412 .0231608

2019#Born in Finland, foreign-born parents | .0022351 .0258192 0.09 0.931 -.0483696 .0528398

2019#Student and parents born abroad | -.0021791 .0242108 -0.09 0.928 -.0496314 .0452732

2021#One foreign-born parent | -.0217957 .0129182 -1.69 0.092 -.0471149 .0035235

2021#Born in Finland, foreign-born parents | -.0002478 .0249893 -0.01 0.992 -.0492258 .0487302

2021#Student and parents born abroad | -.0118136 .0237717 -0.50 0.619 -.0584052 .0347781

2023#One foreign-born parent | -.0350037 .0131726 -2.66 0.008 -.0608215 -.0091858

2023#Born in Finland, foreign-born parents | -.0249943 .0255319 -0.98 0.328 -.0750358 .0250472

2023#Student and parents born abroad | .0327866 .0230482 1.42 0.155 -.0123869 .0779602

|

schoollevel#immigrant |

upper secondary#One foreign-born parent | .0070542 .0123795 0.57 0.569 -.0172091 .0313176

upper secondary#Born in Finland, foreign-born parents | .0005885 .0255338 0.02 0.982 -.0494568 .0506338

upper secondary#Student and parents born abroad | .0767246 .0253692 3.02 0.002 .027002 .1264472

vocational#One foreign-born parent | .0142931 .0142504 1.00 0.316 -.0136372 .0422233

vocational#Born in Finland, foreign-born parents | .0042568 .0293817 0.14 0.885 -.0533302 .0618438

vocational#Student and parents born abroad | -.011223 .0276799 -0.41 0.685 -.0654746 .0430286

|

year#schoollevel#immigrant |

2019#upper secondary#One foreign-born parent | .0132443 .0163195 0.81 0.417 -.0187414 .04523

2019 #|

upper secondary #|

Born in Finland, foreign-born parents | -.0008577 .033543 -0.03 0.980 -.0666007 .0648854

2019#upper secondary#Student and parents born abroad | .0121279 .0329855 0.37 0.713 -.0525225 .0767782

2019#vocational#One foreign-born parent | -.0065211 .0205257 -0.32 0.751 -.0467508 .0337086

2019#vocational#Born in Finland, foreign-born parents | -.0100804 .0438476 -0.23 0.818 -.0960201 .0758593

2019#vocational#Student and parents born abroad | .0725513 .0380762 1.91 0.057 -.0020766 .1471792

2021#upper secondary#One foreign-born parent | .0136291 .0164181 0.83 0.406 -.0185498 .045808

2021 #|

upper secondary #|

Born in Finland, foreign-born parents | -.0185638 .0328871 -0.56 0.572 -.0830214 .0458938

2021#upper secondary#Student and parents born abroad | .0054771 .0325585 0.17 0.866 -.0583363 .0692905

2021#vocational#One foreign-born parent | -.0005636 .0212846 -0.03 0.979 -.0422806 .0411535

2021#vocational#Born in Finland, foreign-born parents | -.0462999 .046296 -1.00 0.317 -.1370385 .0444386

2021#vocational#Student and parents born abroad | .0928974 .0384624 2.42 0.016 .0175125 .1682823

2023#upper secondary#One foreign-born parent | .0158825 .0168119 0.94 0.345 -.0170683 .0488332

2023 #|

upper secondary #|

Born in Finland, foreign-born parents | .0020569 .0328652 0.06 0.950 -.0623577 .0664716

2023#upper secondary#Student and parents born abroad | -.0336704 .0314377 -1.07 0.284 -.0952872 .0279463

2023#vocational#One foreign-born parent | .0100247 .0217314 0.46 0.645 -.0325681 .0526174

2023#vocational#Born in Finland, foreign-born parents | .0426662 .0427476 1.00 0.318 -.0411175 .12645

2023#vocational#Student and parents born abroad | .0408467 .038037 1.07 0.283 -.0337044 .1153978

|

gender |

girl | -.0098591 .002652 -3.72 0.000 -.0150568 -.0046613

|

year#gender |

2019#girl | -.0037351 .0036765 -1.02 0.310 -.0109409 .0034707

2021#girl | -.0506115 .0037079 -13.65 0.000 -.0578788 -.0433442

2023#girl | -.0551336 .0038572 -14.29 0.000 -.0626935 -.0475737

|

gender#immigrant |

girl#One foreign-born parent | .0066193 .0106568 0.62 0.535 -.0142675 .0275062

girl#Born in Finland, foreign-born parents | -.0095692 .0219382 -0.44 0.663 -.0525673 .0334289

girl#Student and parents born abroad | .0859337 .0210612 4.08 0.000 .0446545 .1272129

|

year#gender#immigrant |

2019#girl#One foreign-born parent | .0056409 .014469 0.39 0.697 -.0227178 .0339996

2019#girl#Born in Finland, foreign-born parents | -.0145376 .0296274 -0.49 0.624 -.0726061 .043531

2019#girl#Student and parents born abroad | -.0092772 .028251 -0.33 0.743 -.0646481 .0460937

2021#girl#One foreign-born parent | .0168033 .0146283 1.15 0.251 -.0118677 .0454743

2021#girl#Born in Finland, foreign-born parents | -.0037092 .0289068 -0.13 0.898 -.0603655 .052947

2021#girl#Student and parents born abroad | -.0151559 .027938 -0.54 0.587 -.0699133 .0396016

2023#girl#One foreign-born parent | .0376725 .0149733 2.52 0.012 .0083254 .0670196

2023#girl#Born in Finland, foreign-born parents | .0440258 .0291144 1.51 0.130 -.0130374 .101089

2023#girl#Student and parents born abroad | -.0173098 .0270429 -0.64 0.522 -.0703128 .0356933

|

1.degree | .0315833 .0013144 24.03 0.000 .0290072 .0341594

|

urbanrural |

semi-urban | -.008799 .001702 -5.17 0.000 -.0121348 -.0054632

rural | -.0240715 .002048 -11.75 0.000 -.0280855 -.0200575

|

_cons | .7986545 .0025477 313.48 0.000 .793661 .8036479

------------------------------------------------------------------------------------------------------------------------

. testparm year#gender#immigrant

( 1) [closefriend3]2019.year#2.gender#2.immigrant = 0

( 2) [closefriend3]2019.year#2.gender#3.immigrant = 0

( 3) [closefriend3]2019.year#2.gender#4.immigrant = 0

( 4) [closefriend3]2021.year#2.gender#2.immigrant = 0

( 5) [closefriend3]2021.year#2.gender#3.immigrant = 0

( 6) [closefriend3]2021.year#2.gender#4.immigrant = 0

( 7) [closefriend3]2023.year#2.gender#2.immigrant = 0

( 8) [closefriend3]2023.year#2.gender#3.immigrant = 0

( 9) [closefriend3]2023.year#2.gender#4.immigrant = 0

chi2( 9) = 13.16

Prob > chi2 = 0.1554

. testparm year#schoollevel#immigrant

( 1) [closefriend3]2019.year#20.schoollevel#2.immigrant = 0

( 2) [closefriend3]2019.year#20.schoollevel#3.immigrant = 0

( 3) [closefriend3]2019.year#20.schoollevel#4.immigrant = 0

( 4) [closefriend3]2019.year#30.schoollevel#2.immigrant = 0

( 5) [closefriend3]2019.year#30.schoollevel#3.immigrant = 0

( 6) [closefriend3]2019.year#30.schoollevel#4.immigrant = 0

( 7) [closefriend3]2021.year#20.schoollevel#2.immigrant = 0

( 8) [closefriend3]2021.year#20.schoollevel#3.immigrant = 0

( 9) [closefriend3]2021.year#20.schoollevel#4.immigrant = 0

(10) [closefriend3]2021.year#30.schoollevel#2.immigrant = 0

(11) [closefriend3]2021.year#30.schoollevel#3.immigrant = 0

(12) [closefriend3]2021.year#30.schoollevel#4.immigrant = 0

(13) [closefriend3]2023.year#20.schoollevel#2.immigrant = 0

(14) [closefriend3]2023.year#20.schoollevel#3.immigrant = 0

(15) [closefriend3]2023.year#20.schoollevel#4.immigrant = 0

(16) [closefriend3]2023.year#30.schoollevel#2.immigrant = 0

(17) [closefriend3]2023.year#30.schoollevel#3.immigrant = 0

(18) [closefriend3]2023.year#30.schoollevel#4.immigrant = 0

chi2( 18) = 15.57

Prob > chi2 = 0.6228

.

. poisson closefriend3 i.year##i.gender##i.degree i.year##i.gender##i.urbanrural i.year##i.schoollevel##i.gender##i.immigran

> t, vce(robust)

Iteration 0: Log pseudolikelihood = -872558.86

Iteration 1: Log pseudolikelihood = -872558.86

Poisson regression Number of obs = 556,754

Wald chi2(119) = 6468.34

Prob > chi2 = 0.0000

Log pseudolikelihood = -872558.86 Pseudo R2 = 0.0020

------------------------------------------------------------------------------------------------------------------------

| Robust

closefriend3 | Coefficient std. err. z P>|z| [95% conf. interval]

-------------------------------------------------------+----------------------------------------------------------------

year |

2019 | .0018576 .0053816 0.35 0.730 -.0086902 .0124054

2021 | -.0112439 .0053716 -2.09 0.036 -.021772 -.0007157

2023 | -.0257585 .0055737 -4.62 0.000 -.0366827 -.0148344

|

gender |

girl | -.0023432 .0051154 -0.46 0.647 -.0123692 .0076827

|

year#gender |

2019#girl | -.0225113 .0071354 -3.15 0.002 -.0364965 -.0085261

2021#girl | -.0702747 .0072157 -9.74 0.000 -.0844172 -.0561322

2023#girl | -.0781102 .0075674 -10.32 0.000 -.092942 -.0632784

|

1.degree | .0317549 .0039365 8.07 0.000 .0240395 .0394703

|

year#degree |

2019 1 | -.0145961 .0054839 -2.66 0.008 -.0253445 -.0038478

2021 1 | -.0059287 .0054917 -1.08 0.280 -.0166923 .0048349

2023 1 | -.0054882 .0056911 -0.96 0.335 -.0166425 .0056661

|

gender#degree |

girl#1 | .0040213 .0052284 0.77 0.442 -.0062261 .0142687

|

year#gender#degree |

2019#girl#1 | .0223183 .0072766 3.07 0.002 .0080565 .0365802

2021#girl#1 | .0057395 .0073607 0.78 0.436 -.0086872 .0201662

2023#girl#1 | .0066257 .0076964 0.86 0.389 -.008459 .0217105

|

urbanrural |

semi-urban | -.0020578 .0049881 -0.41 0.680 -.0118342 .0077186

rural | -.016359 .0061696 -2.65 0.008 -.0284512 -.0042668

|

year#urbanrural |

2019#semi-urban | .001119 .006966 0.16 0.872 -.0125341 .0147721

2019#rural | .0057912 .0085821 0.67 0.500 -.0110295 .0226119

2021#semi-urban | .0044579 .0069505 0.64 0.521 -.0091648 .0180806

2021#rural | -.0005562 .0085795 -0.06 0.948 -.0173716 .0162592

2023#semi-urban | .0011226 .0071883 0.16 0.876 -.0129663 .0152114

2023#rural | .0043911 .0087973 0.50 0.618 -.0128512 .0216335

|

gender#urbanrural |

girl#semi-urban | -.0146753 .006757 -2.17 0.030 -.0279187 -.0014319

girl#rural | -.0247114 .0081629 -3.03 0.002 -.0407104 -.0087123

|

year#gender#urbanrural |

2019#girl#semi-urban | -.0045245 .0094208 -0.48 0.631 -.0229889 .0139399

2019#girl#rural | .0040677 .01137 0.36 0.721 -.0182171 .0263525

2021#girl#semi-urban | -.002768 .009504 -0.29 0.771 -.0213956 .0158596

2021#girl#rural | .0244071 .011449 2.13 0.033 .0019675 .0468467

2023#girl#semi-urban | -.0039697 .0099125 -0.40 0.689 -.0233978 .0154584

2023#girl#rural | -.0096551 .0120157 -0.80 0.422 -.0332054 .0138952

|

schoollevel |

upper secondary | -.0025255 .0049963 -0.51 0.613 -.0123181 .0072672

vocational | .0453143 .0047574 9.53 0.000 .03599 .0546385

|

year#schoollevel |

2019#upper secondary | -.0097663 .0068041 -1.44 0.151 -.0231021 .0035694

2019#vocational | -.0245271 .0069144 -3.55 0.000 -.0380791 -.0109752

2021#upper secondary | -.0184248 .0067761 -2.72 0.007 -.0317057 -.005144

2021#vocational | -.02278 .0069846 -3.26 0.001 -.0364697 -.0090904

2023#upper secondary | -.0083129 .0069445 -1.20 0.231 -.0219239 .0052981

2023#vocational | -.0275765 .0074129 -3.72 0.000 -.0421056 -.0130475

|

schoollevel#gender |

upper secondary#girl | .0225884 .0063533 3.56 0.000 .0101361 .0350406

vocational#girl | -.0500934 .0069887 -7.17 0.000 -.063791 -.0363958

|

year#schoollevel#gender |

2019#upper secondary#girl | .0028393 .0086605 0.33 0.743 -.014135 .0198135

2019#vocational#girl | .0150176 .0102118 1.47 0.141 -.0049971 .0350323

2021#upper secondary#girl | .0324402 .0086901 3.73 0.000 .015408 .0494724

2021#vocational#girl | -.0030163 .010609 -0.28 0.776 -.0238094 .0177769

2023#upper secondary#girl | .0372196 .008988 4.14 0.000 .0196035 .0548357

2023#vocational#girl | .0304501 .0111865 2.72 0.006 .008525 .0523751

|

immigrant |

One foreign-born parent | -.0369986 .0108573 -3.41 0.001 -.0582786 -.0157186

Born in Finland, foreign-born parents | -.0687038 .0228293 -3.01 0.003 -.1134484 -.0239593

Student and parents born abroad | -.2576733 .020527 -12.55 0.000 -.2979056 -.217441

|

year#immigrant |

2019#One foreign-born parent | -.0116987 .0149705 -0.78 0.435 -.0410404 .017643

2019#Born in Finland, foreign-born parents | .0137073 .0300013 0.46 0.648 -.0450943 .0725088

2019#Student and parents born abroad | .0171961 .0276199 0.62 0.534 -.0369379 .07133

2021#One foreign-born parent | -.0368902 .0149945 -2.46 0.014 -.0662788 -.0075016

2021#Born in Finland, foreign-born parents | .0040977 .0289338 0.14 0.887 -.0526114 .0608068

2021#Student and parents born abroad | -.0086821 .0271886 -0.32 0.749 -.0619709 .0446066

2023#One foreign-born parent | -.0415607 .0151867 -2.74 0.006 -.0713261 -.0117953

2023#Born in Finland, foreign-born parents | -.0112278 .0296571 -0.38 0.705 -.0693546 .046899

2023#Student and parents born abroad | .0358789 .026366 1.36 0.174 -.0157976 .0875554

|

schoollevel#immigrant |

upper secondary#One foreign-born parent | .0001945 .0199075 0.01 0.992 -.0388234 .0392125

upper secondary#Born in Finland, foreign-born parents | -.01138 .042362 -0.27 0.788 -.094408 .071648

upper secondary#Student and parents born abroad | .1129892 .0413809 2.73 0.006 .0318842 .1940943

vocational#One foreign-born parent | -.0008827 .0204638 -0.04 0.966 -.040991 .0392256

vocational#Born in Finland, foreign-born parents | .0760413 .0384821 1.98 0.048 .0006178 .1514648

vocational#Student and parents born abroad | .0251682 .0378405 0.67 0.506 -.0489978 .0993342

|

year#schoollevel#immigrant |

2019#upper secondary#One foreign-born parent | .0348697 .0264019 1.32 0.187 -.0168771 .0866165

2019 #|

upper secondary #|

Born in Finland, foreign-born parents | .0018937 .0543406 0.03 0.972 -.1046119 .1083993

2019#upper secondary#Student and parents born abroad | -.0252393 .0535072 -0.47 0.637 -.1301115 .0796329

2019#vocational#One foreign-born parent | .0183318 .02899 0.63 0.527 -.0384876 .0751511

2019#vocational#Born in Finland, foreign-born parents | -.0609624 .0563913 -1.08 0.280 -.1714874 .0495626

2019#vocational#Student and parents born abroad | .0003432 .053056 0.01 0.995 -.1036446 .104331

2021#upper secondary#One foreign-born parent | .05862 .0263383 2.23 0.026 .006998 .110242

2021 #|

upper secondary #|

Born in Finland, foreign-born parents | .0243011 .0525807 0.46 0.644 -.0787551 .1273573

2021#upper secondary#Student and parents born abroad | .0056584 .0523192 0.11 0.914 -.0968854 .1082021

2021#vocational#One foreign-born parent | .0174111 .0300335 0.58 0.562 -.0414534 .0762755

2021#vocational#Born in Finland, foreign-born parents | -.1135766 .0597013 -1.90 0.057 -.2305889 .0034358

2021#vocational#Student and parents born abroad | .0680033 .0522048 1.30 0.193 -.0343163 .1703228

2023#upper secondary#One foreign-born parent | .0207207 .0269541 0.77 0.442 -.0321083 .0735498

2023 #|

upper secondary #|

Born in Finland, foreign-born parents | .029337 .0534935 0.55 0.583 -.0755084 .1341824

2023#upper secondary#Student and parents born abroad | -.0269605 .0499372 -0.54 0.589 -.1248356 .0709147

2023#vocational#One foreign-born parent | .042982 .0303478 1.42 0.157 -.0164987 .1024626

2023#vocational#Born in Finland, foreign-born parents | -.0747941 .0585244 -1.28 0.201 -.1894997 .0399116

2023#vocational#Student and parents born abroad | .0023558 .0516523 0.05 0.964 -.0988808 .1035925

|

gender#immigrant |

girl#One foreign-born parent | -.0059601 .014329 -0.42 0.677 -.0340444 .0221243

girl#Born in Finland, foreign-born parents | .0071042 .0294307 0.24 0.809 -.0505789 .0647873

girl#Student and parents born abroad | .1164057 .0278177 4.18 0.000 .0618839 .1709274

|

year#gender#immigrant |

2019#girl#One foreign-born parent | .0245354 .0195305 1.26 0.209 -.0137437 .0628145

2019#girl#Born in Finland, foreign-born parents | -.033906 .039447 -0.86 0.390 -.1112208 .0434087

2019#girl#Student and parents born abroad | -.0535572 .0378986 -1.41 0.158 -.127837 .0207226

2021#girl#One foreign-born parent | .0471188 .0197196 2.39 0.017 .0084691 .0857685

2021#girl#Born in Finland, foreign-born parents | -.007172 .037913 -0.19 0.850 -.0814801 .0671361

2021#girl#Student and parents born abroad | -.0227339 .0372287 -0.61 0.541 -.0957007 .050233

2023#girl#One foreign-born parent | .0512914 .0202201 2.54 0.011 .0116608 .090922

2023#girl#Born in Finland, foreign-born parents | .0200309 .0389911 0.51 0.607 -.0563901 .096452

2023#girl#Student and parents born abroad | -.0273371 .0362138 -0.75 0.450 -.0983148 .0436406

|

schoollevel#gender#immigrant |

upper secondary#girl#One foreign-born parent | .0132867 .0253549 0.52 0.600 -.036408 .0629814

upper secondary #|

girl #|

Born in Finland, foreign-born parents | .0188083 .0529136 0.36 0.722 -.0849006 .1225171

upper secondary#girl#Student and parents born abroad | -.070061 .0517365 -1.35 0.176 -.1714627 .0313406

vocational#girl#One foreign-born parent | .0371475 .0284353 1.31 0.191 -.0185846 .0928796

vocational#girl#Born in Finland, foreign-born parents | -.1547696 .0594726 -2.60 0.009 -.2713337 -.0382055

vocational#girl#Student and parents born abroad | -.0902818 .0547331 -1.65 0.099 -.1975567 .0169932

|

year#schoollevel#gender#immigrant |

2019#upper secondary#girl#One foreign-born parent | -.0386122 .0335025 -1.15 0.249 -.1042759 .0270515

2019 #|

upper secondary #|

girl #|

Born in Finland, foreign-born parents | -.0024895 .0689768 -0.04 0.971 -.1376816 .1327026

2019 #|

upper secondary #|

girl #|

Student and parents born abroad | .0749349 .0674131 1.11 0.266 -.0571924 .2070622

2019#vocational#girl#One foreign-born parent | -.0561236 .0410208 -1.37 0.171 -.1365229 .0242757

2019 #|

vocational #|

girl #|

Born in Finland, foreign-born parents | .0899925 .0899213 1.00 0.317 -.0862499 .266235

2019#vocational#girl#Student and parents born abroad | .1773253 .0749965 2.36 0.018 .0303349 .3243156

2021#upper secondary#girl#One foreign-born parent | -.0801372 .0336226 -2.38 0.017 -.1460364 -.0142381

2021 #|

upper secondary #|

girl #|

Born in Finland, foreign-born parents | -.0733014 .0673914 -1.09 0.277 -.2053862 .0587833

2021 #|

upper secondary #|

girl #|

Student and parents born abroad | .0032356 .0662473 0.05 0.961 -.1266067 .1330779

2021#vocational#girl#One foreign-born parent | -.0334349 .0425714 -0.79 0.432 -.1168734 .0500035

2021 #|

vocational #|

girl #|

Born in Finland, foreign-born parents | .1446581 .0947486 1.53 0.127 -.0410458 .3303619

2021#vocational#girl#Student and parents born abroad | .0666391 .0765238 0.87 0.384 -.0833449 .2166231

2023#upper secondary#girl#One foreign-born parent | -.0121741 .034426 -0.35 0.724 -.0796478 .0552996

2023 #|

upper secondary #|

girl #|

Born in Finland, foreign-born parents | -.0459155 .0676993 -0.68 0.498 -.1786035 .0867726

2023 #|

upper secondary #|

girl #|

Student and parents born abroad | -.0063966 .0637558 -0.10 0.920 -.1313557 .1185625

2023#vocational#girl#One foreign-born parent | -.0741401 .0434959 -1.70 0.088 -.1593905 .0111103

2023 #|

vocational #|

girl #|

Born in Finland, foreign-born parents | .2581112 .0858151 3.01 0.003 .0899167 .4263058

2023#vocational#girl#Student and parents born abroad | .1002199 .075377 1.33 0.184 -.0475163 .247956

|

_cons | .7946072 .00388 204.79 0.000 .7870025 .8022119

------------------------------------------------------------------------------------------------------------------------

.

. testparm year#gender#degree

( 1) [closefriend3]2019.year#2.gender#1.degree = 0

( 2) [closefriend3]2021.year#2.gender#1.degree = 0

( 3) [closefriend3]2023.year#2.gender#1.degree = 0

chi2( 3) = 10.46

Prob > chi2 = 0.0150

. margins year#gender#degree

Predictive margins Number of obs = 556,754

Model VCE: Robust

Expression: Predicted number of events, predict()

------------------------------------------------------------------------------------

| Delta-method

| Margin std. err. z P>|z| [95% conf. interval]

-------------------+----------------------------------------------------------------

year#gender#degree |

2017#boy#0 | 2.198139 .0064195 342.42 0.000 2.185557 2.210721

2017#boy#1 | 2.26906 .0059875 378.97 0.000 2.257325 2.280796

2017#girl#0 | 2.185059 .0053315 409.84 0.000 2.174609 2.195508

2017#girl#1 | 2.264647 .0053698 421.74 0.000 2.254123 2.275172

2019#boy#0 | 2.190582 .006287 348.43 0.000 2.17826 2.202904

2019#boy#1 | 2.228494 .0054254 410.75 0.000 2.21786 2.239128

2019#girl#0 | 2.134589 .0052829 404.05 0.000 2.124235 2.144943

2019#girl#1 | 2.229489 .0048108 463.43 0.000 2.22006 2.238918

2021#boy#0 | 2.153194 .0063126 341.10 0.000 2.140822 2.165567

2021#boy#1 | 2.209528 .0052189 423.37 0.000 2.199299 2.219757

2021#girl#0 | 2.020214 .005314 380.17 0.000 2.009799 2.030629

2021#girl#1 | 2.093402 .0046864 446.69 0.000 2.084217 2.102587

2023#boy#0 | 2.126236 .0068033 312.53 0.000 2.112901 2.13957

2023#boy#1 | 2.182825 .0053706 406.44 0.000 2.172299 2.193351

2023#girl#0 | 1.988773 .0059509 334.20 0.000 1.97711 2.000437

2023#girl#1 | 2.063558 .004898 421.30 0.000 2.053958 2.073158

------------------------------------------------------------------------------------

. testparm year#gender#urbanrural

( 1) [closefriend3]2019.year#2.gender#2.urbanrural = 0

( 2) [closefriend3]2019.year#2.gender#3.urbanrural = 0

( 3) [closefriend3]2021.year#2.gender#2.urbanrural = 0

( 4) [closefriend3]2021.year#2.gender#3.urbanrural = 0

( 5) [closefriend3]2023.year#2.gender#2.urbanrural = 0

( 6) [closefriend3]2023.year#2.gender#3.urbanrural = 0

chi2( 6) = 9.53

Prob > chi2 = 0.1458

. testparm year#schoollevel#gender#immigrant

( 1) [closefriend3]2019.year#20.schoollevel#2.gender#2.immigrant = 0

( 2) [closefriend3]2019.year#20.schoollevel#2.gender#3.immigrant = 0

( 3) [closefriend3]2019.year#20.schoollevel#2.gender#4.immigrant = 0

( 4) [closefriend3]2019.year#30.schoollevel#2.gender#2.immigrant = 0

( 5) [closefriend3]2019.year#30.schoollevel#2.gender#3.immigrant = 0

( 6) [closefriend3]2019.year#30.schoollevel#2.gender#4.immigrant = 0

( 7) [closefriend3]2021.year#20.schoollevel#2.gender#2.immigrant = 0

( 8) [closefriend3]2021.year#20.schoollevel#2.gender#3.immigrant = 0

( 9) [closefriend3]2021.year#20.schoollevel#2.gender#4.immigrant = 0

(10) [closefriend3]2021.year#30.schoollevel#2.gender#2.immigrant = 0

(11) [closefriend3]2021.year#30.schoollevel#2.gender#3.immigrant = 0

(12) [closefriend3]2021.year#30.schoollevel#2.gender#4.immigrant = 0

(13) [closefriend3]2023.year#20.schoollevel#2.gender#2.immigrant = 0

(14) [closefriend3]2023.year#20.schoollevel#2.gender#3.immigrant = 0

(15) [closefriend3]2023.year#20.schoollevel#2.gender#4.immigrant = 0

(16) [closefriend3]2023.year#30.schoollevel#2.gender#2.immigrant = 0

(17) [closefriend3]2023.year#30.schoollevel#2.gender#3.immigrant = 0

(18) [closefriend3]2023.year#30.schoollevel#2.gender#4.immigrant = 0

chi2( 18) = 31.76

Prob > chi2 = 0.0235

. poisson closefriend3 i.year##i.gender##i.degree i.year##i.urbanrural i.year##i.schoollevel##i.gender##i.immigrant, vce(ro

> bust)

Iteration 0: Log pseudolikelihood = -872571.14

Iteration 1: Log pseudolikelihood = -872571.14

Poisson regression Number of obs = 556,754

Wald chi2(111) = 6414.22

Prob > chi2 = 0.0000

Log pseudolikelihood = -872571.14 Pseudo R2 = 0.0020

------------------------------------------------------------------------------------------------------------------------

| Robust

closefriend3 | Coefficient std. err. z P>|z| [95% conf. interval]

-------------------------------------------------------+----------------------------------------------------------------

year |

2019 | .0018055 .0050429 0.36 0.720 -.0080784 .0116893

2021 | -.0135635 .0050485 -2.69 0.007 -.0234583 -.0036686

2023 | -.0246649 .0052462 -4.70 0.000 -.0349472 -.0143825

|

gender |

girl | -.0105583 .0045325 -2.33 0.020 -.0194418 -.0016748

|

year#gender |

2019#girl | -.022438 .0063444 -3.54 0.000 -.0348727 -.0100033

2021#girl | -.0660148 .0064264 -10.27 0.000 -.0786103 -.0534193

2023#girl | -.08038 .0067563 -11.90 0.000 -.0936221 -.0671378

|

1.degree | .0304179 .0039123 7.77 0.000 .02275 .0380858

|

year#degree |

2019 1 | -.0146924 .0054478 -2.70 0.007 -.0253699 -.0040148

2021 1 | -.0051743 .0054582 -0.95 0.343 -.0158721 .0055235

2023 1 | -.0060533 .0056576 -1.07 0.285 -.0171419 .0050353

|

gender#degree |

girl#1 | .0065918 .0051727 1.27 0.203 -.0035466 .0167302

|

year#gender#degree |

2019#girl#1 | .0225036 .0071944 3.13 0.002 .0084029 .0366043

2021#girl#1 | .0042775 .0072812 0.59 0.557 -.0099934 .0185484

2023#girl#1 | .0078068 .0076095 1.03 0.305 -.0071077 .0227212

|

urbanrural |

semi-urban | -.0095093 .0033758 -2.82 0.005 -.0161257 -.0028929

rural | -.0293394 .004058 -7.23 0.000 -.037293 -.0213859

|

year#urbanrural |

2019#semi-urban | -.0012658 .0047046 -0.27 0.788 -.0104866 .007955

2019#rural | .007812 .0056503 1.38 0.167 -.0032623 .0188863

2021#semi-urban | .003069 .0047489 0.65 0.518 -.0062388 .0123767

2021#rural | .0122933 .0056964 2.16 0.031 .0011285 .0234581

2023#semi-urban | -.000808 .0049542 -0.16 0.870 -.0105181 .0089021

2023#rural | -.000085 .0059923 -0.01 0.989 -.0118298 .0116597

|

schoollevel |

upper secondary | -.0031368 .0049932 -0.63 0.530 -.0129232 .0066497

vocational | .0435008 .0047209 9.21 0.000 .0342479 .0527537

|

year#schoollevel |

2019#upper secondary | -.0096495 .0067992 -1.42 0.156 -.0229758 .0036768

2019#vocational | -.0239445 .006874 -3.48 0.000 -.0374172 -.0104718

2021#upper secondary | -.0179217 .0067718 -2.65 0.008 -.0311943 -.0046492

2021#vocational | -.0209699 .0069462 -3.02 0.003 -.0345843 -.0073555

2023#upper secondary | -.0085844 .0069382 -1.24 0.216 -.0221831 .0050143

2023#vocational | -.0271672 .0073788 -3.68 0.000 -.0416294 -.012705

|

schoollevel#gender |

upper secondary#girl | .023746 .0063455 3.74 0.000 .0113091 .0361829

vocational#girl | -.0464372 .0069131 -6.72 0.000 -.0599866 -.0328879

|

year#schoollevel#gender |

2019#upper secondary#girl | .0027127 .008649 0.31 0.754 -.014239 .0196644

2019#vocational#girl | .0140926 .0101268 1.39 0.164 -.0057556 .0339407

2021#upper secondary#girl | .0316922 .0086779 3.65 0.000 .0146837 .0487006

2021#vocational#girl | -.0062386 .0105261 -0.59 0.553 -.0268694 .0143923

2023#upper secondary#girl | .037813 .0089719 4.21 0.000 .0202284 .0553977

2023#vocational#girl | .0300661 .0111114 2.71 0.007 .0082881 .051844

|

immigrant |

One foreign-born parent | -.037859 .0108533 -3.49 0.000 -.0591312 -.0165869

Born in Finland, foreign-born parents | -.0711721 .0228104 -3.12 0.002 -.1158797 -.0264645

Student and parents born abroad | -.2589616 .0205196 -12.62 0.000 -.2991794 -.2187438

|

year#immigrant |

2019#One foreign-born parent | -.0117087 .0149654 -0.78 0.434 -.0410404 .017623

2019#Born in Finland, foreign-born parents | .0138379 .0299645 0.46 0.644 -.0448914 .0725671

2019#Student and parents born abroad | .0172125 .0276077 0.62 0.533 -.0368975 .0713225

2021#One foreign-born parent | -.0364985 .0149898 -2.43 0.015 -.065878 -.007119

2021#Born in Finland, foreign-born parents | .0054025 .028903 0.19 0.852 -.0512463 .0620513

2021#Student and parents born abroad | -.0079689 .0271769 -0.29 0.769 -.0612347 .0452969

2023#One foreign-born parent | -.0420059 .0151816 -2.77 0.006 -.0717613 -.0122506

2023#Born in Finland, foreign-born parents | -.0114827 .0296326 -0.39 0.698 -.0695616 .0465962

2023#Student and parents born abroad | .0353821 .026359 1.34 0.179 -.0162806 .0870447

|

schoollevel#immigrant |

upper secondary#One foreign-born parent | .0002441 .0199107 0.01 0.990 -.0387801 .0392684

upper secondary#Born in Finland, foreign-born parents | -.0111846 .0423448 -0.26 0.792 -.094179 .0718097

upper secondary#Student and parents born abroad | .1129005 .041357 2.73 0.006 .0318423 .1939587

vocational#One foreign-born parent | -.0005278 .0204571 -0.03 0.979 -.040623 .0395674

vocational#Born in Finland, foreign-born parents | .0771838 .0384729 2.01 0.045 .0017783 .1525894

vocational#Student and parents born abroad | .0258985 .0378369 0.68 0.494 -.0482605 .1000575

|

year#schoollevel#immigrant |

2019#upper secondary#One foreign-born parent | .0347628 .0264028 1.32 0.188 -.0169858 .0865114

2019 #|

upper secondary #|

Born in Finland, foreign-born parents | .0017867 .0543191 0.03 0.974 -.1046768 .1082502

2019#upper secondary#Student and parents born abroad | -.0253239 .0534954 -0.47 0.636 -.1301728 .0795251

2019#vocational#One foreign-born parent | .0180995 .0289858 0.62 0.532 -.0387115 .0749106

2019#vocational#Born in Finland, foreign-born parents | -.0618123 .0563681 -1.10 0.273 -.1722918 .0486671

2019#vocational#Student and parents born abroad | .0001465 .0530426 0.00 0.998 -.1038151 .104108

2021#upper secondary#One foreign-born parent | .0586368 .0263403 2.23 0.026 .0070108 .1102628

2021 #|

upper secondary #|

Born in Finland, foreign-born parents | .0242287 .0525649 0.46 0.645 -.0787965 .1272539

2021#upper secondary#Student and parents born abroad | .0054758 .0522993 0.10 0.917 -.0970289 .1079806

2021#vocational#One foreign-born parent | .0169278 .0300256 0.56 0.573 -.0419213 .0757768

2021#vocational#Born in Finland, foreign-born parents | -.1141791 .0596726 -1.91 0.056 -.2311353 .0027771

2021#vocational#Student and parents born abroad | .0672529 .0521952 1.29 0.198 -.0350479 .1695537

2023#upper secondary#One foreign-born parent | .0209368 .0269547 0.78 0.437 -.0318936 .0737671

2023 #|

upper secondary #|

Born in Finland, foreign-born parents | .0295498 .0534759 0.55 0.581 -.0752612 .1343607

2023#upper secondary#Student and parents born abroad | -.0256866 .0499214 -0.51 0.607 -.1235308 .0721576

2023#vocational#One foreign-born parent | .0433993 .0303473 1.43 0.153 -.0160804 .1028789

2023#vocational#Born in Finland, foreign-born parents | -.0754724 .0584998 -1.29 0.197 -.1901299 .0391851

2023#vocational#Student and parents born abroad | .0021884 .0516458 0.04 0.966 -.0990355 .1034124

|

gender#immigrant |

girl#One foreign-born parent | -.0042243 .0143216 -0.29 0.768 -.032294 .0238454

girl#Born in Finland, foreign-born parents | .0120925 .0293919 0.41 0.681 -.0455146 .0696996

girl#Student and parents born abroad | .1192159 .0278058 4.29 0.000 .0647176 .1737142

|

year#gender#immigrant |

2019#girl#One foreign-born parent | .0244838 .0195195 1.25 0.210 -.0137736 .0627412

2019#girl#Born in Finland, foreign-born parents | -.0340264 .039381 -0.86 0.388 -.1112117 .0431589

2019#girl#Student and parents born abroad | -.053445 .0378781 -1.41 0.158 -.1276848 .0207948

2021#girl#One foreign-born parent | .0462921 .0197096 2.35 0.019 .007662 .0849223

2021#girl#Born in Finland, foreign-born parents | -.0097299 .0378519 -0.26 0.797 -.0839182 .0644584

2021#girl#Student and parents born abroad | -.0242411 .0372084 -0.65 0.515 -.0971681 .048686

2023#girl#One foreign-born parent | .0518633 .0202077 2.57 0.010 .0122569 .0914697

2023#girl#Born in Finland, foreign-born parents | .020509 .0389366 0.53 0.598 -.0558053 .0968233

2023#girl#Student and parents born abroad | -.0262456 .0361934 -0.73 0.468 -.0971834 .0446921

|

schoollevel#gender#immigrant |

upper secondary#girl#One foreign-born parent | .0131139 .025359 0.52 0.605 -.0365888 .0628166

upper secondary #|

girl #|

Born in Finland, foreign-born parents | .0183322 .0528997 0.35 0.729 -.0853493 .1220137

upper secondary#girl#Student and parents born abroad | -.069843 .0517214 -1.35 0.177 -.171215 .0315291

vocational#girl#One foreign-born parent | .036313 .0284357 1.28 0.202 -.0194199 .0920459

vocational#girl#Born in Finland, foreign-born parents | -.157273 .0594689 -2.64 0.008 -.27383 -.040716

vocational#girl#Student and parents born abroad | -.0917997 .0547273 -1.68 0.093 -.1990632 .0154638

|

year#schoollevel#gender#immigrant |

2019#upper secondary#girl#One foreign-born parent | -.0384669 .033504 -1.15 0.251 -.1041336 .0271997

2019 #|

upper secondary #|

girl #|

Born in Finland, foreign-born parents | -.0027185 .0689607 -0.04 0.969 -.137879 .1324419

2019 #|

upper secondary #|

girl #|

Student and parents born abroad | .0744948 .0674108 1.11 0.269 -.0576279 .2066175

2019#vocational#girl#One foreign-born parent | -.056042 .0410228 -1.37 0.172 -.1364452 .0243613

2019 #|

vocational #|

girl #|

Born in Finland, foreign-born parents | .091074 .0899273 1.01 0.311 -.0851802 .2673282

2019#vocational#girl#Student and parents born abroad | .1776217 .0749842 2.37 0.018 .0306553 .3245881

2021#upper secondary#girl#One foreign-born parent | -.0801403 .0336256 -2.38 0.017 -.1460452 -.0142353

2021 #|

upper secondary #|

girl #|

Born in Finland, foreign-born parents | -.0730653 .067378 -1.08 0.278 -.2051238 .0589933

2021 #|

upper secondary #|

girl #|

Student and parents born abroad | .003438 .0662359 0.05 0.959 -.126382 .133258

2021#vocational#girl#One foreign-born parent | -.0326149 .0425706 -0.77 0.444 -.1160517 .0508219

2021 #|

vocational #|

girl #|

Born in Finland, foreign-born parents | .146256 .0947318 1.54 0.123 -.039415 .331927

2021#vocational#girl#Student and parents born abroad | .0678499 .0765213 0.89 0.375 -.0821291 .2178288

2023#upper secondary#girl#One foreign-born parent | -.0124131 .0344277 -0.36 0.718 -.0798902 .0550639

2023 #|

upper secondary #|

girl #|

Born in Finland, foreign-born parents | -.0461494 .0676878 -0.68 0.495 -.178815 .0865161

2023 #|

upper secondary #|

girl #|

Student and parents born abroad | -.0096833 .0637426 -0.15 0.879 -.1346165 .1152499

2023#vocational#girl#One foreign-born parent | -.074687 .043499 -1.72 0.086 -.1599434 .0105694

2023 #|

vocational #|

girl #|

Born in Finland, foreign-born parents | .258804 .0858 3.02 0.003 .0906391 .4269689

2023#vocational#girl#Student and parents born abroad | .0998912 .0753701 1.33 0.185 -.0478315 .2476138

|

_cons | .7989231 .0036336 219.87 0.000 .7918013 .8060449

------------------------------------------------------------------------------------------------------------------------

. testparm year#gender#degree

( 1) [closefriend3]2019.year#2.gender#1.degree = 0

( 2) [closefriend3]2021.year#2.gender#1.degree = 0

( 3) [closefriend3]2023.year#2.gender#1.degree = 0

chi2( 3) = 11.21

Prob > chi2 = 0.0106

. testparm year#schoollevel#gender#immigrant

( 1) [closefriend3]2019.year#20.schoollevel#2.gender#2.immigrant = 0

( 2) [closefriend3]2019.year#20.schoollevel#2.gender#3.immigrant = 0

( 3) [closefriend3]2019.year#20.schoollevel#2.gender#4.immigrant = 0

( 4) [closefriend3]2019.year#30.schoollevel#2.gender#2.immigrant = 0

( 5) [closefriend3]2019.year#30.schoollevel#2.gender#3.immigrant = 0

( 6) [closefriend3]2019.year#30.schoollevel#2.gender#4.immigrant = 0

( 7) [closefriend3]2021.year#20.schoollevel#2.gender#2.immigrant = 0

( 8) [closefriend3]2021.year#20.schoollevel#2.gender#3.immigrant = 0

( 9) [closefriend3]2021.year#20.schoollevel#2.gender#4.immigrant = 0

(10) [closefriend3]2021.year#30.schoollevel#2.gender#2.immigrant = 0

(11) [closefriend3]2021.year#30.schoollevel#2.gender#3.immigrant = 0

(12) [closefriend3]2021.year#30.schoollevel#2.gender#4.immigrant = 0

(13) [closefriend3]2023.year#20.schoollevel#2.gender#2.immigrant = 0

(14) [closefriend3]2023.year#20.schoollevel#2.gender#3.immigrant = 0

(15) [closefriend3]2023.year#20.schoollevel#2.gender#4.immigrant = 0

(16) [closefriend3]2023.year#30.schoollevel#2.gender#2.immigrant = 0

(17) [closefriend3]2023.year#30.schoollevel#2.gender#3.immigrant = 0

(18) [closefriend3]2023.year#30.schoollevel#2.gender#4.immigrant = 0

chi2( 18) = 31.99

Prob > chi2 = 0.0221

. testparm year#urbanrural

( 1) [closefriend3]2019.year#2.urbanrural = 0

( 2) [closefriend3]2019.year#3.urbanrural = 0

( 3) [closefriend3]2021.year#2.urbanrural = 0

( 4) [closefriend3]2021.year#3.urbanrural = 0

( 5) [closefriend3]2023.year#2.urbanrural = 0

( 6) [closefriend3]2023.year#3.urbanrural = 0

chi2( 6) = 7.34

Prob > chi2 = 0.2904

. poisson closefriend3 i.year##i.gender##i.degree i.urbanrural i.year##i.schoollevel##i.gender##i.immigrant, vce(robust)

Iteration 0: Log pseudolikelihood = -872572.93

Iteration 1: Log pseudolikelihood = -872572.93

Poisson regression Number of obs = 556,754

Wald chi2(105) = 6402.65

Prob > chi2 = 0.0000

Log pseudolikelihood = -872572.93 Pseudo R2 = 0.0020

------------------------------------------------------------------------------------------------------------------------

| Robust

closefriend3 | Coefficient std. err. z P>|z| [95% conf. interval]

-------------------------------------------------------+----------------------------------------------------------------

year |

2019 | .0030447 .0047688 0.64 0.523 -.0063019 .0123913

2021 | -.0104952 .0047757 -2.20 0.028 -.0198554 -.001135

2023 | -.0247896 .0049592 -5.00 0.000 -.0345096 -.0150697

|

gender |

girl | -.0105205 .0045321 -2.32 0.020 -.0194032 -.0016377

|

year#gender |

2019#girl | -.022482 .0063441 -3.54 0.000 -.0349162 -.0100477

2021#girl | -.0660743 .0064265 -10.28 0.000 -.0786701 -.0534786

2023#girl | -.0803786 .0067558 -11.90 0.000 -.0936198 -.0671375

|

1.degree | .0307988 .0038977 7.90 0.000 .0231595 .0384381

|

year#degree |

2019 1 | -.0151526 .0054191 -2.80 0.005 -.0257739 -.0045314

2021 1 | -.0062437 .0054306 -1.15 0.250 -.0168874 .0044

2023 1 | -.0059687 .0056282 -1.06 0.289 -.0169998 .0050624

|

gender#degree |

girl#1 | .0065908 .0051726 1.27 0.203 -.0035474 .0167289

|

year#gender#degree |

2019#girl#1 | .0225031 .0071943 3.13 0.002 .0084025 .0366037

2021#girl#1 | .0042878 .0072813 0.59 0.556 -.0099832 .0185588

2023#girl#1 | .0078183 .0076093 1.03 0.304 -.0070957 .0227323

|

urbanrural |

semi-urban | -.0092137 .0017016 -5.41 0.000 -.0125488 -.0058786

rural | -.0240491 .0020469 -11.75 0.000 -.0280609 -.0200373

|

schoollevel |

upper secondary | -.0029403 .0049913 -0.59 0.556 -.012723 .0068425

vocational | .0441922 .0047011 9.40 0.000 .0349782 .0534062

|

year#schoollevel |

2019#upper secondary | -.0098824 .0067954 -1.45 0.146 -.0232011 .0034363

2019#vocational | -.0248846 .006844 -3.64 0.000 -.0382986 -.0114707

2021#upper secondary | -.0183964 .0067683 -2.72 0.007 -.031662 -.0051307

2021#vocational | -.0224203 .0069172 -3.24 0.001 -.0359777 -.0088629

2023#upper secondary | -.0085632 .0069329 -1.24 0.217 -.0221514 .005025

2023#vocational | -.0273699 .0073513 -3.72 0.000 -.0417781 -.0129617

|

schoollevel#gender |

upper secondary#girl | .0237225 .0063455 3.74 0.000 .0112855 .0361596

vocational#girl | -.0464867 .0069107 -6.73 0.000 -.0600314 -.032942

|

year#schoollevel#gender |

2019#upper secondary#girl | .0027577 .0086491 0.32 0.750 -.0141942 .0197097

2019#vocational#girl | .0142502 .010124 1.41 0.159 -.0055925 .0340929

2021#upper secondary#girl | .0316919 .0086776 3.65 0.000 .0146841 .0486997

2021#vocational#girl | -.0062187 .0105229 -0.59 0.555 -.0268432 .0144058

2023#upper secondary#girl | .03783 .0089721 4.22 0.000 .020245 .055415

2023#vocational#girl | .0301595 .0111061 2.72 0.007 .008392 .051927

|

immigrant |

One foreign-born parent | -.0376586 .0108522 -3.47 0.001 -.0589285 -.0163888

Born in Finland, foreign-born parents | -.0705497 .0228041 -3.09 0.002 -.1152449 -.0258544

Student and parents born abroad | -.2586231 .0205174 -12.61 0.000 -.2988365 -.2184097

|

year#immigrant |

2019#One foreign-born parent | -.0119392 .0149624 -0.80 0.425 -.041265 .0173867

2019#Born in Finland, foreign-born parents | .0131699 .0299478 0.44 0.660 -.0455268 .0718666

2019#Student and parents born abroad | .0168574 .0276026 0.61 0.541 -.0372428 .0709575

2021#One foreign-born parent | -.0371132 .0149863 -2.48 0.013 -.0664858 -.0077406

2021#Born in Finland, foreign-born parents | .0036312 .0288843 0.13 0.900 -.052981 .0602434

2021#Student and parents born abroad | -.0088685 .0271686 -0.33 0.744 -.0621181 .044381

2023#One foreign-born parent | -.0419136 .0151771 -2.76 0.006 -.0716602 -.0121671

2023#Born in Finland, foreign-born parents | -.0114897 .0296161 -0.39 0.698 -.0695361 .0465567

2023#Student and parents born abroad | .0354667 .0263517 1.35 0.178 -.0161816 .087115

|

schoollevel#immigrant |

upper secondary#One foreign-born parent | .0002353 .0199093 0.01 0.991 -.0387861 .0392567

upper secondary#Born in Finland, foreign-born parents | -.0112578 .0423502 -0.27 0.790 -.0942626 .0717471

upper secondary#Student and parents born abroad | .1128537 .0413628 2.73 0.006 .0317842 .1939233

vocational#One foreign-born parent | -.0006751 .0204579 -0.03 0.974 -.0407718 .0394217

vocational#Born in Finland, foreign-born parents | .0767786 .0384729 2.00 0.046 .0013732 .1521841

vocational#Student and parents born abroad | .0256427 .0378358 0.68 0.498 -.048514 .0997994

|

year#schoollevel#immigrant |

2019#upper secondary#One foreign-born parent | .03478 .0264017 1.32 0.188 -.0169664 .0865264

2019 #|

upper secondary #|

Born in Finland, foreign-born parents | .0018632 .0543225 0.03 0.973 -.104607 .1083334

2019#upper secondary#Student and parents born abroad | -.0252678 .0535013 -0.47 0.637 -.1301284 .0795928

2019#vocational#One foreign-born parent | .0183894 .0289863 0.63 0.526 -.0384227 .0752015

2019#vocational#Born in Finland, foreign-born parents | -.061236 .0563697 -1.09 0.277 -.1717186 .0492467

2019#vocational#Student and parents born abroad | .0004417 .0530427 0.01 0.993 -.1035202 .1044035

2021#upper secondary#One foreign-born parent | .0588049 .0263391 2.23 0.026 .0071812 .1104287

2021 #|

upper secondary #|

Born in Finland, foreign-born parents | .0245538 .0525681 0.47 0.640 -.0784779 .1275855

2021#upper secondary#Student and parents born abroad | .0056797 .0523044 0.11 0.914 -.096835 .1081944

2021#vocational#One foreign-born parent | .0172982 .0300259 0.58 0.565 -.0415514 .0761478

2021#vocational#Born in Finland, foreign-born parents | -.1129798 .0596532 -1.89 0.058 -.2298979 .0039384

2021#vocational#Student and parents born abroad | .0677432 .0521927 1.30 0.194 -.0345526 .170039

2023#upper secondary#One foreign-born parent | .0208561 .0269543 0.77 0.439 -.0319734 .0736856

2023 #|

upper secondary #|

Born in Finland, foreign-born parents | .029537 .0534798 0.55 0.581 -.0752815 .1343554

2023#upper secondary#Student and parents born abroad | -.0259898 .049924 -0.52 0.603 -.123839 .0718594

2023#vocational#One foreign-born parent | .0432755 .0303447 1.43 0.154 -.0161991 .10275

2023#vocational#Born in Finland, foreign-born parents | -.0752785 .0585019 -1.29 0.198 -.18994 .0393831

2023#vocational#Student and parents born abroad | .0022403 .0516442 0.04 0.965 -.0989804 .103461

|

gender#immigrant |

girl#One foreign-born parent | -.0041872 .0143226 -0.29 0.770 -.0322589 .0238846

girl#Born in Finland, foreign-born parents | .012166 .0293943 0.41 0.679 -.0454458 .0697779

girl#Student and parents born abroad | .1192954 .0278077 4.29 0.000 .0647933 .1737974

|

year#gender#immigrant |

2019#girl#One foreign-born parent | .0244519 .0195204 1.25 0.210 -.0138075 .0627112

2019#girl#Born in Finland, foreign-born parents | -.0340929 .0393825 -0.87 0.387 -.1112811 .0430953

2019#girl#Student and parents born abroad | -.0534997 .0378793 -1.41 0.158 -.1277417 .0207423

2021#girl#One foreign-born parent | .0462757 .0197108 2.35 0.019 .0076432 .0849082

2021#girl#Born in Finland, foreign-born parents | -.0098004 .0378526 -0.26 0.796 -.0839901 .0643892

2021#girl#Student and parents born abroad | -.0244429 .0372067 -0.66 0.511 -.0973667 .048481

2023#girl#One foreign-born parent | .0517593 .0202081 2.56 0.010 .0121522 .0913664

2023#girl#Born in Finland, foreign-born parents | .0204469 .0389386 0.53 0.600 -.0558713 .0967651

2023#girl#Student and parents born abroad | -.0262456 .0361938 -0.73 0.468 -.0971841 .044693

|

schoollevel#gender#immigrant |

upper secondary#girl#One foreign-born parent | .0130606 .0253591 0.52 0.607 -.0366422 .0627634

upper secondary #|

girl #|

Born in Finland, foreign-born parents | .0183447 .0529048 0.35 0.729 -.0853468 .1220362

upper secondary#girl#Student and parents born abroad | -.0698158 .0517269 -1.35 0.177 -.1711987 .031567

vocational#girl#One foreign-born parent | .0362854 .0284371 1.28 0.202 -.0194503 .092021

vocational#girl#Born in Finland, foreign-born parents | -.1573948 .0594712 -2.65 0.008 -.2739562 -.0408335

vocational#girl#Student and parents born abroad | -.0917197 .0547259 -1.68 0.094 -.1989805 .015541

|

year#schoollevel#gender#immigrant |

2019#upper secondary#girl#One foreign-born parent | -.0384179 .0335043 -1.15 0.252 -.1040851 .0272494

2019 #|

upper secondary #|

girl #|

Born in Finland, foreign-born parents | -.0027608 .0689635 -0.04 0.968 -.1379269 .1324053

2019 #|

upper secondary #|

girl #|

Student and parents born abroad | .0744613 .0674159 1.10 0.269 -.0576715 .2065941

2019#vocational#girl#One foreign-born parent | -.0560839 .0410238 -1.37 0.172 -.136489 .0243212

2019 #|

vocational #|

girl #|

Born in Finland, foreign-born parents | .0910871 .0899317 1.01 0.311 -.0851757 .26735

2019#vocational#girl#Student and parents born abroad | .1774957 .0749834 2.37 0.018 .0305309 .3244605

2021#upper secondary#girl#One foreign-born parent | -.0802535 .0336259 -2.39 0.017 -.146159 -.014348

2021 #|

upper secondary #|

girl #|

Born in Finland, foreign-born parents | -.0731372 .0673812 -1.09 0.278 -.205202 .0589276

2021 #|

upper secondary #|

girl #|

Student and parents born abroad | .0034005 .0662399 0.05 0.959 -.1264273 .1332284

2021#vocational#girl#One foreign-born parent | -.0325937 .0425706 -0.77 0.444 -.1160305 .0508431

2021 #|

vocational #|

girl #|

Born in Finland, foreign-born parents | .146049 .0947173 1.54 0.123 -.0395934 .3316914

2021#vocational#girl#Student and parents born abroad | .067986 .0765156 0.89 0.374 -.0819818 .2179538

2023#upper secondary#girl#One foreign-born parent | -.0122984 .0344285 -0.36 0.721 -.079777 .0551803

2023 #|

upper secondary #|

girl #|

Born in Finland, foreign-born parents | -.0461605 .0676924 -0.68 0.495 -.1788352 .0865143

2023 #|

upper secondary #|

girl #|

Student and parents born abroad | -.0098987 .0637456 -0.16 0.877 -.1348377 .1150404

2023#vocational#girl#One foreign-born parent | -.074568 .0434982 -1.71 0.086 -.1598228 .0106868

2023 #|

vocational #|

girl #|

Born in Finland, foreign-born parents | .2587666 .0858036 3.02 0.003 .0905947 .4269385

2023#vocational#girl#Student and parents born abroad | .0997546 .0753685 1.32 0.186 -.0479649 .2474741

|

_cons | .7977939 .0034863 228.83 0.000 .7909608 .804627

------------------------------------------------------------------------------------------------------------------------

. testparm year#gender#degree

( 1) [closefriend3]2019.year#2.gender#1.degree = 0

( 2) [closefriend3]2021.year#2.gender#1.degree = 0

( 3) [closefriend3]2023.year#2.gender#1.degree = 0

chi2( 3) = 11.21

Prob > chi2 = 0.0106

. testparm year#schoollevel#gender#immigrant

( 1) [closefriend3]2019.year#20.schoollevel#2.gender#2.immigrant = 0

( 2) [closefriend3]2019.year#20.schoollevel#2.gender#3.immigrant = 0

( 3) [closefriend3]2019.year#20.schoollevel#2.gender#4.immigrant = 0

( 4) [closefriend3]2019.year#30.schoollevel#2.gender#2.immigrant = 0

( 5) [closefriend3]2019.year#30.schoollevel#2.gender#3.immigrant = 0

( 6) [closefriend3]2019.year#30.schoollevel#2.gender#4.immigrant = 0

( 7) [closefriend3]2021.year#20.schoollevel#2.gender#2.immigrant = 0

( 8) [closefriend3]2021.year#20.schoollevel#2.gender#3.immigrant = 0

( 9) [closefriend3]2021.year#20.schoollevel#2.gender#4.immigrant = 0

(10) [closefriend3]2021.year#30.schoollevel#2.gender#2.immigrant = 0

(11) [closefriend3]2021.year#30.schoollevel#2.gender#3.immigrant = 0

(12) [closefriend3]2021.year#30.schoollevel#2.gender#4.immigrant = 0

(13) [closefriend3]2023.year#20.schoollevel#2.gender#2.immigrant = 0

(14) [closefriend3]2023.year#20.schoollevel#2.gender#3.immigrant = 0

(15) [closefriend3]2023.year#20.schoollevel#2.gender#4.immigrant = 0

(16) [closefriend3]2023.year#30.schoollevel#2.gender#2.immigrant = 0

(17) [closefriend3]2023.year#30.schoollevel#2.gender#3.immigrant = 0

(18) [closefriend3]2023.year#30.schoollevel#2.gender#4.immigrant = 0

chi2( 18) = 32.01

Prob > chi2 = 0.0219

. margins year#gender#degree

Predictive margins Number of obs = 556,754

Model VCE: Robust

Expression: Predicted number of events, predict()

------------------------------------------------------------------------------------

| Delta-method

| Margin std. err. z P>|z| [95% conf. interval]

-------------------+----------------------------------------------------------------

year#gender#degree |

2017#boy#0 | 2.199612 .0063781 344.87 0.000 2.187111 2.212113

2017#boy#1 | 2.268412 .0059688 380.04 0.000 2.256713 2.28011

2017#girl#0 | 2.182909 .0053046 411.52 0.000 2.172513 2.193306

2017#girl#1 | 2.266072 .00535 423.57 0.000 2.255587 2.276558

2019#boy#0 | 2.192469 .0062562 350.45 0.000 2.180207 2.204731

2019#boy#1 | 2.227043 .0053908 413.12 0.000 2.216477 2.237608

2019#girl#0 | 2.133312 .0052632 405.32 0.000 2.122996 2.143628

2019#girl#1 | 2.230924 .0047804 466.68 0.000 2.221554 2.240293

2021#boy#0 | 2.154825 .0062873 342.73 0.000 2.142502 2.167148

2021#boy#1 | 2.208392 .0051873 425.73 0.000 2.198225 2.218559

2021#girl#0 | 2.020431 .0052961 381.50 0.000 2.010051 2.030811

2021#girl#1 | 2.093305 .0046574 449.46 0.000 2.084177 2.102434

2023#boy#0 | 2.128071 .006773 314.20 0.000 2.114796 2.141345

2023#boy#1 | 2.181572 .0053406 408.48 0.000 2.171105 2.19204

2023#girl#0 | 1.986592 .0059253 335.27 0.000 1.974978 1.998205

2023#girl#1 | 2.066093 .004854 425.65 0.000 2.05658 2.075607

------------------------------------------------------------------------------------

. margins year#schoollevel#gender#immigrant

Predictive margins Number of obs = 556,754

Model VCE: Robust

Expression: Predicted number of events, predict()

---------------------------------------------------------------------------------------------------------------------------

| Delta-method

| Margin std. err. z P>|z| [95% conf. interval]

----------------------------------------------------------+----------------------------------------------------------------

year#schoollevel#gender#immigrant |

2017 #|

lower secondary #|

boy #|

Student and parents born in Finland | 2.249542 .0063059 356.74 0.000 2.237183 2.261901

2017#lower secondary#boy#One foreign-born parent | 2.166402 .0227072 95.41 0.000 2.121897 2.210908

2017 #|

lower secondary #|

boy #|

Born in Finland, foreign-born parents | 2.096306 .0474381 44.19 0.000 2.003329 2.189283

2017#lower secondary#boy#Student and parents born abroad | 1.736903 .0353075 49.19 0.000 1.667701 1.806104

2017 #|

lower secondary #|

girl #|

Student and parents born in Finland | 2.234328 .0054585 409.33 0.000 2.22363 2.245027

2017#lower secondary#girl#One foreign-born parent | 2.14276 .0193384 110.80 0.000 2.104858 2.180663

2017 #|

lower secondary #|

girl #|

Born in Finland, foreign-born parents | 2.107615 .0387705 54.36 0.000 2.031626 2.183604

2017 #|

lower secondary #|

girl #|

Student and parents born abroad | 1.943738 .0361877 53.71 0.000 1.872811 2.014665

2017 #|

upper secondary #|

boy #|

Student and parents born in Finland | 2.242937 .0092003 243.79 0.000 2.224905 2.26097

2017#upper secondary#boy#One foreign-born parent | 2.16055 .0349952 61.74 0.000 2.091961 2.22914

2017 #|

upper secondary #|

boy #|

Born in Finland, foreign-born parents | 2.066753 .0732723 28.21 0.000 1.923142 2.210364

2017#upper secondary#boy#Student and parents born abroad | 1.938699 .0691847 28.02 0.000 1.803099 2.074298

2017 #|

upper secondary #|

girl #|

Student and parents born in Finland | 2.281248 .0069487 328.30 0.000 2.267629 2.294868

2017#upper secondary#girl#One foreign-born parent | 2.21704 .0271765 81.58 0.000 2.163775 2.270305

2017 #|

upper secondary #|

girl #|

Born in Finland, foreign-born parents | 2.167179 .0553357 39.16 0.000 2.058723 2.275635

2017 #|

upper secondary #|

girl #|

Student and parents born abroad | 2.071832 .050888 40.71 0.000 1.972093 2.17157

2017#vocational#boy#Student and parents born in Finland | 2.351183 .0089626 262.33 0.000 2.333617 2.36875

2017#vocational#boy#One foreign-born parent | 2.262759 .0383545 59.00 0.000 2.187586 2.337933

2017 #|

vocational #|

boy #|

Born in Finland, foreign-born parents | 2.365874 .0727974 32.50 0.000 2.223194 2.508555

2017#vocational#boy#Student and parents born abroad | 1.862535 .0588208 31.66 0.000 1.747248 1.977821

2017#vocational#girl#Student and parents born in Finland | 2.229207 .0100152 222.58 0.000 2.209578 2.248837

2017#vocational#girl#One foreign-born parent | 2.215351 .0373142 59.37 0.000 2.142216 2.288485

2017 #|

vocational #|

girl #|

Born in Finland, foreign-born parents | 1.939919 .0798562 24.29 0.000 1.783404 2.096434

2017#vocational#girl#Student and parents born abroad | 1.815283 .0626786 28.96 0.000 1.692435 1.938131

2019 #|

lower secondary #|

boy #|

Student and parents born in Finland | 2.237202 .0057461 389.34 0.000 2.225939 2.248464

2019#lower secondary#boy#One foreign-born parent | 2.128948 .0212383 100.24 0.000 2.087322 2.170574

2019 #|

lower secondary #|

boy #|

Born in Finland, foreign-born parents | 2.112445 .0406601 51.95 0.000 2.032753 2.192137

2019#lower secondary#boy#Student and parents born abroad | 1.75674 .032126 54.68 0.000 1.693775 1.819706

2019 #|

lower secondary #|

girl #|

Student and parents born in Finland | 2.200492 .0050456 436.12 0.000 2.190603 2.210381

2019#lower secondary#girl#One foreign-born parent | 2.136882 .017166 124.48 0.000 2.103237 2.170527

2019 #|

lower secondary #|

girl #|

Born in Finland, foreign-born parents | 2.032719 .0354898 57.28 0.000 1.96316 2.102278

2019 #|

lower secondary #|

girl #|

Student and parents born abroad | 1.845427 .0327719 56.31 0.000 1.781196 1.909659

2019 #|

upper secondary #|

boy #|

Student and parents born in Finland | 2.208698 .0084489 261.42 0.000 2.192138 2.225257

2019#upper secondary#boy#One foreign-born parent | 2.176723 .0292741 74.36 0.000 2.119347 2.234099

2019 #|

upper secondary #|

boy #|

Born in Finland, foreign-born parents | 2.06603 .0571791 36.13 0.000 1.953961 2.178099

2019#upper secondary#boy#Student and parents born abroad | 1.893114 .0534233 35.44 0.000 1.788407 1.997822

2019 #|

upper secondary #|

girl #|

Student and parents born in Finland | 2.230752 .0063122 353.40 0.000 2.21838 2.243123

2019#upper secondary#girl#One foreign-born parent | 2.18729 .0220139 99.36 0.000 2.144144 2.230437

2019 #|

upper secondary #|

girl #|

Born in Finland, foreign-born parents | 2.073465 .0455152 45.56 0.000 1.984257 2.162673

2019 #|

upper secondary #|

girl #|

Student and parents born abroad | 2.051559 .0404805 50.68 0.000 1.972219 2.130899

2019#vocational#boy#Student and parents born in Finland | 2.280816 .0097514 233.90 0.000 2.261704 2.299929

2019#vocational#boy#One foreign-born parent | 2.209243 .0381358 57.93 0.000 2.134498 2.283988

2019 #|

vocational #|

boy #|

Born in Finland, foreign-born parents | 2.187362 .0789841 27.69 0.000 2.032556 2.342168

2019#vocational#boy#Student and parents born abroad | 1.83832 .0588169 31.25 0.000 1.723041 1.953599

2019#vocational#girl#Student and parents born in Finland | 2.172225 .0107965 201.20 0.000 2.151064 2.193386

2019#vocational#girl#One foreign-born parent | 2.105041 .0398878 52.77 0.000 2.026862 2.183219

2019 #|

vocational #|

girl #|

Born in Finland, foreign-born parents | 1.907284 .0957407 19.92 0.000 1.719636 2.094933

2019#vocational#girl#Student and parents born abroad | 2.037334 .0611607 33.31 0.000 1.917462 2.157207

2021 #|

lower secondary #|

boy #|

Student and parents born in Finland | 2.218216 .0055729 398.03 0.000 2.207293 2.229139

2021#lower secondary#boy#One foreign-born parent | 2.058405 .0206398 99.73 0.000 2.017952 2.098858

2021 #|

lower secondary #|

boy #|

Born in Finland, foreign-born parents | 2.074634 .0364205 56.96 0.000 2.003251 2.146017

2021#lower secondary#boy#Student and parents born abroad | 1.697594 .0299328 56.71 0.000 1.638926 1.756261

2021 #|

lower secondary #|

girl #|

Student and parents born in Finland | 2.067338 .0049847 414.73 0.000 2.057568 2.077108

2021#lower secondary#girl#One foreign-born parent | 2.000863 .0168289 118.89 0.000 1.967879 2.033847

2021 #|

lower secondary #|

girl #|

Born in Finland, foreign-born parents | 1.938101 .0305609 63.42 0.000 1.878203 1.998

2021 #|

lower secondary #|

girl #|

Student and parents born abroad | 1.739543 .0295251 58.92 0.000 1.681675 1.797411

2021 #|

upper secondary #|

boy #|

Student and parents born in Finland | 2.171388 .008308 261.36 0.000 2.155105 2.187672

2021#upper secondary#boy#One foreign-born parent | 2.137496 .0284532 75.12 0.000 2.081729 2.193263

2021 #|

upper secondary #|

boy #|

Born in Finland, foreign-born parents | 2.05802 .0521144 39.49 0.000 1.955877 2.160162

2021#upper secondary#boy#Student and parents born abroad | 1.870879 .0492726 37.97 0.000 1.774307 1.967452

2021 #|

upper secondary #|

girl #|

Student and parents born in Finland | 2.139002 .0061792 346.16 0.000 2.126891 2.151113

2021#upper secondary#girl#One foreign-born parent | 2.053413 .0211239 97.21 0.000 2.012011 2.094815

2021 #|

upper secondary #|

girl #|

Born in Finland, foreign-born parents | 1.923777 .0434 44.33 0.000 1.838714 2.008839

2021 #|

upper secondary #|

girl #|

Student and parents born abroad | 1.896137 .0372207 50.94 0.000 1.823186 1.969088

2021#vocational#boy#Student and parents born in Finland | 2.26704 .0100055 226.58 0.000 2.24743 2.286651

2021#vocational#boy#One foreign-born parent | 2.138974 .0404315 52.90 0.000 2.05973 2.218219

2021 #|

vocational #|

boy #|

Born in Finland, foreign-born parents | 2.044914 .0854386 23.93 0.000 1.877457 2.21237

2021#vocational#boy#Student and parents born abroad | 1.904786 .058905 32.34 0.000 1.789334 2.020237

2021#vocational#girl#Student and parents born in Finland | 2.004367 .011223 178.59 0.000 1.98237 2.026364

2021#vocational#girl#One foreign-born parent | 1.979728 .0402642 49.17 0.000 1.900812 2.058645

2021 #|

vocational #|

girl #|

Born in Finland, foreign-born parents | 1.791814 .0993123 18.04 0.000 1.597165 1.986462

2021#vocational#girl#Student and parents born abroad | 1.808217 .0637536 28.36 0.000 1.683262 1.933172

2023 #|

lower secondary #|

boy #|

Student and parents born in Finland | 2.187073 .0058633 373.01 0.000 2.175581 2.198565

2023#lower secondary#boy#One foreign-born parent | 2.019786 .0207418 97.38 0.000 1.979133 2.06044

2023 #|

lower secondary #|

boy #|

Born in Finland, foreign-born parents | 2.01481 .0377001 53.44 0.000 1.940919 2.0887

2023#lower secondary#boy#Student and parents born abroad | 1.749636 .0285535 61.28 0.000 1.693672 1.8056

2023 #|

lower secondary #|

girl #|

Student and parents born in Finland | 2.013395 .0053548 376.00 0.000 2.0029 2.02389

2023#lower secondary#girl#One foreign-born parent | 1.949986 .0178339 109.34 0.000 1.915032 1.98494

2023 #|

lower secondary #|

girl #|

Born in Finland, foreign-born parents | 1.916299 .0325179 58.93 0.000 1.852565 1.980033

2023 #|

lower secondary #|

girl #|

Student and parents born abroad | 1.767764 .0282941 62.48 0.000 1.712309 1.82322

2023 #|

upper secondary #|

boy #|

Student and parents born in Finland | 2.162058 .0086783 249.13 0.000 2.145049 2.179067

2023#upper secondary#boy#One foreign-born parent | 2.039245 .0290593 70.18 0.000 1.98229 2.0962

2023 #|

upper secondary #|

boy #|

Born in Finland, foreign-born parents | 2.028508 .0534327 37.96 0.000 1.923782 2.133234

2023#upper secondary#boy#Student and parents born abroad | 1.886585 .0418765 45.05 0.000 1.804508 1.968661

2023 #|

upper secondary #|

girl #|

Student and parents born in Finland | 2.116728 .006749 313.64 0.000 2.1035 2.129955

2023#upper secondary#girl#One foreign-born parent | 2.095359 .0222006 94.38 0.000 2.051847 2.138871

2023 #|

upper secondary #|

girl #|

Born in Finland, foreign-born parents | 1.995527 .0405109 49.26 0.000 1.916127 2.074927

2023 #|

upper secondary #|

girl #|

Student and parents born abroad | 1.871826 .0341843 54.76 0.000 1.804826 1.938826

2023#vocational#boy#Student and parents born in Finland | 2.224176 .011066 200.99 0.000 2.202487 2.245865

2023#vocational#boy#One foreign-born parent | 2.143445 .0409679 52.32 0.000 2.06315 2.223741

2023 #|

vocational #|

boy #|

Born in Finland, foreign-born parents | 2.052066 .0810748 25.31 0.000 1.893162 2.21097

2023#vocational#boy#Student and parents born abroad | 1.829629 .056024 32.66 0.000 1.719824 1.939434

2023#vocational#girl#Student and parents born in Finland | 2.014392 .0121556 165.72 0.000 1.990567 2.038217

2023#vocational#girl#One foreign-born parent | 1.959394 .0417866 46.89 0.000 1.877494 2.041294

2023 #|

vocational #|

girl #|

Born in Finland, foreign-born parents | 2.124981 .0837465 25.37 0.000 1.960841 2.289121

2023#vocational#girl#Student and parents born abroad | 1.83332 .0621982 29.48 0.000 1.711414 1.955227

---------------------------------------------------------------------------------------------------------------------------

## 3.2. Loneliness

. glm loneliness i.year##i.gender##i.schoollevel i.degree i.immigrant i.urbanrural, family(gamma) link (log

> ) vce(robust) nolog

Generalized linear models Number of obs = 557,391

Optimization : ML Residual df = 557,361

Scale parameter = .1956516

Deviance = 113631.3463 (1/df) Deviance = .2038739

Pearson = 109048.5763 (1/df) Pearson = .1956516

Variance function: V(u) = u^2 [Gamma]

Link function : g(u) = ln(u) [Log]

AIC = 3.667397

Log pseudolikelihood = -1022056.921 BIC = -7260824

--------------------------------------------------------------------------------------------------------

| Robust

loneliness | Coefficient std. err. z P>|z| [95% conf. interval]

---------------------------------------+----------------------------------------------------------------

year |

2019 | .0032691 .0038917 0.84 0.401 -.0043585 .0108967

2021 | .1075286 .0038265 28.10 0.000 .1000288 .1150284

2023 | .0826386 .0039164 21.10 0.000 .0749627 .0903146

|

gender |

girl | .2543255 .0036906 68.91 0.000 .2470921 .261559

|

year#gender |

2019#girl | .0347235 .0049532 7.01 0.000 .0250154 .0444316

2021#girl | .0356527 .0048256 7.39 0.000 .0261947 .0451107

2023#girl | .0484103 .0049205 9.84 0.000 .0387663 .0580543

|

schoollevel |

upper secondary | .088675 .0049138 18.05 0.000 .0790442 .0983058

vocational | -.0015326 .0052103 -0.29 0.769 -.0117446 .0086795

|

year#schoollevel |

2019#upper secondary | .0221182 .0065128 3.40 0.001 .0093534 .034883

2019#vocational | .0046968 .0074214 0.63 0.527 -.0098488 .0192424

2021#upper secondary | .0150545 .0063799 2.36 0.018 .00255 .0275589

2021#vocational | .0280163 .007366 3.80 0.000 .0135792 .0424534

2023#upper secondary | .0077848 .0065235 1.19 0.233 -.0050011 .0205707

2023#vocational | .0212823 .0077607 2.74 0.006 .0060716 .036493

|

gender#schoollevel |

girl#upper secondary | -.0504675 .0060993 -8.27 0.000 -.0624218 -.0385132

girl#vocational | .0477446 .0069957 6.82 0.000 .0340332 .061456

|

year#gender#schoollevel |

2019#girl#upper secondary | -.02591 .00808 -3.21 0.001 -.0417465 -.0100734

2019#girl#vocational | -.0050362 .0099646 -0.51 0.613 -.0245665 .0144941

2021#girl#upper secondary | -.0272537 .0078606 -3.47 0.001 -.0426602 -.0118472

2021#girl#vocational | -.0268728 .0097611 -2.75 0.006 -.0460042 -.0077414

2023#girl#upper secondary | -.0358987 .0080364 -4.47 0.000 -.0516499 -.0201476

2023#girl#vocational | -.0203285 .0102141 -1.99 0.047 -.0403477 -.0003092

|

1.degree | -.0105942 .0012456 -8.51 0.000 -.0130356 -.0081529

|

immigrant |

One foreign-born parent | .0453626 .0022751 19.94 0.000 .0409035 .0498216

Born in Finland, foreign-born parents | -.0251473 .0047791 -5.26 0.000 -.0345143 -.0157804

Student and parents born abroad | .0960643 .0040575 23.68 0.000 .0881118 .1040169

|

urbanrural |

semi-urban | -.0125309 .0016277 -7.70 0.000 -.0157212 -.0093406

rural | -.0025161 .0018914 -1.33 0.183 -.0062231 .0011909

|

_cons | .6135261 .0030225 202.98 0.000 .6076021 .6194502

--------------------------------------------------------------------------------------------------------

. testparm year#gender#schoollevel

( 1) [loneliness]2019.year#2.gender#20.schoollevel = 0

( 2) [loneliness]2019.year#2.gender#30.schoollevel = 0

( 3) [loneliness]2021.year#2.gender#20.schoollevel = 0

( 4) [loneliness]2021.year#2.gender#30.schoollevel = 0

( 5) [loneliness]2023.year#2.gender#20.schoollevel = 0

( 6) [loneliness]2023.year#2.gender#30.schoollevel = 0

chi2( 6) = 27.08

Prob > chi2 = 0.0001

. margins year#gender#schoollevel

Predictive margins Number of obs = 557,391

Model VCE: Robust

Expression: Predicted mean loneliness, predict()

--------------------------------------------------------------------------------------------

| Delta-method

| Margin std. err. z P>|z| [95% conf. interval]

---------------------------+----------------------------------------------------------------

year#gender#schoollevel |

2017#boy#lower secondary | 1.843209 .005344 344.91 0.000 1.832734 1.853683

2017#boy#upper secondary | 2.014121 .0079891 252.11 0.000 1.998463 2.029779

2017#boy#vocational | 1.840386 .0079706 230.90 0.000 1.824764 1.856008

2017#girl#lower secondary | 2.376986 .005445 436.54 0.000 2.366314 2.387658

2017#girl#upper secondary | 2.469562 .0069333 356.19 0.000 2.455973 2.483151

2017#girl#vocational | 2.489409 .0102048 243.94 0.000 2.469408 2.50941

2019#boy#lower secondary | 1.849244 .0047986 385.37 0.000 1.839839 1.858649

2019#boy#upper secondary | 2.065909 .0070424 293.35 0.000 2.052106 2.079711

2019#boy#vocational | 1.855105 .0085759 216.32 0.000 1.838296 1.871913

2019#girl#lower secondary | 2.469031 .0050417 489.72 0.000 2.45915 2.478913

2019#girl#upper secondary | 2.555484 .0060623 421.54 0.000 2.543602 2.567366

2019#girl#vocational | 2.58493 .0110515 233.90 0.000 2.56327 2.606591

2021#boy#lower secondary | 2.052455 .0051189 400.95 0.000 2.042422 2.062488

2021#boy#upper secondary | 2.276789 .0073522 309.67 0.000 2.262378 2.291199

2021#boy#vocational | 2.107537 .0096684 217.98 0.000 2.088588 2.126487

2021#girl#lower secondary | 2.742897 .0050804 539.90 0.000 2.73294 2.752854

2021#girl#upper secondary | 2.815171 .0060304 466.83 0.000 2.803351 2.82699

2021#girl#vocational | 2.875913 .0114344 251.51 0.000 2.853502 2.898324

2023#boy#lower secondary | 2.002 .0052629 380.40 0.000 1.991685 2.012315

2023#boy#upper secondary | 2.204733 .0075088 293.62 0.000 2.190016 2.21945

2023#boy#vocational | 2.041932 .0104694 195.04 0.000 2.021412 2.062451

2023#girl#lower secondary | 2.70982 .0051878 522.35 0.000 2.699652 2.719988

2023#girl#upper secondary | 2.737311 .0063068 434.02 0.000 2.724949 2.749672

2023#girl#vocational | 2.840693 .0122573 231.76 0.000 2.816669 2.864717

--------------------------------------------------------------------------------------------

.

. glm loneliness i.year##i.schoollevel##i.gender##i.degree i.urbanrural i.immigrant, family(gamma) link (log

> ) vce(robust) nolog

Generalized linear models Number of obs = 557,391

Optimization : ML Residual df = 557,338

Scale parameter = .1956264

Deviance = 113607.8498 (1/df) Deviance = .2038401

Pearson = 109030.0473 (1/df) Pearson = .1956264

Variance function: V(u) = u^2 [Gamma]

Link function : g(u) = ln(u) [Log]

AIC = 3.667437

Log pseudolikelihood = -1022045.172 BIC = -7260544

--------------------------------------------------------------------------------------------------------

| Robust

loneliness | Coefficient std. err. z P>|z| [95% conf. interval]

---------------------------------------+----------------------------------------------------------------

year |

2019 | .0016592 .0056306 0.29 0.768 -.0093766 .012695

2021 | .1065879 .0056003 19.03 0.000 .0956115 .1175643

2023 | .0804142 .005795 13.88 0.000 .0690562 .0917722

|

schoollevel |

upper secondary | .1063925 .0080585 13.20 0.000 .0905982 .1221868

vocational | .0016828 .0067071 0.25 0.802 -.0114629 .0148285

|

year#schoollevel |

2019#upper secondary | .0221601 .0110453 2.01 0.045 .0005117 .0438084

2019#vocational | -.000657 .0096757 -0.07 0.946 -.019621 .018307

2021#upper secondary | .018654 .010982 1.70 0.089 -.0028704 .0401784

2021#vocational | .0216851 .0096859 2.24 0.025 .0027011 .040669

2023#upper secondary | .0144413 .0113859 1.27 0.205 -.0078747 .0367573

2023#vocational | .0103655 .0104145 1.00 0.320 -.0100465 .0307775

|

gender |

girl | .2582293 .0051938 49.72 0.000 .2480496 .268409

|

year#gender |

2019#girl | .0436451 .0071446 6.11 0.000 .0296418 .0576483

2021#girl | .0394077 .0070226 5.61 0.000 .0256436 .0531717

2023#girl | .0582074 .007244 8.04 0.000 .0440094 .0724054

|

schoollevel#gender |

upper secondary#girl | -.0610535 .0097396 -6.27 0.000 -.0801429 -.0419642

vocational#girl | .0467086 .0088811 5.26 0.000 .029302 .0641152

|

year#schoollevel#gender |

2019#upper secondary#girl | -.0308081 .0133062 -2.32 0.021 -.0568878 -.0047284

2019#vocational#girl | -.0118908 .0127948 -0.93 0.353 -.0369681 .0131865

2021#upper secondary#girl | -.0310544 .0131331 -2.36 0.018 -.0567948 -.005314

2021#vocational#girl | -.0300011 .012626 -2.38 0.017 -.0547477 -.0052546

2023#upper secondary#girl | -.0463724 .0136658 -3.39 0.001 -.0731568 -.019588

2023#vocational#girl | -.0213328 .0134986 -1.58 0.114 -.0477897 .005124

|

1.degree | .0001673 .0058016 0.03 0.977 -.0112037 .0115382

|

year#degree |

2019 1 | .0019926 .0077976 0.26 0.798 -.0132904 .0172755

2021 1 | .000456 .0076829 0.06 0.953 -.0146022 .0155142

2023 1 | .0023723 .0078824 0.30 0.763 -.013077 .0178216

|

schoollevel#degree |

upper secondary#1 | -.0295408 .0102478 -2.88 0.004 -.0496261 -.0094555

vocational#1 | -.0048172 .0108224 -0.45 0.656 -.0260287 .0163943

|

year#schoollevel#degree |

2019#upper secondary#1 | .0015598 .0137945 0.11 0.910 -.0254769 .0285965

2019#vocational#1 | .0155278 .0153414 1.01 0.311 -.0145407 .0455964

2021#upper secondary#1 | -.0021676 .0136155 -0.16 0.874 -.0288534 .0245182

2021#vocational#1 | .0176114 .0151878 1.16 0.246 -.0121561 .0473789

2023#upper secondary#1 | -.0059154 .0140229 -0.42 0.673 -.0333998 .021569

2023#vocational#1 | .0265313 .0158774 1.67 0.095 -.0045878 .0576504

|

gender#degree |

girl#1 | -.0078667 .0073842 -1.07 0.287 -.0223394 .0066061

|

year#gender#degree |

2019#girl#1 | -.0160304 .0099248 -1.62 0.106 -.0354826 .0034219

2021#girl#1 | -.0059318 .0096873 -0.61 0.540 -.0249186 .013055

2023#girl#1 | -.0159494 .0099035 -1.61 0.107 -.0353598 .0034611

|

schoollevel#gender#degree |

upper secondary#girl#1 | .0170589 .0125997 1.35 0.176 -.007636 .0417538

vocational#girl#1 | -.000855 .0147458 -0.06 0.954 -.0297562 .0280461

|

year#schoollevel#gender#degree |

2019#upper secondary#girl#1 | .0092948 .0168945 0.55 0.582 -.0238179 .0424074

2019#vocational#girl#1 | .0140168 .020858 0.67 0.502 -.0268642 .0548977

2021#upper secondary#girl#1 | .0048496 .0165506 0.29 0.770 -.027589 .0372882

2021#vocational#girl#1 | .0075399 .0203634 0.37 0.711 -.0323717 .0474514

2023#upper secondary#girl#1 | .0157413 .017066 0.92 0.356 -.0177075 .0491902

2023#vocational#girl#1 | -.001261 .0210847 -0.06 0.952 -.0425862 .0400642

|

urbanrural |

semi-urban | -.0126023 .001628 -7.74 0.000 -.015793 -.0094115

rural | -.0028433 .0018928 -1.50 0.133 -.0065531 .0008665

|

immigrant |

One foreign-born parent | .0452385 .0022749 19.89 0.000 .0407798 .0496972

Born in Finland, foreign-born parents | -.0257191 .0047823 -5.38 0.000 -.0350922 -.016346

Student and parents born abroad | .0958552 .0040608 23.61 0.000 .0878963 .1038142

|

_cons | .6083293 .0041426 146.85 0.000 .6002101 .6164486

--------------------------------------------------------------------------------------------------------

. testparm year#schoollevel#gender#degree

( 1) [loneliness]2019.year#20.schoollevel#2.gender#1.degree = 0

( 2) [loneliness]2019.year#30.schoollevel#2.gender#1.degree = 0

( 3) [loneliness]2021.year#20.schoollevel#2.gender#1.degree = 0

( 4) [loneliness]2021.year#30.schoollevel#2.gender#1.degree = 0

( 5) [loneliness]2023.year#20.schoollevel#2.gender#1.degree = 0

( 6) [loneliness]2023.year#30.schoollevel#2.gender#1.degree = 0

chi2( 6) = 1.77

Prob > chi2 = 0.9395

.

. glm loneliness i.year##i.schoollevel##i.gender##i.urbanrural i.degree i.immigrant, family(gamma) link (log

> ) vce(robust) nolog

Generalized linear models Number of obs = 557,391

Optimization : ML Residual df = 557,315

Scale parameter = .1956212

Deviance = 113599.5152 (1/df) Deviance = .2038336

Pearson = 109022.6028 (1/df) Pearson = .1956212

Variance function: V(u) = u^2 [Gamma]

Link function : g(u) = ln(u) [Log]

AIC = 3.667505

Log pseudolikelihood = -1022041.005 BIC = -7260248

--------------------------------------------------------------------------------------------------------

| Robust

loneliness | Coefficient std. err. z P>|z| [95% conf. interval]

---------------------------------------+----------------------------------------------------------------

year |

2019 | .003399 .0047991 0.71 0.479 -.0060071 .012805

2021 | .105284 .0047012 22.40 0.000 .0960699 .1144982

2023 | .0874999 .0048023 18.22 0.000 .0780875 .0969123

|

schoollevel |

upper secondary | .0884104 .0059001 14.98 0.000 .0768464 .0999745

vocational | .0032025 .0061222 0.52 0.601 -.0087969 .0152019

|

year#schoollevel |

2019#upper secondary | .0272987 .0077514 3.52 0.000 .0121062 .0424911

2019#vocational | .0050367 .0086805 0.58 0.562 -.0119768 .0220502

2021#upper secondary | .0182706 .007595 2.41 0.016 .0033847 .0331566

2021#vocational | .0383666 .0086098 4.46 0.000 .0214918 .0552415

2023#upper secondary | .0002724 .0077298 0.04 0.972 -.0148778 .0154226

2023#vocational | .0257501 .0091171 2.82 0.005 .007881 .0436192

|

gender |

girl | .2521906 .0045958 54.87 0.000 .243183 .2611982

|

year#gender |

2019#girl | .0327211 .0061083 5.36 0.000 .020749 .0446932

2021#girl | .0378511 .0059383 6.37 0.000 .0262124 .0494899

2023#girl | .042135 .0060382 6.98 0.000 .0303003 .0539697

|

schoollevel#gender |

upper secondary#girl | -.0503086 .0073319 -6.86 0.000 -.0646789 -.0359383

vocational#girl | .0370989 .0081495 4.55 0.000 .0211261 .0530717

|

year#schoollevel#gender |

2019#upper secondary#girl | -.0258755 .0096275 -2.69 0.007 -.0447451 -.007006

2019#vocational#girl | .0027884 .0115654 0.24 0.809 -.0198793 .0254562

2021#upper secondary#girl | -.0308237 .0093679 -3.29 0.001 -.0491845 -.012463

2021#vocational#girl | -.032712 .0113348 -2.89 0.004 -.0549277 -.0104963

2023#upper secondary#girl | -.0281516 .0095301 -2.95 0.003 -.0468303 -.009473

2023#vocational#girl | -.0205504 .0118868 -1.73 0.084 -.0438482 .0027474

|

urbanrural |

semi-urban | -.0076214 .0075461 -1.01 0.313 -.0224115 .0071687

rural | -.0108993 .0080336 -1.36 0.175 -.026645 .0048463

|

year#urbanrural |

2019#semi-urban | -.0049928 .0101921 -0.49 0.624 -.0249689 .0149833

2019#rural | .0045145 .0110092 0.41 0.682 -.0170631 .0260921

2021#semi-urban | .0095023 .0100801 0.94 0.346 -.0102543 .0292589

2021#rural | .003065 .0108786 0.28 0.778 -.0182567 .0243867

2023#semi-urban | -.0184421 .0103187 -1.79 0.074 -.0386663 .0017821

2023#rural | -.0124462 .0112084 -1.11 0.267 -.0344143 .0095219

|

schoollevel#urbanrural |

upper secondary#semi-urban | -.0115416 .013331 -0.87 0.387 -.0376698 .0145866

upper secondary#rural | .0160476 .0147373 1.09 0.276 -.0128369 .0449322

vocational#semi-urban | -.0271924 .013447 -2.02 0.043 -.0535481 -.0008367

vocational#rural | -.0139517 .021726 -0.64 0.521 -.0565338 .0286305

|

year#schoollevel#urbanrural |

2019#upper secondary#semi-urban | -.008136 .0177597 -0.46 0.647 -.0429443 .0266723

2019#upper secondary#rural | -.0370643 .0203952 -1.82 0.069 -.0770382 .0029096

2019#vocational#semi-urban | .0104872 .0192354 0.55 0.586 -.0272135 .0481878

2019#vocational#rural | -.031721 .0310274 -1.02 0.307 -.0925337 .0290917

2021#upper secondary#semi-urban | -.0108709 .0173904 -0.63 0.532 -.0449554 .0232136

2021#upper secondary#rural | -.0098824 .0198826 -0.50 0.619 -.0488516 .0290868

2021#vocational#semi-urban | -.0351912 .0190497 -1.85 0.065 -.072528 .0021456

2021#vocational#rural | -.0652839 .030772 -2.12 0.034 -.1255958 -.0049719

2023#upper secondary#semi-urban | .0318287 .0180812 1.76 0.078 -.0036097 .0672671

2023#upper secondary#rural | .0208339 .0204495 1.02 0.308 -.0192464 .0609142

2023#vocational#semi-urban | -.010754 .0198068 -0.54 0.587 -.0495746 .0280666

2023#vocational#rural | -.0245726 .0318571 -0.77 0.441 -.0870113 .0378661

|

gender#urbanrural |

girl#semi-urban | -.0008623 .0095873 -0.09 0.928 -.0196531 .0179284

girl#rural | .0143457 .0102517 1.40 0.162 -.0057472 .0344385

|

year#gender#urbanrural |

2019#girl#semi-urban | .011757 .012956 0.91 0.364 -.0136363 .0371503

2019#girl#rural | .0010214 .0140357 0.07 0.942 -.0264881 .0285309

2021#girl#semi-urban | -.0093083 .0126769 -0.73 0.463 -.0341546 .015538

2021#girl#rural | -.0020316 .013709 -0.15 0.882 -.0289008 .0248375

2023#girl#semi-urban | .0205701 .0129671 1.59 0.113 -.004845 .0459852

2023#girl#rural | .0217675 .0140676 1.55 0.122 -.0058046 .0493396

|

schoollevel#gender#urbanrural |

upper secondary#girl#semi-urban | .0067394 .0165469 0.41 0.684 -.025692 .0391708

upper secondary#girl#rural | -.0058899 .0182884 -0.32 0.747 -.0417345 .0299548

vocational#girl#semi-urban | .0631789 .0185991 3.40 0.001 .0267254 .0996324

vocational#girl#rural | .0430972 .0291942 1.48 0.140 -.0141225 .1003168

|

year#schoollevel#gender#urbanrural |

2019#upper secondary#girl#semi-urban | -.0044272 .0220735 -0.20 0.841 -.0476905 .038836

2019#upper secondary#girl#rural | .009909 .0250858 0.40 0.693 -.0392583 .0590764

2019#vocational#girl#semi-urban | -.0548514 .0266646 -2.06 0.040 -.1071131 -.0025896

2019#vocational#girl#rural | .0066554 .0406399 0.16 0.870 -.0729973 .0863082

2021#upper secondary#girl#semi-urban | .0251015 .0214722 1.17 0.242 -.0169833 .0671863

2021#upper secondary#girl#rural | -.0047738 .0243219 -0.20 0.844 -.0524438 .0428961

2021#vocational#girl#semi-urban | .0086251 .0259417 0.33 0.740 -.0422197 .0594698

2021#vocational#girl#rural | .0457034 .0400192 1.14 0.253 -.0327328 .1241397

2023#upper secondary#girl#semi-urban | -.0288896 .022297 -1.30 0.195 -.072591 .0148118

2023#upper secondary#girl#rural | -.0207158 .0250818 -0.83 0.409 -.0698751 .0284436

2023#vocational#girl#semi-urban | -.0076196 .026894 -0.28 0.777 -.0603309 .0450917

2023#vocational#girl#rural | -.0023865 .0418173 -0.06 0.954 -.084347 .079574

|

1.degree | -.0105434 .0012459 -8.46 0.000 -.0129854 -.0081014

|

immigrant |

One foreign-born parent | .0454022 .0022749 19.96 0.000 .0409434 .049861

Born in Finland, foreign-born parents | -.0248864 .0047772 -5.21 0.000 -.0342495 -.0155232

Student and parents born abroad | .0958914 .0040569 23.64 0.000 .08794 .1038429

|

_cons | .613929 .0036818 166.75 0.000 .6067127 .6211452

--------------------------------------------------------------------------------------------------------

. testparm year#schoollevel#gender#urbanrural

( 1) [loneliness]2019.year#20.schoollevel#2.gender#2.urbanrural = 0

( 2) [loneliness]2019.year#20.schoollevel#2.gender#3.urbanrural = 0

( 3) [loneliness]2019.year#30.schoollevel#2.gender#2.urbanrural = 0

( 4) [loneliness]2019.year#30.schoollevel#2.gender#3.urbanrural = 0

( 5) [loneliness]2021.year#20.schoollevel#2.gender#2.urbanrural = 0

( 6) [loneliness]2021.year#20.schoollevel#2.gender#3.urbanrural = 0

( 7) [loneliness]2021.year#30.schoollevel#2.gender#2.urbanrural = 0

( 8) [loneliness]2021.year#30.schoollevel#2.gender#3.urbanrural = 0

( 9) [loneliness]2023.year#20.schoollevel#2.gender#2.urbanrural = 0

(10) [loneliness]2023.year#20.schoollevel#2.gender#3.urbanrural = 0

(11) [loneliness]2023.year#30.schoollevel#2.gender#2.urbanrural = 0

(12) [loneliness]2023.year#30.schoollevel#2.gender#3.urbanrural = 0

chi2( 12) = 16.95

Prob > chi2 = 0.1514

.

. glm loneliness i.year##i.schoollevel##i.gender##i.immigrant i.degree i.urbanrural, family(gamma) link (log

> ) vce(robust) nolog

Generalized linear models Number of obs = 557,391

Optimization : ML Residual df = 557,292

Scale parameter = .1949982

Deviance = 113423.4618 (1/df) Deviance = .2035261

Pearson = 108670.9525 (1/df) Pearson = .1949982

Variance function: V(u) = u^2 [Gamma]

Link function : g(u) = ln(u) [Log]

AIC = 3.667271

Log pseudolikelihood = -1021952.978 BIC = -7260119

----------------------------------------------------------------------------------------------------------

| Robust

loneliness | Coefficient std. err. z P>|z| [95% conf. interval]

-----------------------------------------+----------------------------------------------------------------

year |

2019 | .0033821 .0040791 0.83 0.407 -.0046129 .011377

2021 | .1067268 .0040203 26.55 0.000 .0988472 .1146064

2023 | .0847282 .0041315 20.51 0.000 .0766306 .0928257

|

schoollevel |

upper secondary | .0967666 .0051467 18.80 0.000 .0866793 .106854

vocational | .0034211 .0054307 0.63 0.529 -.0072228 .014065

|

year#schoollevel |

2019#upper secondary | .0197006 .0068437 2.88 0.004 .0062873 .0331139

2019#vocational | .0051022 .0077585 0.66 0.511 -.0101041 .0203085

2021#upper secondary | .0138358 .0067108 2.06 0.039 .0006829 .0269886

2021#vocational | .0257108 .0077056 3.34 0.001 .0106081 .0408135

2023#upper secondary | .0040764 .006892 0.59 0.554 -.0094318 .0175845

2023#vocational | .0154162 .0081736 1.89 0.059 -.0006038 .0314362

|

gender |

girl | .2651798 .0038759 68.42 0.000 .2575832 .2727765

|

year#gender |

2019#girl | .033935 .0052087 6.52 0.000 .0237262 .0441439

2021#girl | .0365502 .0050848 7.19 0.000 .0265841 .0465163

2023#girl | .0502607 .0052002 9.67 0.000 .0400686 .0604528

|

schoollevel#gender |

upper secondary#girl | -.0580271 .0063926 -9.08 0.000 -.0705564 -.0454978

vocational#girl | .0473152 .0073065 6.48 0.000 .0329946 .0616357

|

year#schoollevel#gender |

2019#upper secondary#girl | -.021888 .0084968 -2.58 0.010 -.0385414 -.0052346

2019#vocational#girl | -.0028423 .0104263 -0.27 0.785 -.0232774 .0175928

2021#upper secondary#girl | -.0257742 .0082761 -3.11 0.002 -.041995 -.0095533

2021#vocational#girl | -.02311 .0102269 -2.26 0.024 -.0431543 -.0030658

2023#upper secondary#girl | -.032468 .008492 -3.82 0.000 -.0491119 -.0158241

2023#vocational#girl | -.0147469 .0107544 -1.37 0.170 -.0358251 .0063313

|

immigrant |

One foreign-born parent | .0574154 .0113615 5.05 0.000 .0351473 .0796835

Born in Finland, foreign-born parents | .02527 .0241983 1.04 0.296 -.0221578 .0726978

Student and parents born abroad | .2329567 .0179768 12.96 0.000 .1977228 .2681906

|

year#immigrant |

2019#One foreign-born parent | .004378 .015185 0.29 0.773 -.025384 .0341401

2019 #|

Born in Finland, foreign-born parents | .0249063 .0324599 0.77 0.443 -.0387139 .0885265

2019#Student and parents born abroad | -.0180104 .0240732 -0.75 0.454 -.065193 .0291721

2021#One foreign-born parent | .0243074 .0150089 1.62 0.105 -.0051096 .0537244

2021 #|

Born in Finland, foreign-born parents | -.0246945 .0306874 -0.80 0.421 -.0848408 .0354518

2021#Student and parents born abroad | -.0188865 .0229247 -0.82 0.410 -.0638181 .0260451

2023#One foreign-born parent | .0085563 .0150232 0.57 0.569 -.0208887 .0380014

2023 #|

Born in Finland, foreign-born parents | .0000291 .0314136 0.00 0.999 -.0615404 .0615986

2023#Student and parents born abroad | -.0778818 .0228756 -3.40 0.001 -.1227172 -.0330465

|

schoollevel#immigrant |

upper secondary#One foreign-born parent | -.0235743 .0198696 -1.19 0.235 -.0625179 .0153694

upper secondary #|

Born in Finland, foreign-born parents | -.0366629 .041893 -0.88 0.381 -.1187717 .0454458

upper secondary #|

Student and parents born abroad | -.1221417 .0368256 -3.32 0.001 -.1943186 -.0499648

vocational#One foreign-born parent | .0089444 .0228851 0.39 0.696 -.0359096 .0537984

vocational #|

Born in Finland, foreign-born parents | -.0516877 .0467109 -1.11 0.268 -.1432394 .0398641

vocational #|

Student and parents born abroad | -.0774266 .0344551 -2.25 0.025 -.1449574 -.0098959

|

year#schoollevel#immigrant |

2019 #|

upper secondary #|

One foreign-born parent | .0072711 .025763 0.28 0.778 -.0432234 .0577656

2019 #|

upper secondary #|

Born in Finland, foreign-born parents | .0174293 .0539251 0.32 0.747 -.0882619 .1231205

2019 #|

upper secondary #|

Student and parents born abroad | .0381545 .0468724 0.81 0.416 -.0537137 .1300227

2019#vocational#One foreign-born parent | -.0114445 .0315066 -0.36 0.716 -.0731962 .0503072

2019 #|

vocational #|

Born in Finland, foreign-born parents | -.0463307 .0659503 -0.70 0.482 -.1755908 .0829294

2019 #|

vocational #|

Student and parents born abroad | .014373 .0488188 0.29 0.768 -.0813101 .1100561

2021 #|

upper secondary #|

One foreign-born parent | .0033273 .0253246 0.13 0.895 -.0463079 .0529626

2021 #|

upper secondary #|

Born in Finland, foreign-born parents | .060389 .0524324 1.15 0.249 -.0423766 .1631546

2021 #|

upper secondary #|

Student and parents born abroad | -.0109868 .0448564 -0.24 0.807 -.0989037 .07693

2021#vocational#One foreign-born parent | .0195787 .0314917 0.62 0.534 -.0421439 .0813013

2021 #|

vocational #|

Born in Finland, foreign-born parents | .1140231 .0666778 1.71 0.087 -.016663 .2447093

2021 #|

vocational #|

Student and parents born abroad | -.0155624 .0472033 -0.33 0.742 -.1080792 .0769543

2023 #|

upper secondary #|

One foreign-born parent | .0215783 .0255445 0.84 0.398 -.0284879 .0716445

2023 #|

upper secondary #|

Born in Finland, foreign-born parents | .0076706 .052543 0.15 0.884 -.0953117 .110653

2023 #|

upper secondary #|

Student and parents born abroad | .0492284 .043768 1.12 0.261 -.0365553 .1350121

2023#vocational#One foreign-born parent | .0352602 .0319146 1.10 0.269 -.0272913 .0978117

2023 #|

vocational #|

Born in Finland, foreign-born parents | .044151 .0662901 0.67 0.505 -.0857753 .1740773

2023 #|

vocational #|

Student and parents born abroad | .0496634 .0467063 1.06 0.288 -.0418792 .141206

|

gender#immigrant |

girl#One foreign-born parent | -.0204148 .0143952 -1.42 0.156 -.0486288 .0077992

girl #|

Born in Finland, foreign-born parents | -.0885257 .0309384 -2.86 0.004 -.1491639 -.0278875

girl#Student and parents born abroad | -.1937252 .0244803 -7.91 0.000 -.2417058 -.1457447

|

year#gender#immigrant |

2019#girl#One foreign-born parent | .0069081 .0191084 0.36 0.718 -.0305437 .0443599

2019 #|

girl #|

Born in Finland, foreign-born parents | -.0396168 .0412725 -0.96 0.337 -.1205095 .0412758

2019 #|

girl #|

Student and parents born abroad | .0265666 .0326192 0.81 0.415 -.0373659 .090499

2021#girl#One foreign-born parent | -.030596 .0186995 -1.64 0.102 -.0672463 .0060543

2021 #|

girl #|

Born in Finland, foreign-born parents | .0510145 .0386859 1.32 0.187 -.0248084 .1268374

2021 #|

girl #|

Student and parents born abroad | .0241729 .0308179 0.78 0.433 -.036229 .0845749

2023#girl#One foreign-born parent | -.0307645 .0187892 -1.64 0.102 -.0675907 .0060617

2023 #|

girl #|

Born in Finland, foreign-born parents | .0163011 .0393754 0.41 0.679 -.0608734 .0934755

2023 #|

girl #|

Student and parents born abroad | .0206663 .0306055 0.68 0.500 -.0393194 .0806519

|

schoollevel#gender#immigrant |

upper secondary #|

girl #|

One foreign-born parent | .0251197 .0246402 1.02 0.308 -.0231741 .0734136

upper secondary #|

girl #|

Born in Finland, foreign-born parents | -.0314696 .0527501 -0.60 0.551 -.1348579 .0719187

upper secondary #|

girl #|

Student and parents born abroad | .1083979 .046545 2.33 0.020 .0171714 .1996244

vocational#girl#One foreign-born parent | -.0401132 .0299017 -1.34 0.180 -.0987195 .0184932

vocational #|

girl #|

Born in Finland, foreign-born parents | -.0029117 .0646516 -0.05 0.964 -.1296265 .1238031

vocational #|

girl #|

Student and parents born abroad | .0020622 .0479641 0.04 0.966 -.0919457 .0960701

|

year#schoollevel#gender#immigrant |

2019 #|

upper secondary #|

girl #|

One foreign-born parent | -.0342573 .0318839 -1.07 0.283 -.0967485 .0282339

2019 #|

upper secondary #|

girl #|

Born in Finland, foreign-born parents | .0878873 .0678342 1.30 0.195 -.0450653 .22084

2019 #|

upper secondary #|

girl #|

Student and parents born abroad | -.0765207 .0591761 -1.29 0.196 -.1925037 .0394623

2019 #|

vocational #|

girl #|

One foreign-born parent | -.0066439 .0413562 -0.16 0.872 -.0877005 .0744128

2019 #|

vocational #|

girl #|

Born in Finland, foreign-born parents | .116321 .0908965 1.28 0.201 -.0618328 .2944749

2019 #|

vocational #|

girl #|

Student and parents born abroad | -.1056401 .068997 -1.53 0.126 -.2408717 .0295914

2021 #|

upper secondary #|

girl #|

One foreign-born parent | -.0044163 .0310297 -0.14 0.887 -.0652333 .0564007

2021 #|

upper secondary #|

girl #|

Born in Finland, foreign-born parents | -.0067042 .06522 -0.10 0.918 -.134533 .1211246

2021 #|

upper secondary #|

girl #|

Student and parents born abroad | -.0077163 .0564608 -0.14 0.891 -.1183775 .102945

2021 #|

vocational #|

girl #|

One foreign-born parent | -.0077565 .0405358 -0.19 0.848 -.0872052 .0716921

2021 #|

vocational #|

girl #|

Born in Finland, foreign-born parents | -.1066841 .0906897 -1.18 0.239 -.2844326 .0710645

2021 #|

vocational #|

girl #|

Student and parents born abroad | -.0609965 .0649134 -0.94 0.347 -.1882244 .0662314

2023 #|

upper secondary #|

girl #|

One foreign-born parent | -.0220314 .0313907 -0.70 0.483 -.0835561 .0394932

2023 #|

upper secondary #|

girl #|

Born in Finland, foreign-born parents | .0490528 .0652526 0.75 0.452 -.0788399 .1769454

2023 #|

upper secondary #|

girl #|

Student and parents born abroad | -.0394769 .0551807 -0.72 0.474 -.147629 .0686753

2023 #|

vocational #|

girl #|

One foreign-born parent | -.0080228 .0410465 -0.20 0.845 -.0884724 .0724267

2023 #|

vocational #|

girl #|

Born in Finland, foreign-born parents | -.0842148 .0903786 -0.93 0.351 -.2613536 .092924

2023 #|

vocational #|

girl #|

Student and parents born abroad | -.0547282 .064972 -0.84 0.400 -.182071 .0726147

|

1.degree | -.0104541 .0012436 -8.41 0.000 -.0128914 -.0080167

|

urbanrural |

semi-urban | -.0127289 .0016247 -7.83 0.000 -.0159132 -.0095446

rural | -.0025119 .0018884 -1.33 0.183 -.006213 .0011892

|

_cons | .6055613 .0031512 192.17 0.000 .599385 .6117376

----------------------------------------------------------------------------------------------------------

. testparm year#schoollevel#gender#immigrant

( 1) [loneliness]2019.year#20.schoollevel#2.gender#2.immigrant = 0

( 2) [loneliness]2019.year#20.schoollevel#2.gender#3.immigrant = 0

( 3) [loneliness]2019.year#20.schoollevel#2.gender#4.immigrant = 0

( 4) [loneliness]2019.year#30.schoollevel#2.gender#2.immigrant = 0

( 5) [loneliness]2019.year#30.schoollevel#2.gender#3.immigrant = 0

( 6) [loneliness]2019.year#30.schoollevel#2.gender#4.immigrant = 0

( 7) [loneliness]2021.year#20.schoollevel#2.gender#2.immigrant = 0

( 8) [loneliness]2021.year#20.schoollevel#2.gender#3.immigrant = 0

( 9) [loneliness]2021.year#20.schoollevel#2.gender#4.immigrant = 0

(10) [loneliness]2021.year#30.schoollevel#2.gender#2.immigrant = 0

(11) [loneliness]2021.year#30.schoollevel#2.gender#3.immigrant = 0

(12) [loneliness]2021.year#30.schoollevel#2.gender#4.immigrant = 0

(13) [loneliness]2023.year#20.schoollevel#2.gender#2.immigrant = 0

(14) [loneliness]2023.year#20.schoollevel#2.gender#3.immigrant = 0

(15) [loneliness]2023.year#20.schoollevel#2.gender#4.immigrant = 0

(16) [loneliness]2023.year#30.schoollevel#2.gender#2.immigrant = 0

(17) [loneliness]2023.year#30.schoollevel#2.gender#3.immigrant = 0

(18) [loneliness]2023.year#30.schoollevel#2.gender#4.immigrant = 0

chi2( 18) = 15.84

Prob > chi2 = 0.6036

.

. glm loneliness i.year##i.schoollevel##degree i.year##i.gender##i.degree i.urbanrural i.immigrant, family(g

> amma) link (log) vce(robust) nolog

Generalized linear models Number of obs = 557,391

Optimization : ML Residual df = 557,354

Scale parameter = .195783

Deviance = 113808.1617 (1/df) Deviance = .2041937

Pearson = 109120.4499 (1/df) Pearson = .195783

Variance function: V(u) = u^2 [Gamma]

Link function : g(u) = ln(u) [Log]

AIC = 3.667739

Log pseudolikelihood = -1022145.328 BIC = -7260555

--------------------------------------------------------------------------------------------------------

| Robust

loneliness | Coefficient std. err. z P>|z| [95% conf. interval]

---------------------------------------+----------------------------------------------------------------

year |

2019 | .0078456 .0047902 1.64 0.101 -.001543 .0172341

2021 | .11471 .0047609 24.09 0.000 .1053789 .1240411

2023 | .0892673 .004952 18.03 0.000 .0795616 .098973

|

schoollevel |

upper secondary | .0683676 .0046196 14.80 0.000 .0593135 .0774218

vocational | .0220888 .0044972 4.91 0.000 .0132746 .0309031

|

year#schoollevel |

2019#upper secondary | .0028798 .0062906 0.46 0.647 -.0094495 .0152091

2019#vocational | -.0064173 .0064624 -0.99 0.321 -.0190834 .0062488

2021#upper secondary | -.0005584 .0061765 -0.09 0.928 -.012664 .0115473

2021#vocational | .0075687 .006391 1.18 0.236 -.0049575 .0200948

2023#upper secondary | -.0122334 .0064772 -1.89 0.059 -.0249284 .0004616

2023#vocational | .0005252 .0068297 0.08 0.939 -.0128607 .0139111

|

1.degree | .0053218 .0048923 1.09 0.277 -.0042669 .0149104

|

year#degree |

2019 1 | .0010669 .0065977 0.16 0.872 -.0118645 .0139982

2021 1 | .0000418 .006492 0.01 0.995 -.0126822 .0127658

2023 1 | .0022128 .0066782 0.33 0.740 -.0108762 .0153019

|

schoollevel#degree |

upper secondary#1 | -.0157664 .006063 -2.60 0.009 -.0276497 -.0038831

vocational#1 | -.0083816 .007488 -1.12 0.263 -.0230578 .0062946

|

year#schoollevel#degree |

2019#upper secondary#1 | .0089878 .0081051 1.11 0.267 -.006898 .0248735

2019#vocational#1 | .0200287 .0106108 1.89 0.059 -.000768 .0408255

2021#upper secondary#1 | .0021385 .0079033 0.27 0.787 -.0133518 .0176288

2021#vocational#1 | .0213761 .0103681 2.06 0.039 .001055 .0416971

2023#upper secondary#1 | .0035355 .0081843 0.43 0.666 -.0125055 .0195765

2023#vocational#1 | .0264244 .0107301 2.46 0.014 .0053937 .047455

|

gender |

girl | .2585664 .0037594 68.78 0.000 .2511981 .2659347

|

year#gender |

2019#girl | .031886 .0052449 6.08 0.000 .0216062 .0421659

2021#girl | .0239609 .0051696 4.63 0.000 .0138287 .034093

2023#girl | .0411106 .0053973 7.62 0.000 .030532 .0516892

|

gender#degree |

girl#1 | -.0178595 .005286 -3.38 0.001 -.0282199 -.0074991

|

year#gender#degree |

2019#girl#1 | -.0142742 .0071709 -1.99 0.047 -.0283289 -.0002195

2021#girl#1 | -.0048893 .0070086 -0.70 0.485 -.0186259 .0088472

2023#girl#1 | -.0150425 .0072219 -2.08 0.037 -.029197 -.0008879

|

urbanrural |

semi-urban | -.0128519 .001628 -7.89 0.000 -.0160427 -.0096612

rural | -.0026998 .0018945 -1.43 0.154 -.006413 .0010134

|

immigrant |

One foreign-born parent | .0454583 .0022746 19.98 0.000 .0410001 .0499164

Born in Finland, foreign-born parents | -.0254359 .0047838 -5.32 0.000 -.0348119 -.0160599

Student and parents born abroad | .0949075 .0040537 23.41 0.000 .0869624 .1028527

|

_cons | .6081992 .0035186 172.85 0.000 .6013029 .6150954

--------------------------------------------------------------------------------------------------------

. testparm year#schoollevel#degree

( 1) [loneliness]2019.year#20.schoollevel#1.degree = 0

( 2) [loneliness]2019.year#30.schoollevel#1.degree = 0

( 3) [loneliness]2021.year#20.schoollevel#1.degree = 0

( 4) [loneliness]2021.year#30.schoollevel#1.degree = 0

( 5) [loneliness]2023.year#20.schoollevel#1.degree = 0

( 6) [loneliness]2023.year#30.schoollevel#1.degree = 0

chi2( 6) = 8.37

Prob > chi2 = 0.2120

. testparm year#gender#degree

( 1) [loneliness]2019.year#2.gender#1.degree = 0

( 2) [loneliness]2021.year#2.gender#1.degree = 0

( 3) [loneliness]2023.year#2.gender#1.degree = 0

chi2( 3) = 6.38

Prob > chi2 = 0.0946

. glm loneliness i.year##i.degree i.urbanrural i.immigrant i.schoollevel i.gender, family(gamma) link (log)

> vce(robust) nolog

Generalized linear models Number of obs = 557,391

Optimization : ML Residual df = 557,375

Scale parameter = .1958253

Deviance = 113867.941 (1/df) Deviance = .2042932

Pearson = 109148.1088 (1/df) Pearson = .1958253

Variance function: V(u) = u^2 [Gamma]

Link function : g(u) = ln(u) [Log]

AIC = 3.667771

Log pseudolikelihood = -1022175.218 BIC = -7260773

--------------------------------------------------------------------------------------------------------

| Robust

loneliness | Coefficient std. err. z P>|z| [95% conf. interval]

---------------------------------------+----------------------------------------------------------------

year |

2019 | .0244076 .0025709 9.49 0.000 .0193687 .0294464

2021 | .129135 .0025317 51.01 0.000 .124173 .1340969

2023 | .1088521 .00265 41.08 0.000 .1036582 .114046

|

1.degree | -.0083713 .002634 -3.18 0.001 -.0135337 -.0032088

|

year#degree |

2019 1 | -.0006191 .0035321 -0.18 0.861 -.0075419 .0063037

2021 1 | -.0013275 .0034458 -0.39 0.700 -.0080811 .0054262

2023 1 | -.0046067 .0035535 -1.30 0.195 -.0115714 .002358

|

urbanrural |

semi-urban | -.0127615 .0016276 -7.84 0.000 -.0159515 -.0095714

rural | -.0023777 .0018927 -1.26 0.209 -.0060873 .0013319

|

immigrant |

One foreign-born parent | .0455681 .0022746 20.03 0.000 .04111 .0500263

Born in Finland, foreign-born parents | -.0250579 .0047796 -5.24 0.000 -.0344257 -.01569

Student and parents born abroad | .0946752 .0040488 23.38 0.000 .0867396 .1026108

|

schoollevel |

upper secondary | .0581269 .0013095 44.39 0.000 .0555603 .0606936

vocational | .0252017 .0018258 13.80 0.000 .0216233 .0287801

|

gender |

girl | .2686824 .0012056 222.85 0.000 .2663194 .2710455

_cons | .6040957 .0021743 277.83 0.000 .599834 .6083573

--------------------------------------------------------------------------------------------------------

. testparm year#degree

( 1) [loneliness]2019.year#1.degree = 0

( 2) [loneliness]2021.year#1.degree = 0

( 3) [loneliness]2023.year#1.degree = 0

chi2( 3) = 2.11

Prob > chi2 = 0.5491

. glm loneliness i.year##i.schoollevel##i.urbanrural i.year##i.gender##i.urbanrural i.immigrant i.degree, fa

> mily(gamma) link (log) vce(robust) nolog

Generalized linear models Number of obs = 557,391

Optimization : ML Residual df = 557,339

Scale parameter = .1957915

Deviance = 113819.6486 (1/df) Deviance = .2042198

Pearson = 109122.249 (1/df) Pearson = .1957915

Variance function: V(u) = u^2 [Gamma]

Link function : g(u) = ln(u) [Log]

AIC = 3.667813

Log pseudolikelihood = -1022151.072 BIC = -7260345

--------------------------------------------------------------------------------------------------------

| Robust

loneliness | Coefficient std. err. z P>|z| [95% conf. interval]

---------------------------------------+----------------------------------------------------------------

year |

2019 | .0092271 .0039966 2.31 0.021 .001394 .0170603

2021 | .1152493 .0039101 29.47 0.000 .1075856 .1229129

2023 | .0966542 .0039969 24.18 0.000 .0888205 .1044879

|

schoollevel |

upper secondary | .0592894 .0035665 16.62 0.000 .0522992 .0662797

vocational | .0188468 .0041287 4.56 0.000 .0107546 .0269389

|

year#schoollevel |

2019#upper secondary | .0126272 .0046792 2.70 0.007 .0034562 .0217982

2019#vocational | .0053223 .0058518 0.91 0.363 -.006147 .0167917

2021#upper secondary | .000654 .004537 0.14 0.885 -.0082385 .0095464

2021#vocational | .0225718 .0057449 3.93 0.000 .011312 .0338315

2023#upper secondary | -.0152906 .0046204 -3.31 0.001 -.0243464 -.0062349

2023#vocational | .016256 .0060071 2.71 0.007 .0044822 .0280298

|

urbanrural |

semi-urban | -.0140845 .0064248 -2.19 0.028 -.0266769 -.001492

rural | -.0099306 .0070682 -1.40 0.160 -.0237841 .0039228

|

year#urbanrural |

2019#semi-urban | -.0008096 .0087054 -0.09 0.926 -.0178719 .0162527

2019#rural | .0010028 .0096934 0.10 0.918 -.017996 .0200015

2021#semi-urban | .0043688 .0085774 0.51 0.611 -.0124425 .0211801

2021#rural | -.0021088 .0095618 -0.22 0.825 -.0208497 .016632

2023#semi-urban | -.0163513 .0088137 -1.86 0.064 -.0336258 .0009232

2023#rural | -.0139003 .0098631 -1.41 0.159 -.0332316 .005431

|

schoollevel#urbanrural |

upper secondary#semi-urban | -.0080542 .0080505 -1.00 0.317 -.023833 .0077245

upper secondary#rural | .0127193 .008881 1.43 0.152 -.0046871 .0301257

vocational#semi-urban | -.005554 .0094998 -0.58 0.559 -.0241732 .0130653

vocational#rural | .0068533 .0147541 0.46 0.642 -.0220643 .0357708

|

year#schoollevel#urbanrural |

2019#upper secondary#semi-urban | -.0098841 .0107475 -0.92 0.358 -.0309489 .0111806

2019#upper secondary#rural | -.0321763 .0121277 -2.65 0.008 -.0559461 -.0084064

2019#vocational#semi-urban | -.0119105 .0136318 -0.87 0.382 -.0386283 .0148073

2019#vocational#rural | -.0260175 .0205218 -1.27 0.205 -.0662395 .0142046

2021#upper secondary#semi-urban | .0041777 .010429 0.40 0.689 -.0162627 .024618

2021#upper secondary#rural | -.0134798 .0117373 -1.15 0.251 -.0364845 .0095249

2021#vocational#semi-urban | -.0281608 .0132973 -2.12 0.034 -.054223 -.0020986

2021#vocational#rural | -.0397747 .0201423 -1.97 0.048 -.0792528 -.0002966

2023#upper secondary#semi-urban | .0162827 .0108519 1.50 0.133 -.0049867 .0375521

2023#upper secondary#rural | .0099552 .0121763 0.82 0.414 -.01391 .0338204

2023#vocational#semi-urban | -.0145246 .0138222 -1.05 0.293 -.0416155 .0125664

2023#vocational#rural | -.0274462 .0212512 -1.29 0.197 -.0690977 .0142054

|

gender |

girl | .2461956 .0031697 77.67 0.000 .239983 .2524081

|

year#gender |

2019#girl | .0215668 .004255 5.07 0.000 .0132271 .0299065

2021#girl | .0188959 .0041422 4.56 0.000 .0107774 .0270145

2023#girl | .0247594 .0042327 5.85 0.000 .0164636 .0330553

|

gender#urbanrural |

girl#semi-urban | .0116195 .0070241 1.65 0.098 -.0021475 .0253865

girl#rural | .0124926 .0080402 1.55 0.120 -.003266 .0282512

|

year#gender#urbanrural |

2019#girl#semi-urban | .0036319 .0095597 0.38 0.704 -.0151047 .0223686

2019#girl#rural | .0077458 .011053 0.70 0.483 -.0139176 .0294093

2021#girl#semi-urban | .0003526 .0093245 0.04 0.970 -.0179232 .0186283

2021#girl#rural | .0077265 .010784 0.72 0.474 -.0134099 .0288628

2023#girl#semi-urban | .0161782 .0096212 1.68 0.093 -.0026791 .0350355

2023#girl#rural | .0239684 .0111066 2.16 0.031 .0021999 .045737

|

immigrant |

One foreign-born parent | .0456054 .0022747 20.05 0.000 .0411471 .0500637

Born in Finland, foreign-born parents | -.0246059 .0047784 -5.15 0.000 -.0339714 -.0152403

Student and parents born abroad | .0946924 .0040481 23.39 0.000 .0867583 .1026265

|

1.degree | -.0099587 .0012459 -7.99 0.000 -.0124007 -.0075168

_cons | .6167951 .0030849 199.94 0.000 .6107489 .6228414

--------------------------------------------------------------------------------------------------------

. testparm year#schoollevel#urbanrural

( 1) [loneliness]2019.year#20.schoollevel#2.urbanrural = 0

( 2) [loneliness]2019.year#20.schoollevel#3.urbanrural = 0

( 3) [loneliness]2019.year#30.schoollevel#2.urbanrural = 0

( 4) [loneliness]2019.year#30.schoollevel#3.urbanrural = 0

( 5) [loneliness]2021.year#20.schoollevel#2.urbanrural = 0

( 6) [loneliness]2021.year#20.schoollevel#3.urbanrural = 0

( 7) [loneliness]2021.year#30.schoollevel#2.urbanrural = 0

( 8) [loneliness]2021.year#30.schoollevel#3.urbanrural = 0

( 9) [loneliness]2023.year#20.schoollevel#2.urbanrural = 0

(10) [loneliness]2023.year#20.schoollevel#3.urbanrural = 0

(11) [loneliness]2023.year#30.schoollevel#2.urbanrural = 0

(12) [loneliness]2023.year#30.schoollevel#3.urbanrural = 0

chi2( 12) = 28.06

Prob > chi2 = 0.0054

. testparm year#gender#urbanrural

( 1) [loneliness]2019.year#2.gender#2.urbanrural = 0

( 2) [loneliness]2019.year#2.gender#3.urbanrural = 0

( 3) [loneliness]2021.year#2.gender#2.urbanrural = 0

( 4) [loneliness]2021.year#2.gender#3.urbanrural = 0

( 5) [loneliness]2023.year#2.gender#2.urbanrural = 0

( 6) [loneliness]2023.year#2.gender#3.urbanrural = 0

chi2( 6) = 7.90

Prob > chi2 = 0.2455

. glm loneliness i.year##i.schoollevel##i.immigrant i.year##i.gender##i.immigrant i.urbanrural i.degree, fam

> ily(gamma) link (log) vce(robust) nolog

Generalized linear models Number of obs = 557,391

Optimization : ML Residual df = 557,324

Scale parameter = .195197

Deviance = 113654.1461 (1/df) Deviance = .2039283

Pearson = 108787.9642 (1/df) Pearson = .195197

Variance function: V(u) = u^2 [Gamma]

Link function : g(u) = ln(u) [Log]

AIC = 3.66757

Log pseudolikelihood = -1022068.321 BIC = -7260312

----------------------------------------------------------------------------------------------------------

| Robust

loneliness | Coefficient std. err. z P>|z| [95% conf. interval]

-----------------------------------------+----------------------------------------------------------------

year |

2019 | .0087919 .0034456 2.55 0.011 .0020387 .0155451

2021 | .1147389 .0033877 33.87 0.000 .1080991 .1213787

2023 | .0935318 .003486 26.83 0.000 .0866994 .1003642

|

schoollevel |

upper secondary | .0631877 .0031139 20.29 0.000 .0570846 .0692909

vocational | .0226866 .0037195 6.10 0.000 .0153965 .0299767

|

year#schoollevel |

2019#upper secondary | .0073462 .0041301 1.78 0.075 -.0007487 .0154411

2019#vocational | .0031933 .0052921 0.60 0.546 -.007179 .0135655

2021#upper secondary | -.0008538 .0040107 -0.21 0.831 -.0087147 .0070071

2021#vocational | .0148686 .0051994 2.86 0.004 .0046779 .0250592

2023#upper secondary | -.0138297 .0041203 -3.36 0.001 -.0219054 -.005754

2023#vocational | .0086939 .0054643 1.59 0.112 -.0020159 .0194038

|

immigrant |

One foreign-born parent | .0577714 .0097106 5.95 0.000 .038739 .0768039

Born in Finland, foreign-born parents | .0305562 .0206749 1.48 0.139 -.0099659 .0710782

Student and parents born abroad | .2215752 .0159991 13.85 0.000 .1902176 .2529329

|

year#immigrant |

2019#One foreign-born parent | .0102107 .0129092 0.79 0.429 -.0150909 .0355123

2019 #|

Born in Finland, foreign-born parents | .0052872 .0276538 0.19 0.848 -.0489133 .0594877

2019#Student and parents born abroad | -.0064816 .0213473 -0.30 0.761 -.0483215 .0353584

2021#One foreign-born parent | .0245442 .0127228 1.93 0.054 -.0003921 .0494805

2021 #|

Born in Finland, foreign-born parents | -.017942 .0262812 -0.68 0.495 -.0694522 .0335683

2021#Student and parents born abroad | -.018884 .0203308 -0.93 0.353 -.0587316 .0209637

2023#One foreign-born parent | .0112507 .0127777 0.88 0.379 -.0137932 .0362946

2023 #|

Born in Finland, foreign-born parents | -.0019584 .0266993 -0.07 0.942 -.0542881 .0503713

2023#Student and parents born abroad | -.0750329 .020194 -3.72 0.000 -.1146124 -.0354534

|

schoollevel#immigrant |

upper secondary#One foreign-born parent | -.0087721 .0119492 -0.73 0.463 -.0321921 .0146478

upper secondary #|

Born in Finland, foreign-born parents | -.0551037 .0257001 -2.14 0.032 -.105475 -.0047324

upper secondary #|

Student and parents born abroad | -.0631575 .022958 -2.75 0.006 -.1081545 -.0181606

vocational#One foreign-born parent | -.0073852 .0150466 -0.49 0.624 -.036876 .0221057

vocational #|

Born in Finland, foreign-born parents | -.0511149 .0323464 -1.58 0.114 -.1145127 .012283

vocational #|

Student and parents born abroad | -.0767929 .0245938 -3.12 0.002 -.1249959 -.02859

|

year#schoollevel#immigrant |

2019 #|

upper secondary #|

One foreign-born parent | -.0132093 .0154376 -0.86 0.392 -.0434664 .0170479

2019 #|

upper secondary #|

Born in Finland, foreign-born parents | .0675229 .0330271 2.04 0.041 .002791 .1322547

2019 #|

upper secondary #|

Student and parents born abroad | -.0000774 .0291346 -0.00 0.998 -.0571803 .0570254

2019#vocational#One foreign-born parent | -.016869 .0209002 -0.81 0.420 -.0578325 .0240946

2019 #|

vocational #|

Born in Finland, foreign-born parents | .0004375 .0459607 0.01 0.992 -.0896438 .0905188

2019 #|

vocational #|

Student and parents born abroad | -.0274761 .0352103 -0.78 0.435 -.0964871 .0415349

2021 #|

upper secondary #|

One foreign-born parent | -.0007359 .0149145 -0.05 0.961 -.0299677 .0284959

2021 #|

upper secondary #|

Born in Finland, foreign-born parents | .0579282 .0316785 1.83 0.067 -.0041604 .1200169

2021 #|

upper secondary #|

Student and parents born abroad | -.011916 .0277787 -0.43 0.668 -.0663612 .0425293

2021#vocational#One foreign-born parent | .0146367 .0204454 0.72 0.474 -.0254355 .0547088

2021 #|

vocational #|

Born in Finland, foreign-born parents | .0640133 .0459967 1.39 0.164 -.0261387 .1541652

2021 #|

vocational #|

Student and parents born abroad | -.038736 .0333745 -1.16 0.246 -.1041488 .0266768

2023 #|

upper secondary #|

One foreign-born parent | .0082211 .015173 0.54 0.588 -.0215173 .0379596

2023 #|

upper secondary #|

Born in Finland, foreign-born parents | .0352348 .0315688 1.12 0.264 -.0266389 .0971085

2023 #|

upper secondary #|

Student and parents born abroad | .0306801 .027254 1.13 0.260 -.0227368 .084097

2023#vocational#One foreign-born parent | .0297077 .0207076 1.43 0.151 -.0108783 .0702938

2023 #|

vocational #|

Born in Finland, foreign-born parents | .0039376 .0454443 0.09 0.931 -.0851316 .0930068

2023 #|

vocational #|

Student and parents born abroad | .0284724 .0334165 0.85 0.394 -.0370227 .0939676

|

gender |

girl | .2585217 .0027656 93.48 0.000 .2531012 .2639423

|

year#gender |

2019#girl | .0235097 .0037458 6.28 0.000 .016168 .0308514

2021#girl | .0213027 .0036548 5.83 0.000 .0141394 .0284659

2023#girl | .0334421 .0037602 8.89 0.000 .0260723 .0408119

|

gender#immigrant |

girl#One foreign-born parent | -.0210248 .010694 -1.97 0.049 -.0419846 -.000065

girl #|

Born in Finland, foreign-born parents | -.0978267 .0229799 -4.26 0.000 -.1428663 -.052787

girl#Student and parents born abroad | -.1681602 .0186677 -9.01 0.000 -.2047482 -.1315723

|

year#gender#immigrant |

2019#girl#One foreign-born parent | -.0024523 .0141258 -0.17 0.862 -.0301382 .0252337

2019 #|

girl #|

Born in Finland, foreign-born parents | -.0033122 .0304203 -0.11 0.913 -.0629349 .0563105

2019 #|

girl #|

Student and parents born abroad | -.0024954 .0248075 -0.10 0.920 -.0511172 .0461263

2021#girl#One foreign-born parent | -.0300495 .0137885 -2.18 0.029 -.0570744 -.0030246

2021 #|

girl #|

Born in Finland, foreign-born parents | .0394534 .0290333 1.36 0.174 -.0174508 .0963577

2021 #|

girl #|

Student and parents born abroad | .0202241 .0234787 0.86 0.389 -.0257932 .0662415

2023#girl#One foreign-born parent | -.0352159 .0139155 -2.53 0.011 -.0624897 -.007942

2023 #|

girl #|

Born in Finland, foreign-born parents | .020337 .029195 0.70 0.486 -.0368842 .0775581

2023 #|

girl #|

Student and parents born abroad | .0098798 .0231135 0.43 0.669 -.0354219 .0551814

|

urbanrural |

semi-urban | -.0130294 .0016248 -8.02 0.000 -.016214 -.0098448

rural | -.0024443 .00189 -1.29 0.196 -.0061485 .00126

|

1.degree | -.009831 .0012435 -7.91 0.000 -.0122683 -.0073937

_cons | .6088106 .0026965 225.78 0.000 .6035255 .6140956

----------------------------------------------------------------------------------------------------------

. testparm year#schoollevel#immigrant

( 1) [loneliness]2019.year#20.schoollevel#2.immigrant = 0

( 2) [loneliness]2019.year#20.schoollevel#3.immigrant = 0

( 3) [loneliness]2019.year#20.schoollevel#4.immigrant = 0

( 4) [loneliness]2019.year#30.schoollevel#2.immigrant = 0

( 5) [loneliness]2019.year#30.schoollevel#3.immigrant = 0

( 6) [loneliness]2019.year#30.schoollevel#4.immigrant = 0

( 7) [loneliness]2021.year#20.schoollevel#2.immigrant = 0

( 8) [loneliness]2021.year#20.schoollevel#3.immigrant = 0

( 9) [loneliness]2021.year#20.schoollevel#4.immigrant = 0

(10) [loneliness]2021.year#30.schoollevel#2.immigrant = 0

(11) [loneliness]2021.year#30.schoollevel#3.immigrant = 0

(12) [loneliness]2021.year#30.schoollevel#4.immigrant = 0

(13) [loneliness]2023.year#20.schoollevel#2.immigrant = 0

(14) [loneliness]2023.year#20.schoollevel#3.immigrant = 0

(15) [loneliness]2023.year#20.schoollevel#4.immigrant = 0

(16) [loneliness]2023.year#30.schoollevel#2.immigrant = 0

(17) [loneliness]2023.year#30.schoollevel#3.immigrant = 0

(18) [loneliness]2023.year#30.schoollevel#4.immigrant = 0

chi2( 18) = 21.94

Prob > chi2 = 0.2345

. testparm year#gender#immigrant

( 1) [loneliness]2019.year#2.gender#2.immigrant = 0

( 2) [loneliness]2019.year#2.gender#3.immigrant = 0

( 3) [loneliness]2019.year#2.gender#4.immigrant = 0

( 4) [loneliness]2021.year#2.gender#2.immigrant = 0

( 5) [loneliness]2021.year#2.gender#3.immigrant = 0

( 6) [loneliness]2021.year#2.gender#4.immigrant = 0

( 7) [loneliness]2023.year#2.gender#2.immigrant = 0

( 8) [loneliness]2023.year#2.gender#3.immigrant = 0

( 9) [loneliness]2023.year#2.gender#4.immigrant = 0

chi2( 9) = 16.46

Prob > chi2 = 0.0580

. glm loneliness i.year##i.immigrant i.schoollevel i.gender i.urbanrural i.degree, family(gamma) link (log)

> vce(robust) nolog

Generalized linear models Number of obs = 557,391

Optimization : ML Residual df = 557,369

Scale parameter = .1957751

Deviance = 113855.4079 (1/df) Deviance = .2042729

Pearson = 109118.9787 (1/df) Pearson = .1957751

Variance function: V(u) = u^2 [Gamma]

Link function : g(u) = ln(u) [Log]

AIC = 3.66777

Log pseudolikelihood = -1022168.952 BIC = -7260706

--------------------------------------------------------------------------------------------------------

| Robust

loneliness | Coefficient std. err. z P>|z| [95% conf. interval]

---------------------------------------+----------------------------------------------------------------

year |

2019 | .0241178 .0018491 13.04 0.000 .0204936 .0277419

2021 | .1281044 .001803 71.05 0.000 .1245707 .1316382

2023 | .1084771 .0018568 58.42 0.000 .1048379 .1121163

|

immigrant |

One foreign-born parent | .0430243 .005275 8.16 0.000 .0326855 .0533631

Born in Finland, foreign-born parents | -.0448166 .0114401 -3.92 0.000 -.0672387 -.0223945

Student and parents born abroad | .1259616 .0098715 12.76 0.000 .1066138 .1453094

|

year#immigrant |

2019#One foreign-born parent | .0025067 .0069352 0.36 0.718 -.0110861 .0160995

2019 #|

Born in Finland, foreign-born parents | .0254997 .0151122 1.69 0.092 -.0041196 .055119

2019#Student and parents born abroad | -.0198934 .0130101 -1.53 0.126 -.0453927 .0056058

2021#One foreign-born parent | .0086846 .0067497 1.29 0.198 -.0045446 .0219137

2021 #|

Born in Finland, foreign-born parents | .0273077 .0143629 1.90 0.057 -.000843 .0554584

2021#Student and parents born abroad | -.0250677 .0123656 -2.03 0.043 -.0493037 -.0008316

2023#One foreign-born parent | -.0019563 .0068375 -0.29 0.775 -.0153576 .0114449

2023 #|

Born in Finland, foreign-born parents | .0198758 .014421 1.38 0.168 -.0083887 .0481404

2023#Student and parents born abroad | -.0672943 .0121775 -5.53 0.000 -.0911617 -.0434269

|

schoollevel |

upper secondary | .0581967 .0013095 44.44 0.000 .0556302 .0607632

vocational | .0252277 .0018252 13.82 0.000 .0216503 .0288051

|

gender |

girl | .2686781 .0012055 222.87 0.000 .2663153 .2710409

|

urbanrural |

semi-urban | -.0127451 .0016273 -7.83 0.000 -.0159345 -.0095557

rural | -.0023423 .0018922 -1.24 0.216 -.006051 .0013664

|

1.degree | -.0099622 .0012454 -8.00 0.000 -.0124032 -.0075212

_cons | .604417 .0018702 323.18 0.000 .6007514 .6080826

--------------------------------------------------------------------------------------------------------

. testparm year#immigrant

( 1) [loneliness]2019.year#2.immigrant = 0

( 2) [loneliness]2019.year#3.immigrant = 0

( 3) [loneliness]2019.year#4.immigrant = 0

( 4) [loneliness]2021.year#2.immigrant = 0

( 5) [loneliness]2021.year#3.immigrant = 0

( 6) [loneliness]2021.year#4.immigrant = 0

( 7) [loneliness]2023.year#2.immigrant = 0

( 8) [loneliness]2023.year#3.immigrant = 0

( 9) [loneliness]2023.year#4.immigrant = 0

chi2( 9) = 44.90

Prob > chi2 = 0.0000

. glm loneliness i.year##i.schoollevel##i.urbanrural i.gender i.year##i.immigrant i.degree, family(gamma) li

> nk (log) vce(robust) nolog

Generalized linear models Number of obs = 557,391

Optimization : ML Residual df = 557,341

Scale parameter = .1957478

Deviance = 113833.779 (1/df) Deviance = .2042444

Pearson = 109098.2813 (1/df) Pearson = .1957478

Variance function: V(u) = u^2 [Gamma]

Link function : g(u) = ln(u) [Log]

AIC = 3.667832

Log pseudolikelihood = -1022158.137 BIC = -7260357

--------------------------------------------------------------------------------------------------------

| Robust

loneliness | Coefficient std. err. z P>|z| [95% conf. interval]

---------------------------------------+----------------------------------------------------------------

year |

2019 | .0205264 .0031009 6.62 0.000 .0144488 .026604

2021 | .1246196 .0030148 41.34 0.000 .1187107 .1305286

2023 | .1124838 .003068 36.66 0.000 .1064707 .118497

|

schoollevel |

upper secondary | .0586048 .0035742 16.40 0.000 .0515995 .0656101

vocational | .020926 .0041266 5.07 0.000 .012838 .0290141

|

year#schoollevel |

2019#upper secondary | .0134191 .0046821 2.87 0.004 .0042425 .0225958

2019#vocational | .0034763 .0058461 0.59 0.552 -.0079818 .0149344

2021#upper secondary | .0012717 .004542 0.28 0.779 -.0076305 .0101738

2021#vocational | .0211231 .0057412 3.68 0.000 .0098706 .0323756

2023#upper secondary | -.0149715 .0046254 -3.24 0.001 -.0240371 -.0059059

2023#vocational | .0137889 .0060047 2.30 0.022 .0020199 .0255579

|

urbanrural |

semi-urban | -.0079135 .0047618 -1.66 0.097 -.0172465 .0014195

rural | -.0031807 .0051059 -0.62 0.533 -.0131881 .0068267

|

year#urbanrural |

2019#semi-urban | .0013447 .0064235 0.21 0.834 -.0112452 .0139346

2019#rural | .0051125 .0069678 0.73 0.463 -.0085442 .0187692

2021#semi-urban | .0050353 .0062684 0.80 0.422 -.0072506 .0173212

2021#rural | .0022318 .0067956 0.33 0.743 -.0110872 .0155509

2023#semi-urban | -.008868 .006425 -1.38 0.168 -.0214607 .0037248

2023#rural | -.0027928 .0069781 -0.40 0.689 -.0164696 .010884

|

schoollevel#urbanrural |

upper secondary#semi-urban | -.0074028 .0080594 -0.92 0.358 -.023199 .0083933

upper secondary#rural | .0130803 .008889 1.47 0.141 -.0043418 .0305023

vocational#semi-urban | -.0060017 .009442 -0.64 0.525 -.0245077 .0125044

vocational#rural | .0055265 .0147399 0.37 0.708 -.0233631 .0344161

|

year#schoollevel#urbanrural |

2019#upper secondary#semi-urban | -.0098491 .0107457 -0.92 0.359 -.0309103 .0112121

2019#upper secondary#rural | -.0311584 .0121051 -2.57 0.010 -.054884 -.0074328

2019#vocational#semi-urban | -.0139853 .0135565 -1.03 0.302 -.0405556 .012585

2019#vocational#rural | -.0252776 .0205126 -1.23 0.218 -.0654816 .0149263

2021#upper secondary#semi-urban | .0040226 .0104333 0.39 0.700 -.0164262 .0244714

2021#upper secondary#rural | -.0125604 .0117268 -1.07 0.284 -.0355445 .0104236

2021#vocational#semi-urban | -.0294264 .013226 -2.22 0.026 -.0553489 -.0035039

2021#vocational#rural | -.0391407 .0201332 -1.94 0.052 -.078601 .0003197

2023#upper secondary#semi-urban | .0169922 .0108405 1.57 0.117 -.0042548 .0382392

2023#upper secondary#rural | .0125856 .0121501 1.04 0.300 -.0112282 .0363994

2023#vocational#semi-urban | -.0180002 .0137549 -1.31 0.191 -.0449593 .0089589

2023#vocational#rural | -.0283145 .0212219 -1.33 0.182 -.0699087 .0132796

|

gender |

girl | .2685826 .0012058 222.75 0.000 .2662193 .2709459

|

immigrant |

One foreign-born parent | .0430362 .0052824 8.15 0.000 .032683 .0533895

Born in Finland, foreign-born parents | -.0444 .0114578 -3.88 0.000 -.0668569 -.0219432

Student and parents born abroad | .126225 .009874 12.78 0.000 .1068724 .1455776

|

year#immigrant |

2019#One foreign-born parent | .0022371 .0069474 0.32 0.747 -.0113796 .0158539

2019 #|

Born in Finland, foreign-born parents | .0245103 .0151442 1.62 0.106 -.0051717 .0541924

2019#Student and parents born abroad | -.0197001 .0130224 -1.51 0.130 -.0452234 .0058233

2021#One foreign-born parent | .0090756 .0067589 1.34 0.179 -.0041715 .0223228

2021 #|

Born in Finland, foreign-born parents | .0281446 .0143904 1.96 0.050 -.0000602 .0563493

2021#Student and parents born abroad | -.0248048 .0123732 -2.00 0.045 -.0490558 -.0005538

2023#One foreign-born parent | -.0020672 .0068472 -0.30 0.763 -.0154876 .0113531

2023 #|

Born in Finland, foreign-born parents | .0195163 .014447 1.35 0.177 -.0087994 .047832

2023#Student and parents born abroad | -.068511 .01218 -5.62 0.000 -.0923834 -.0446386

|

1.degree | -.0098437 .0012458 -7.90 0.000 -.0122855 -.007402

_cons | .6044006 .0025505 236.97 0.000 .5994016 .6093996

--------------------------------------------------------------------------------------------------------

. testparm year#schoollevel#urbanrural

( 1) [loneliness]2019.year#20.schoollevel#2.urbanrural = 0

( 2) [loneliness]2019.year#20.schoollevel#3.urbanrural = 0

( 3) [loneliness]2019.year#30.schoollevel#2.urbanrural = 0

( 4) [loneliness]2019.year#30.schoollevel#3.urbanrural = 0

( 5) [loneliness]2021.year#20.schoollevel#2.urbanrural = 0

( 6) [loneliness]2021.year#20.schoollevel#3.urbanrural = 0

( 7) [loneliness]2021.year#30.schoollevel#2.urbanrural = 0

( 8) [loneliness]2021.year#30.schoollevel#3.urbanrural = 0

( 9) [loneliness]2023.year#20.schoollevel#2.urbanrural = 0

(10) [loneliness]2023.year#20.schoollevel#3.urbanrural = 0

(11) [loneliness]2023.year#30.schoollevel#2.urbanrural = 0

(12) [loneliness]2023.year#30.schoollevel#3.urbanrural = 0

chi2( 12) = 29.96

Prob > chi2 = 0.0028

. testparm year#immigrant

( 1) [loneliness]2019.year#2.immigrant = 0

( 2) [loneliness]2019.year#3.immigrant = 0

( 3) [loneliness]2019.year#4.immigrant = 0

( 4) [loneliness]2021.year#2.immigrant = 0

( 5) [loneliness]2021.year#3.immigrant = 0

( 6) [loneliness]2021.year#4.immigrant = 0

( 7) [loneliness]2023.year#2.immigrant = 0

( 8) [loneliness]2023.year#3.immigrant = 0

( 9) [loneliness]2023.year#4.immigrant = 0

chi2( 9) = 47.02

Prob > chi2 = 0.0000

. margins year#schoollevel#urbanrural

Predictive margins Number of obs = 557,391

Model VCE: Robust

Expression: Predicted mean loneliness, predict()

--------------------------------------------------------------------------------------------------

| Delta-method

| Margin std. err. z P>|z| [95% conf. interval]

---------------------------------+----------------------------------------------------------------

year#schoollevel#urbanrural |

2017#lower secondary#urban | 2.131264 .0048368 440.63 0.000 2.121784 2.140744

2017#lower secondary#semi-urban | 2.114464 .0088472 239.00 0.000 2.097124 2.131805

2017#lower secondary#rural | 2.124496 .0097146 218.69 0.000 2.105455 2.143536

2017#upper secondary#urban | 2.259898 .0062442 361.92 0.000 2.24766 2.272137

2017#upper secondary#semi-urban | 2.225549 .0131673 169.02 0.000 2.199741 2.251356

2017#upper secondary#rural | 2.282382 .0154196 148.02 0.000 2.25216 2.312604

2017#vocational#urban | 2.176332 .0074916 290.50 0.000 2.161649 2.191016

2017#vocational#semi-urban | 2.146258 .015939 134.65 0.000 2.115018 2.177498

2017#vocational#rural | 2.181444 .0292505 74.58 0.000 2.124114 2.238774

2019#lower secondary#urban | 2.1753 .0043112 504.57 0.000 2.166851 2.18375

2019#lower secondary#semi-urban | 2.161058 .0082845 260.85 0.000 2.144821 2.177295

2019#lower secondary#rural | 2.179507 .0094147 231.50 0.000 2.161054 2.197959

2019#upper secondary#urban | 2.337754 .0053709 435.27 0.000 2.327227 2.348281

2019#upper secondary#semi-urban | 2.282725 .0118192 193.14 0.000 2.25956 2.30589

2019#upper secondary#rural | 2.300311 .0145274 158.34 0.000 2.271838 2.328784

2019#vocational#urban | 2.229036 .0081409 273.81 0.000 2.21308 2.244991

2019#vocational#semi-urban | 2.170621 .0172308 125.97 0.000 2.136849 2.204393

2019#vocational#rural | 2.189668 .0283824 77.15 0.000 2.134039 2.245296

2021#lower secondary#urban | 2.414922 .004458 541.70 0.000 2.406184 2.423659

2021#lower secondary#semi-urban | 2.407981 .0087498 275.21 0.000 2.390832 2.42513

2021#lower secondary#rural | 2.412631 .0098769 244.27 0.000 2.393273 2.43199

2021#upper secondary#urban | 2.563935 .0054461 470.78 0.000 2.553261 2.57461

2021#upper secondary#semi-urban | 2.547939 .0122003 208.84 0.000 2.524027 2.571851

2021#upper secondary#rural | 2.562836 .0149337 171.61 0.000 2.533566 2.592105

2021#vocational#urban | 2.518632 .0089443 281.59 0.000 2.501102 2.536163

2021#vocational#semi-urban | 2.423977 .0182668 132.70 0.000 2.388175 2.45978

2021#vocational#rural | 2.433068 .030347 80.17 0.000 2.373589 2.492547

2023#lower secondary#urban | 2.379566 .0045672 521.02 0.000 2.370615 2.388518

2023#lower secondary#semi-urban | 2.339967 .0090275 259.20 0.000 2.322273 2.35766

2023#lower secondary#rural | 2.365394 .0103045 229.55 0.000 2.345198 2.38559

2023#upper secondary#urban | 2.485693 .0055638 446.76 0.000 2.474788 2.496598

2023#upper secondary#semi-urban | 2.46788 .0133131 185.37 0.000 2.441787 2.493973

2023#upper secondary#rural | 2.535127 .0162351 156.15 0.000 2.503307 2.566948

2023#vocational#urban | 2.463623 .0096627 254.96 0.000 2.444685 2.482562

2023#vocational#semi-urban | 2.36517 .0192398 122.93 0.000 2.32746 2.402879

2023#vocational#rural | 2.393775 .0334414 71.58 0.000 2.328231 2.459319

--------------------------------------------------------------------------------------------------

. margins year#immigrant

Predictive margins Number of obs = 557,391

Model VCE: Robust

Expression: Predicted mean loneliness, predict()

-----------------------------------------------------------------------------------------------------------

| Delta-method

| Margin std. err. z P>|z| [95% conf. interval]

------------------------------------------+----------------------------------------------------------------

year#immigrant |

2017#Student and parents born in Finland | 2.158305 .0029597 729.23 0.000 2.152504 2.164106

2017#One foreign-born parent | 2.253218 .0114782 196.30 0.000 2.230721 2.275715

2017 #|

Born in Finland, foreign-born parents | 2.064572 .0234713 87.96 0.000 2.01857 2.110575

2017#Student and parents born abroad | 2.448678 .0239311 102.32 0.000 2.401774 2.495582

2019#Student and parents born in Finland | 2.21092 .0027324 809.13 0.000 2.205565 2.216276

2019#One foreign-born parent | 2.313316 .0100291 230.66 0.000 2.29366 2.332973

2019 #|

Born in Finland, foreign-born parents | 2.16738 .0212881 101.81 0.000 2.125656 2.209104

2019#Student and parents born abroad | 2.45944 .020636 119.18 0.000 2.418994 2.499886

2021#Student and parents born in Finland | 2.452923 .0028534 859.64 0.000 2.447331 2.458516

2021#One foreign-born parent | 2.584139 .0104648 246.94 0.000 2.563628 2.60465

2021 #|

Born in Finland, foreign-born parents | 2.413372 .0208197 115.92 0.000 2.372566 2.454178

2021#Student and parents born abroad | 2.714752 .0199698 135.94 0.000 2.675612 2.753892

2023#Student and parents born in Finland | 2.405682 .0029923 803.95 0.000 2.399817 2.411547

2023#One foreign-born parent | 2.506287 .010466 239.47 0.000 2.485774 2.526801

2023 #|

Born in Finland, foreign-born parents | 2.346559 .0204441 114.78 0.000 2.306489 2.386628

2023#Student and parents born abroad | 2.548609 .017878 142.56 0.000 2.513568 2.583649

-----------------------------------------------------------------------------------------------------------

## 3.3. Belonging at school

. glm belonging i.year##i.gender##i.schoollevel i.degree i.immigrant i.urbanrural, family(gaussian) link (i

> dentity) vce(robust) nolog

Generalized linear models Number of obs = 556,424

Optimization : ML Residual df = 556,394

Scale parameter = .909666

Deviance = 506132.6965 (1/df) Deviance = .909666

Pearson = 506132.6965 (1/df) Pearson = .909666

Variance function: V(u) = 1 [Gaussian]

Link function : g(u) = u [Identity]

AIC = 2.743253

Log pseudolikelihood = -763175.9552 BIC = -6854563

--------------------------------------------------------------------------------------------------------

| Robust

belonging | Coefficient std. err. z P>|z| [95% conf. interval]

---------------------------------------+----------------------------------------------------------------

year |

2019 | -.3197274 .007474 -42.78 0.000 -.3343761 -.3050787

2021 | -.3337288 .0073879 -45.17 0.000 -.3482089 -.3192487

2023 | -.3591779 .0076076 -47.21 0.000 -.3740884 -.3442673

|

gender |

girl | -.4072035 .0078581 -51.82 0.000 -.422605 -.3918019

|

year#gender |

2019#girl | .0947296 .0103925 9.12 0.000 .0743606 .1150985

2021#girl | .0206229 .0102941 2.00 0.045 .0004467 .0407991

2023#girl | -.0314892 .0105463 -2.99 0.003 -.0521596 -.0108187

|

schoollevel |

upper secondary | -.1278339 .009909 -12.90 0.000 -.1472552 -.1084127

vocational | .1242068 .0097667 12.72 0.000 .1050645 .1433492

|

year#schoollevel |

2019#upper secondary | .2131362 .0130468 16.34 0.000 .1875649 .2387074

2019#vocational | .0702029 .0132766 5.29 0.000 .0441812 .0962246

2021#upper secondary | .1250464 .0129718 9.64 0.000 .0996221 .1504707

2021#vocational | .0552567 .0135159 4.09 0.000 .028766 .0817474

2023#upper secondary | .2343913 .0132099 17.74 0.000 .2085002 .2602823

2023#vocational | .1168159 .0142549 8.19 0.000 .0888768 .1447551

|

gender#schoollevel |

girl#upper secondary | .1010247 .0131221 7.70 0.000 .0753057 .1267436

girl#vocational | .0652711 .0148596 4.39 0.000 .0361469 .0943953

|

year#gender#schoollevel |

2019#girl#upper secondary | -.0695093 .0172865 -4.02 0.000 -.1033903 -.0356283

2019#girl#vocational | -.0664729 .0203711 -3.26 0.001 -.1063996 -.0265463

2021#girl#upper secondary | -.0242083 .0172039 -1.41 0.159 -.0579272 .0095107

2021#girl#vocational | -.0286111 .0207682 -1.38 0.168 -.069316 .0120939

2023#girl#upper secondary | .04634 .0174886 2.65 0.008 .012063 .0806171

2023#girl#vocational | -.0246491 .0217871 -1.13 0.258 -.0673511 .0180528

|

1.degree | .0928942 .0026874 34.57 0.000 .0876271 .0981613

|

immigrant |

One foreign-born parent | -.0556687 .0050751 -10.97 0.000 -.0656157 -.0457216

Born in Finland, foreign-born parents | .0426206 .0095013 4.49 0.000 .0239985 .0612427

Student and parents born abroad | -.0965318 .0084501 -11.42 0.000 -.1130937 -.0799698

|

urbanrural |

semi-urban | .0306554 .0034684 8.84 0.000 .0238575 .0374532

rural | .0581469 .0041361 14.06 0.000 .0500403 .0662535

|

_cons | 3.852422 .0059506 647.40 0.000 3.840759 3.864085

--------------------------------------------------------------------------------------------------------

. testparm year#gender#schoollevel

( 1) [belonging]2019.year#2.gender#20.schoollevel = 0

( 2) [belonging]2019.year#2.gender#30.schoollevel = 0

( 3) [belonging]2021.year#2.gender#20.schoollevel = 0

( 4) [belonging]2021.year#2.gender#30.schoollevel = 0

( 5) [belonging]2023.year#2.gender#20.schoollevel = 0

( 6) [belonging]2023.year#2.gender#30.schoollevel = 0

chi2( 6) = 59.51

Prob > chi2 = 0.0000

. margins year#gender#schoollevel

Predictive margins Number of obs = 556,424

Model VCE: Robust

Expression: Predicted mean belonging, predict()

--------------------------------------------------------------------------------------------

| Delta-method

| Margin std. err. z P>|z| [95% conf. interval]

---------------------------+----------------------------------------------------------------

year#gender#schoollevel |

2017#boy#lower secondary | 3.909908 .005658 691.04 0.000 3.898818 3.920997

2017#boy#upper secondary | 3.782074 .0081336 464.99 0.000 3.766132 3.798016

2017#boy#vocational | 4.034115 .0079659 506.42 0.000 4.018502 4.049728

2017#girl#lower secondary | 3.502704 .0054663 640.78 0.000 3.491991 3.513418

2017#girl#upper secondary | 3.475895 .0066657 521.46 0.000 3.462831 3.48896

2017#girl#vocational | 3.692182 .0098349 375.42 0.000 3.672906 3.711458

2019#boy#lower secondary | 3.590181 .0048829 735.25 0.000 3.58061 3.599751

2019#boy#upper secondary | 3.675483 .0069684 527.45 0.000 3.661825 3.689141

2019#boy#vocational | 3.78459 .0076051 497.64 0.000 3.769684 3.799496

2019#girl#lower secondary | 3.277707 .0047304 692.90 0.000 3.268435 3.286978

2019#girl#upper secondary | 3.394524 .0056676 598.94 0.000 3.383416 3.405632

2019#girl#vocational | 3.470915 .009543 363.71 0.000 3.45221 3.489619

2021#boy#lower secondary | 3.576179 .0047473 753.31 0.000 3.566875 3.585484

2021#boy#upper secondary | 3.573392 .0069253 515.99 0.000 3.559818 3.586965

2021#boy#vocational | 3.755643 .0080937 464.02 0.000 3.739779 3.771506

2021#girl#lower secondary | 3.189599 .0046516 685.70 0.000 3.180482 3.198715

2021#girl#upper secondary | 3.263627 .0056584 576.78 0.000 3.252537 3.274718

2021#girl#vocational | 3.405722 .0100814 337.82 0.000 3.385963 3.425481

2023#boy#lower secondary | 3.55073 .0050786 699.16 0.000 3.540776 3.560684

2023#boy#upper secondary | 3.657287 .0071424 512.05 0.000 3.643288 3.671286

2023#boy#vocational | 3.791753 .0090906 417.11 0.000 3.773936 3.80957

2023#girl#lower secondary | 3.112037 .0048622 640.04 0.000 3.102508 3.121567

2023#girl#upper secondary | 3.365959 .0058086 579.48 0.000 3.354575 3.377344

2023#girl#vocational | 3.393682 .0110622 306.78 0.000 3.372001 3.415364

--------------------------------------------------------------------------------------------

. glm belonging i.year##i.gender##i.schoollevel##i.degree i.urbanrural i.immigrant, family(gaussian) link (i

> dentity) vce(robust) nolog

Generalized linear models Number of obs = 556,424

Optimization : ML Residual df = 556,371

Scale parameter = .9094141

Deviance = 505971.6553 (1/df) Deviance = .9094141

Pearson = 505971.6553 (1/df) Pearson = .9094141

Variance function: V(u) = 1 [Gaussian]

Link function : g(u) = u [Identity]

AIC = 2.743018

Log pseudolikelihood = -763087.4196 BIC = -6854419

--------------------------------------------------------------------------------------------------------

| Robust

belonging | Coefficient std. err. z P>|z| [95% conf. interval]

---------------------------------------+----------------------------------------------------------------

year |

2019 | -.3407934 .0107876 -31.59 0.000 -.3619368 -.31965

2021 | -.3434016 .0107566 -31.92 0.000 -.3644842 -.3223191

2023 | -.3699159 .011215 -32.98 0.000 -.3918969 -.347935

|

gender |

girl | -.3878039 .0110485 -35.10 0.000 -.4094586 -.3661492

|

year#gender |

2019#girl | .0754318 .0149676 5.04 0.000 .0460959 .1047677

2021#girl | -.0076198 .0149443 -0.51 0.610 -.0369101 .0216704

2023#girl | -.0524814 .0155252 -3.38 0.001 -.0829103 -.0220525

|

schoollevel |

upper secondary | -.1146437 .0163311 -7.02 0.000 -.1466521 -.0826353

vocational | .1295228 .0126524 10.24 0.000 .1047246 .154321

|

year#schoollevel |

2019#upper secondary | .2065601 .0222279 9.29 0.000 .1629943 .250126

2019#vocational | .1002756 .0173945 5.76 0.000 .0661829 .1343683

2021#upper secondary | .1124417 .0224861 5.00 0.000 .0683698 .1565136

2021#vocational | .0810331 .017793 4.55 0.000 .0461594 .1159067

2023#upper secondary | .2173259 .0233832 9.29 0.000 .1714957 .2631561

2023#vocational | .154714 .0190834 8.11 0.000 .1173111 .1921168

|

gender#schoollevel |

girl#upper secondary | .0754284 .0209375 3.60 0.000 .0343917 .1164651

girl#vocational | .0627582 .0188151 3.34 0.001 .0258813 .0996351

|

year#gender#schoollevel |

2019#girl#upper secondary | -.0363527 .028429 -1.28 0.201 -.0920725 .0193671

2019#girl#vocational | -.0580335 .026056 -2.23 0.026 -.1091022 -.0069647

2021#girl#upper secondary | -.0160381 .028804 -0.56 0.578 -.0724928 .0404166

2021#girl#vocational | -.0334561 .0267224 -1.25 0.211 -.085831 .0189187

2023#girl#upper secondary | .0611789 .0299657 2.04 0.041 .0024471 .1199107

2023#girl#vocational | -.0171216 .0284921 -0.60 0.548 -.0729652 .0387219

|

1.degree | .0844395 .0113181 7.46 0.000 .0622564 .1066225

|

year#degree |

2019 1 | .039849 .0149668 2.66 0.008 .0105146 .0691834

2021 1 | .0184501 .0148164 1.25 0.213 -.0105896 .0474898

2023 1 | .0200248 .0152926 1.31 0.190 -.0099482 .0499977

|

gender#degree |

girl#1 | -.0406371 .0157181 -2.59 0.010 -.071444 -.0098303

|

year#gender#degree |

2019#girl#1 | .0408049 .0208193 1.96 0.050 -2.08e-07 .08161

2021#girl#1 | .0565834 .0206551 2.74 0.006 .0161002 .0970666

2023#girl#1 | .0432116 .0212258 2.04 0.042 .0016097 .0848134

|

schoollevel#degree |

upper secondary#1 | -.017478 .0206981 -0.84 0.398 -.0580455 .0230894

vocational#1 | -.0186142 .0201953 -0.92 0.357 -.0581963 .0209679

|

year#schoollevel#degree |

2019#upper secondary#1 | .0006253 .0276833 0.02 0.982 -.053633 .0548836

2019#vocational#1 | -.0607671 .0274025 -2.22 0.027 -.114475 -.0070592

2021#upper secondary#1 | .0143081 .0277729 0.52 0.606 -.0401257 .0687419

2021#vocational#1 | -.0573624 .0278406 -2.06 0.039 -.111929 -.0027958

2023#upper secondary#1 | .0198439 .0286139 0.69 0.488 -.0362383 .0759261

2023#vocational#1 | -.0775682 .0291617 -2.66 0.008 -.134724 -.0204123

|

gender#schoollevel#degree |

girl#upper secondary#1 | .0482662 .0270923 1.78 0.075 -.0048338 .1013662

girl#vocational#1 | -.0184566 .0314073 -0.59 0.557 -.0800137 .0431005

|

year#gender#schoollevel#degree |

2019#girl#upper secondary#1 | -.0587173 .0361105 -1.63 0.104 -.1294925 .012058

2019#girl#vocational#1 | -.010299 .0428596 -0.24 0.810 -.0943022 .0737042

2021#girl#upper secondary#1 | -.0248821 .0362459 -0.69 0.492 -.0959227 .0461585

2021#girl#vocational#1 | .0415952 .043493 0.96 0.339 -.0436495 .1268398

2023#girl#upper secondary#1 | -.0327377 .0372856 -0.88 0.380 -.1058161 .0403407

2023#girl#vocational#1 | -.0008357 .045199 -0.02 0.985 -.0894241 .0877527

|

urbanrural |

semi-urban | .0310098 .0034686 8.94 0.000 .0242114 .0378082

rural | .0595672 .0041389 14.39 0.000 .051455 .0676793

|

immigrant |

One foreign-born parent | -.0554113 .0050748 -10.92 0.000 -.0653577 -.045465

Born in Finland, foreign-born parents | .0439236 .0095034 4.62 0.000 .0252972 .0625499

Student and parents born abroad | -.0952301 .0084522 -11.27 0.000 -.111796 -.0786641

|

_cons | 3.856183 .0081096 475.51 0.000 3.840289 3.872078

--------------------------------------------------------------------------------------------------------

. testparm year#schoollevel#gender#degree

( 1) [belonging]2019.year#2.gender#20.schoollevel#1.degree = 0

( 2) [belonging]2019.year#2.gender#30.schoollevel#1.degree = 0

( 3) [belonging]2021.year#2.gender#20.schoollevel#1.degree = 0

( 4) [belonging]2021.year#2.gender#30.schoollevel#1.degree = 0

( 5) [belonging]2023.year#2.gender#20.schoollevel#1.degree = 0

( 6) [belonging]2023.year#2.gender#30.schoollevel#1.degree = 0

chi2( 6) = 4.46

Prob > chi2 = 0.6147

.

. glm belonging i.year##i.gender##i.schoollevel##i.urbanrural i.degree i.immigrant, family(gaussian) link (i

> dentity) vce(robust) nolog

Generalized linear models Number of obs = 556,424

Optimization : ML Residual df = 556,348

Scale parameter = .9093461

Deviance = 505912.8914 (1/df) Deviance = .9093461

Pearson = 505912.8914 (1/df) Pearson = .9093461

Variance function: V(u) = 1 [Gaussian]

Link function : g(u) = u [Identity]

AIC = 2.742984

Log pseudolikelihood = -763055.106 BIC = -6854174

--------------------------------------------------------------------------------------------------------

| Robust

belonging | Coefficient std. err. z P>|z| [95% conf. interval]

---------------------------------------+----------------------------------------------------------------

year |

2019 | -.2990208 .0091984 -32.51 0.000 -.3170493 -.2809923

2021 | -.3214229 .0091008 -35.32 0.000 -.3392603 -.3035856

2023 | -.3587836 .0093753 -38.27 0.000 -.3771588 -.3404084

|

gender |

girl | -.4115021 .0097929 -42.02 0.000 -.4306959 -.3923084

|

year#gender |

2019#girl | .0961904 .0127737 7.53 0.000 .0711545 .1212264

2021#girl | .0262222 .0126676 2.07 0.038 .0013941 .0510502

2023#girl | -.0129933 .0129539 -1.00 0.316 -.0383825 .0123959

|

schoollevel |

upper secondary | -.1532179 .0119873 -12.78 0.000 -.1767125 -.1297232

vocational | .1214366 .0115903 10.48 0.000 .0987201 .1441531

|

year#schoollevel |

2019#upper secondary | .2137605 .0156114 13.69 0.000 .1831628 .2443582

2019#vocational | .0467609 .0156121 3.00 0.003 .0161618 .0773601

2021#upper secondary | .1359783 .0155043 8.77 0.000 .1055903 .1663662

2021#vocational | .0242094 .015947 1.52 0.129 -.0070462 .055465

2023#upper secondary | .2563302 .0157403 16.29 0.000 .2254798 .2871805

2023#vocational | .1032302 .0169435 6.09 0.000 .0700215 .136439

|

gender#schoollevel |

girl#upper secondary | .118025 .0158665 7.44 0.000 .0869272 .1491228

girl#vocational | .0846673 .0173478 4.88 0.000 .0506663 .1186684

|

year#gender#schoollevel |

2019#girl#upper secondary | -.0883909 .0206799 -4.27 0.000 -.1289228 -.0478589

2019#girl#vocational | -.0782221 .0236107 -3.31 0.001 -.1244983 -.031946

2021#girl#upper secondary | -.030154 .0205513 -1.47 0.142 -.0704338 .0101258

2021#girl#vocational | -.0190236 .0241423 -0.79 0.431 -.0663416 .0282943

2023#girl#upper secondary | .027875 .0208032 1.34 0.180 -.0128985 .0686485

2023#girl#vocational | -.0448855 .0254063 -1.77 0.077 -.0946809 .0049098

|

urbanrural |

semi-urban | .0424676 .0146741 2.89 0.004 .013707 .0712283

rural | .0679766 .0155677 4.37 0.000 .0374645 .0984888

|

year#urbanrural |

2019#semi-urban | -.0654191 .0196136 -3.34 0.001 -.1038611 -.026977

2019#rural | -.059371 .021262 -2.79 0.005 -.1010438 -.0176983

2021#semi-urban | -.0325136 .0194904 -1.67 0.095 -.0707141 .0056869

2021#rural | -.04316 .0208085 -2.07 0.038 -.083944 -.002376

2023#semi-urban | .0041801 .0198835 0.21 0.833 -.0347909 .0431511

2023#rural | -.0053588 .0216294 -0.25 0.804 -.0477516 .037034

|

gender#urbanrural |

girl#semi-urban | .0061535 .0203483 0.30 0.762 -.0337283 .0460354

girl#rural | .0204104 .021854 0.93 0.350 -.0224227 .0632435

|

year#gender#urbanrural |

2019#girl#semi-urban | .0066445 .027204 0.24 0.807 -.0466743 .0599632

2019#girl#rural | -.0183534 .0297896 -0.62 0.538 -.0767398 .0400331

2021#girl#semi-urban | -.006818 .0269725 -0.25 0.800 -.0596832 .0460472

2021#girl#rural | -.0292706 .0293691 -1.00 0.319 -.0868331 .0282918

2023#girl#semi-urban | -.0538041 .0275918 -1.95 0.051 -.1078831 .0002749

2023#girl#rural | -.0641429 .0303716 -2.11 0.035 -.1236701 -.0046156

|

schoollevel#urbanrural |

upper secondary#semi-urban | .0797393 .0262175 3.04 0.002 .028354 .1311247

upper secondary#rural | .1129571 .0294402 3.84 0.000 .0552553 .1706588

vocational#semi-urban | .0402141 .0246303 1.63 0.103 -.0080603 .0884885

vocational#rural | -.0760909 .0401056 -1.90 0.058 -.1546963 .0025146

|

year#schoollevel#urbanrural |

2019#upper secondary#semi-urban | -.0149014 .0349609 -0.43 0.670 -.0834234 .0536207

2019#upper secondary#rural | -.0018103 .040241 -0.04 0.964 -.0806811 .0770606

2019#vocational#semi-urban | .0340997 .0341475 1.00 0.318 -.0328281 .1010275

2019#vocational#rural | .2312837 .0545444 4.24 0.000 .1243787 .3381886

2021#upper secondary#semi-urban | -.0314742 .0348797 -0.90 0.367 -.0998371 .0368887

2021#upper secondary#rural | -.0613038 .0402403 -1.52 0.128 -.1401734 .0175658

2021#vocational#semi-urban | .0717411 .0343727 2.09 0.037 .0043719 .1391103

2021#vocational#rural | .2599454 .0550598 4.72 0.000 .1520302 .3678607

2023#upper secondary#semi-urban | -.0870124 .0359668 -2.42 0.016 -.1575061 -.0165188

2023#upper secondary#rural | -.0568603 .0411345 -1.38 0.167 -.1374824 .0237618

2023#vocational#semi-urban | .0210662 .0355341 0.59 0.553 -.0485794 .0907117

2023#vocational#rural | .1202132 .0581008 2.07 0.039 .0063378 .2340887

|

gender#schoollevel#urbanrural |

girl#upper secondary#semi-urban | -.0573749 .0348314 -1.65 0.100 -.1256433 .0108934

girl#upper secondary#rural | -.0580903 .0391561 -1.48 0.138 -.1348348 .0186541

girl#vocational#semi-urban | -.1036306 .0394694 -2.63 0.009 -.1809892 -.0262719

girl#vocational#rural | .0062204 .0608172 0.10 0.919 -.1129792 .12542

|

year#gender#schoollevel#urbanrural |

2019#girl#upper secondary#semi-urban | .066947 .0463982 1.44 0.149 -.0239918 .1578858

2019#girl#upper secondary#rural | .0674255 .053297 1.27 0.206 -.0370347 .1718857

2019#girl#vocational#semi-urban | .0565704 .0549371 1.03 0.303 -.0511043 .1642451

2019#girl#vocational#rural | -.0136279 .0823643 -0.17 0.869 -.1750589 .1478031

2021#girl#upper secondary#semi-urban | .0026469 .0464046 0.06 0.955 -.0883044 .0935982

2021#girl#upper secondary#rural | .0251825 .0533614 0.47 0.637 -.0794039 .129769

2021#girl#vocational#semi-urban | -.0039178 .055403 -0.07 0.944 -.1125056 .10467

2021#girl#vocational#rural | -.1849012 .0833363 -2.22 0.027 -.3482373 -.0215651

2023#girl#upper secondary#semi-urban | .0746581 .047803 1.56 0.118 -.0190341 .1683502

2023#girl#upper secondary#rural | .0101541 .0548263 0.19 0.853 -.0973034 .1176116

2023#girl#vocational#semi-urban | .1093113 .057091 1.91 0.056 -.002585 .2212076

2023#girl#vocational#rural | .0044019 .0894139 0.05 0.961 -.1708462 .1796499

|

1.degree | .0926523 .002688 34.47 0.000 .0873839 .0979206

|

immigrant |

One foreign-born parent | -.0558103 .0050742 -11.00 0.000 -.0657554 -.0458651

Born in Finland, foreign-born parents | .0416568 .0094989 4.39 0.000 .0230392 .0602744

Student and parents born abroad | -.0969277 .0084494 -11.47 0.000 -.1134883 -.0803671

|

_cons | 3.848736 .0072402 531.58 0.000 3.834545 3.862926

--------------------------------------------------------------------------------------------------------

. testparm year#schoollevel#gender#urbanrural

( 1) [belonging]2019.year#2.gender#20.schoollevel#2.urbanrural = 0

( 2) [belonging]2019.year#2.gender#20.schoollevel#3.urbanrural = 0

( 3) [belonging]2019.year#2.gender#30.schoollevel#2.urbanrural = 0

( 4) [belonging]2019.year#2.gender#30.schoollevel#3.urbanrural = 0

( 5) [belonging]2021.year#2.gender#20.schoollevel#2.urbanrural = 0

( 6) [belonging]2021.year#2.gender#20.schoollevel#3.urbanrural = 0

( 7) [belonging]2021.year#2.gender#30.schoollevel#2.urbanrural = 0

( 8) [belonging]2021.year#2.gender#30.schoollevel#3.urbanrural = 0

( 9) [belonging]2023.year#2.gender#20.schoollevel#2.urbanrural = 0

(10) [belonging]2023.year#2.gender#20.schoollevel#3.urbanrural = 0

(11) [belonging]2023.year#2.gender#30.schoollevel#2.urbanrural = 0

(12) [belonging]2023.year#2.gender#30.schoollevel#3.urbanrural = 0

chi2( 12) = 16.80

Prob > chi2 = 0.1575

.

. glm belonging i.year##i.gender##i.schoollevel##i.immigrant i.degree i.urbanrural, family(gaussian) link (i

> dentity) vce(robust) nolog

Generalized linear models Number of obs = 556,424

Optimization : ML Residual df = 556,325

Scale parameter = .9087881

Deviance = 505581.5187 (1/df) Deviance = .9087881

Pearson = 505581.5187 (1/df) Pearson = .9087881

Variance function: V(u) = 1 [Gaussian]

Link function : g(u) = u [Identity]

AIC = 2.742412

Log pseudolikelihood = -762872.8176 BIC = -6854201

----------------------------------------------------------------------------------------------------------

| Robust

belonging | Coefficient std. err. z P>|z| [95% conf. interval]

-----------------------------------------+----------------------------------------------------------------

year |

2019 | -.3262503 .0077949 -41.85 0.000 -.3415281 -.3109726

2021 | -.3391228 .0077117 -43.97 0.000 -.3542375 -.3240081

2023 | -.3673318 .007978 -46.04 0.000 -.3829685 -.3516952

|

gender |

girl | -.4215093 .0082117 -51.33 0.000 -.437604 -.4054146

|

year#gender |

2019#girl | .0992007 .0109026 9.10 0.000 .0778319 .1205694

2021#girl | .0171503 .0108152 1.59 0.113 -.0040472 .0383478

2023#girl | -.0391728 .0111246 -3.52 0.000 -.0609767 -.017369

|

schoollevel |

upper secondary | -.1354581 .0103384 -13.10 0.000 -.1557211 -.1151952

vocational | .1187819 .0100918 11.77 0.000 .0990024 .1385614

|

year#schoollevel |

2019#upper secondary | .2163922 .0136837 15.81 0.000 .1895727 .2432117

2019#vocational | .0701366 .0138115 5.08 0.000 .0430666 .0972065

2021#upper secondary | .1251211 .0136291 9.18 0.000 .0984087 .1518336

2021#vocational | .0546525 .014051 3.89 0.000 .0271131 .0821919

2023#upper secondary | .2373749 .0139278 17.04 0.000 .2100768 .264673

2023#vocational | .1117119 .0149798 7.46 0.000 .0823519 .1410718

|

gender#schoollevel |

girl#upper secondary | .1053707 .0137122 7.68 0.000 .0784952 .1322462

girl#vocational | .0695296 .0154361 4.50 0.000 .0392754 .0997839

|

year#gender#schoollevel |

2019#girl#upper secondary | -.0715338 .0181582 -3.94 0.000 -.1071232 -.0359444

2019#girl#vocational | -.0700886 .0212851 -3.29 0.001 -.1118065 -.0283706

2021#girl#upper secondary | -.0153614 .0181045 -0.85 0.396 -.0508456 .0201228

2021#girl#vocational | -.0328589 .0217011 -1.51 0.130 -.0753923 .0096744

2023#girl#upper secondary | .0453811 .0184767 2.46 0.014 .0091673 .0815948

2023#girl#vocational | -.020042 .0229455 -0.87 0.382 -.0650144 .0249305

|

immigrant |

One foreign-born parent | -.0691008 .022515 -3.07 0.002 -.1132295 -.0249722

Born in Finland, foreign-born parents | -.0753397 .0465078 -1.62 0.105 -.1664934 .0158139

Student and parents born abroad | -.3932946 .0408438 -9.63 0.000 -.4733469 -.3132422

|

year#immigrant |

2019#One foreign-born parent | .003293 .0298499 0.11 0.912 -.0552118 .0617978

2019 #|

Born in Finland, foreign-born parents | .048306 .0597441 0.81 0.419 -.0687902 .1654022

2019#Student and parents born abroad | .1353831 .0518735 2.61 0.009 .0337129 .2370534

2021#One foreign-born parent | -.0290665 .0295201 -0.98 0.325 -.0869248 .0287918

2021 #|

Born in Finland, foreign-born parents | .0958594 .0574567 1.67 0.095 -.0167536 .2084725

2021#Student and parents born abroad | .1560548 .0503865 3.10 0.002 .0572991 .2548105

2023#One foreign-born parent | -.0611194 .0300222 -2.04 0.042 -.1199618 -.002277

2023 #|

Born in Finland, foreign-born parents | .0635042 .0592112 1.07 0.283 -.0525476 .179556

2023#Student and parents born abroad | .2959701 .0493526 6.00 0.000 .1992407 .3926995

|

gender#immigrant |

girl#One foreign-born parent | .0293626 .0311872 0.94 0.346 -.0317631 .0904884

girl #|

Born in Finland, foreign-born parents | .0654562 .0641427 1.02 0.308 -.0602613 .1911736

girl#Student and parents born abroad | .2580747 .0574724 4.49 0.000 .1454308 .3707185

|

year#gender#immigrant |

2019#girl#One foreign-born parent | -.0289838 .0410563 -0.71 0.480 -.1094526 .051485

2019 #|

girl #|

Born in Finland, foreign-born parents | .0846701 .0810608 1.04 0.296 -.0742061 .2435463

2019 #|

girl #|

Student and parents born abroad | -.0889769 .0740128 -1.20 0.229 -.2340393 .0560855

2021#girl#One foreign-born parent | .035442 .0406341 0.87 0.383 -.0441994 .1150833

2021 #|

girl #|

Born in Finland, foreign-born parents | .0787028 .0787823 1.00 0.318 -.0757076 .2331133

2021 #|

girl #|

Student and parents born abroad | -.0171934 .0713684 -0.24 0.810 -.1570728 .122686

2023#girl#One foreign-born parent | .0869302 .04109 2.12 0.034 .0063953 .1674651

2023 #|

girl #|

Born in Finland, foreign-born parents | .1149802 .0805878 1.43 0.154 -.0429689 .2729293

2023 #|

girl #|

Student and parents born abroad | -.0245721 .0698876 -0.35 0.725 -.1615493 .1124051

|

schoollevel#immigrant |

upper secondary#One foreign-born parent | -.0175026 .040538 -0.43 0.666 -.0969555 .0619504

upper secondary #|

Born in Finland, foreign-born parents | -.0116163 .0820179 -0.14 0.887 -.1723684 .1491358

upper secondary #|

Student and parents born abroad | .1357942 .0804921 1.69 0.092 -.0219675 .2935558

vocational#One foreign-born parent | -.0077084 .0443331 -0.17 0.862 -.0945997 .0791828

vocational #|

Born in Finland, foreign-born parents | .0667369 .0922057 0.72 0.469 -.1139829 .2474567

vocational #|

Student and parents born abroad | .0391945 .071986 0.54 0.586 -.1018955 .1802844

|

year#schoollevel#immigrant |

2019 #|

upper secondary #|

One foreign-born parent | .0227749 .0527282 0.43 0.666 -.0805704 .1261203

2019 #|

upper secondary #|

Born in Finland, foreign-born parents | -.0590224 .1037525 -0.57 0.569 -.2623735 .1443287

2019 #|

upper secondary #|

Student and parents born abroad | -.0160706 .098282 -0.16 0.870 -.2086998 .1765587

2019#vocational#One foreign-born parent | -.0265539 .0587591 -0.45 0.651 -.1417196 .0886118

2019 #|

vocational #|

Born in Finland, foreign-born parents | .0577759 .1151505 0.50 0.616 -.167915 .2834668

2019 #|

vocational #|

Student and parents born abroad | .0849405 .0934727 0.91 0.363 -.0982626 .2681436

2021 #|

upper secondary #|

One foreign-born parent | .0826967 .0520348 1.59 0.112 -.0192896 .184683

2021 #|

upper secondary #|

Born in Finland, foreign-born parents | -.0643979 .1008257 -0.64 0.523 -.2620125 .1332168

2021 #|

upper secondary #|

Student and parents born abroad | -.0532961 .0966125 -0.55 0.581 -.2426532 .1360609

2021#vocational#One foreign-born parent | .0178152 .0599427 0.30 0.766 -.0996704 .1353008

2021 #|

vocational #|

Born in Finland, foreign-born parents | -.1586893 .1234933 -1.29 0.199 -.4007318 .0833532

2021 #|

vocational #|

Student and parents born abroad | .095211 .0944068 1.01 0.313 -.089823 .280245

2023 #|

upper secondary #|

One foreign-born parent | .0814073 .0526839 1.55 0.122 -.0218513 .1846659

2023 #|

upper secondary #|

Born in Finland, foreign-born parents | -.0493786 .10208 -0.48 0.629 -.2494517 .1506945

2023 #|

upper secondary #|

Student and parents born abroad | -.1046975 .093742 -1.12 0.264 -.2884284 .0790334

2023#vocational#One foreign-born parent | .0901132 .0612526 1.47 0.141 -.0299397 .2101662

2023 #|

vocational #|

Born in Finland, foreign-born parents | -.060193 .1190554 -0.51 0.613 -.2935373 .1731513

2023 #|

vocational #|

Student and parents born abroad | .0463228 .0899338 0.52 0.606 -.1299441 .2225898

|

gender#schoollevel#immigrant |

girl #|

upper secondary #|

One foreign-born parent | -.0020841 .0545086 -0.04 0.970 -.108919 .1047509

girl #|

upper secondary #|

Born in Finland, foreign-born parents | .1060977 .1081968 0.98 0.327 -.1059641 .3181596

girl #|

upper secondary #|

Student and parents born abroad | .0065839 .1048991 0.06 0.950 -.1990145 .2121823

girl#vocational#One foreign-born parent | .0133911 .0665469 0.20 0.841 -.1170384 .1438206

girl #|

vocational #|

Born in Finland, foreign-born parents | -.1077873 .1362034 -0.79 0.429 -.374741 .1591663

girl #|

vocational #|

Student and parents born abroad | .01938 .108377 0.18 0.858 -.193035 .2317951

|

year#gender#schoollevel#immigrant |

2019 #|

girl #|

upper secondary #|

One foreign-born parent | .0388488 .0703037 0.55 0.581 -.0989439 .1766414

2019 #|

girl #|

upper secondary #|

Born in Finland, foreign-born parents | -.129205 .1362081 -0.95 0.343 -.396168 .137758

2019 #|

girl #|

upper secondary #|

Student and parents born abroad | -.0899352 .1294172 -0.69 0.487 -.3435883 .1637178

2019 #|

girl #|

vocational #|

One foreign-born parent | .0395823 .088374 0.45 0.654 -.1336275 .2127922

2019 #|

girl #|

vocational #|

Born in Finland, foreign-born parents | -.2192428 .1743654 -1.26 0.209 -.5609927 .122507

2019 #|

girl #|

vocational #|

Student and parents born abroad | .0792704 .1421936 0.56 0.577 -.1994239 .3579647

2021 #|

girl #|

upper secondary #|

One foreign-born parent | -.0910682 .069376 -1.31 0.189 -.2270427 .0449063

2021 #|

girl #|

upper secondary #|

Born in Finland, foreign-born parents | -.1523328 .1331148 -1.14 0.252 -.4132331 .1085675

2021 #|

girl #|

upper secondary #|

Student and parents born abroad | -.0869602 .1271269 -0.68 0.494 -.3361244 .162204

2021 #|

girl #|

vocational #|

One foreign-born parent | .0138913 .0897989 0.15 0.877 -.1621114 .189894

2021 #|

girl #|

vocational #|

Born in Finland, foreign-born parents | .1745191 .184193 0.95 0.343 -.1864925 .5355308

2021 #|

girl #|

vocational #|

Student and parents born abroad | .0143916 .1432439 0.10 0.920 -.2663612 .2951445

2023 #|

girl #|

upper secondary #|

One foreign-born parent | -.0195436 .0698998 -0.28 0.780 -.1565446 .1174574

2023 #|

girl #|

upper secondary #|

Born in Finland, foreign-born parents | -.0834664 .1334602 -0.63 0.532 -.3450435 .1781108

2023 #|

girl #|

upper secondary #|

Student and parents born abroad | -.0657272 .1228947 -0.53 0.593 -.3065964 .175142

2023 #|

girl #|

vocational #|

One foreign-born parent | -.137728 .0913077 -1.51 0.131 -.3166878 .0412319

2023 #|

girl #|

vocational #|

Born in Finland, foreign-born parents | .1992245 .179301 1.11 0.267 -.152199 .550648

2023 #|

girl #|

vocational #|

Student and parents born abroad | .0049901 .1397433 0.04 0.972 -.2689018 .2788819

|

1.degree | .0928848 .0026866 34.57 0.000 .0876192 .0981504

|

urbanrural |

semi-urban | .0307553 .0034676 8.87 0.000 .023959 .0375516

rural | .0580069 .0041352 14.03 0.000 .049902 .0661118

|

_cons | 3.867601 .0061508 628.80 0.000 3.855546 3.879656

----------------------------------------------------------------------------------------------------------

. testparm year#schoollevel#gender#immigrant

( 1) [belonging]2019.year#2.gender#20.schoollevel#2.immigrant = 0

( 2) [belonging]2019.year#2.gender#20.schoollevel#3.immigrant = 0

( 3) [belonging]2019.year#2.gender#20.schoollevel#4.immigrant = 0

( 4) [belonging]2019.year#2.gender#30.schoollevel#2.immigrant = 0

( 5) [belonging]2019.year#2.gender#30.schoollevel#3.immigrant = 0

( 6) [belonging]2019.year#2.gender#30.schoollevel#4.immigrant = 0

( 7) [belonging]2021.year#2.gender#20.schoollevel#2.immigrant = 0

( 8) [belonging]2021.year#2.gender#20.schoollevel#3.immigrant = 0

( 9) [belonging]2021.year#2.gender#20.schoollevel#4.immigrant = 0

(10) [belonging]2021.year#2.gender#30.schoollevel#2.immigrant = 0

(11) [belonging]2021.year#2.gender#30.schoollevel#3.immigrant = 0

(12) [belonging]2021.year#2.gender#30.schoollevel#4.immigrant = 0

(13) [belonging]2023.year#2.gender#20.schoollevel#2.immigrant = 0

(14) [belonging]2023.year#2.gender#20.schoollevel#3.immigrant = 0

(15) [belonging]2023.year#2.gender#20.schoollevel#4.immigrant = 0

(16) [belonging]2023.year#2.gender#30.schoollevel#2.immigrant = 0

(17) [belonging]2023.year#2.gender#30.schoollevel#3.immigrant = 0

(18) [belonging]2023.year#2.gender#30.schoollevel#4.immigrant = 0

chi2( 18) = 22.35

Prob > chi2 = 0.2170

. glm belonging i.year##i.schoollevel##i.degree i.year##i.gender##i.degree i.urbanrural i.immigrant, family(

> gaussian) link (identity) vce(robust) nolog

Generalized linear models Number of obs = 556,424

Optimization : ML Residual df = 556,387

Scale parameter = .909861

Deviance = 506234.8155 (1/df) Deviance = .909861

Pearson = 506234.8155 (1/df) Pearson = .909861

Variance function: V(u) = 1 [Gaussian]

Link function : g(u) = u [Identity]

AIC = 2.74348

Log pseudolikelihood = -763232.0825 BIC = -6854368

--------------------------------------------------------------------------------------------------------

| Robust

belonging | Coefficient std. err. z P>|z| [95% conf. interval]

---------------------------------------+----------------------------------------------------------------

year |

2019 | -.3292966 .0093931 -35.06 0.000 -.3477068 -.3108864

2021 | -.3365039 .0094107 -35.76 0.000 -.3549484 -.3180594

2023 | -.3720738 .0098347 -37.83 0.000 -.3913494 -.3527982

|

schoollevel |

upper secondary | -.0702305 .0102083 -6.88 0.000 -.0902383 -.0502226

vocational | .1595667 .0093795 17.01 0.000 .1411831 .1779502

|

year#schoollevel |

2019#upper secondary | .186322 .0138367 13.47 0.000 .1592025 .2134415

2019#vocational | .0730768 .0129471 5.64 0.000 .0477009 .0984527

2021#upper secondary | .1043504 .0140347 7.44 0.000 .076843 .1318578

2021#vocational | .0654372 .013276 4.93 0.000 .0394166 .0914577

2023#upper secondary | .2546701 .014615 17.43 0.000 .2260253 .2833149

2023#vocational | .1475572 .0141688 10.41 0.000 .1197868 .1753276

|

1.degree | .0755489 .0098012 7.71 0.000 .0563388 .094759

|

year#degree |

2019 1 | .0491638 .0129968 3.78 0.000 .0236905 .0746372

2021 1 | .0182865 .0129137 1.42 0.157 -.007024 .0435969

2023 1 | .0163758 .0133298 1.23 0.219 -.0097502 .0425017

|

schoollevel#degree |

upper secondary#1 | .0057112 .0133227 0.43 0.668 -.0204009 .0318232

vocational#1 | -.02517 .0154233 -1.63 0.103 -.0553991 .0050592

|

year#schoollevel#degree |

2019#upper secondary#1 | -.0308636 .0177264 -1.74 0.082 -.0656066 .0038795

2019#vocational#1 | -.0648199 .0210052 -3.09 0.002 -.1059894 -.0236504

2021#upper secondary#1 | .0008306 .0178018 0.05 0.963 -.0340602 .0357214

2021#vocational#1 | -.0399344 .0213461 -1.87 0.061 -.0817721 .0019032

2023#upper secondary#1 | -.0004567 .0183142 -0.02 0.980 -.0363517 .0354384

2023#vocational#1 | -.0763856 .0222355 -3.44 0.001 -.1199664 -.0328048

|

gender |

girl | -.3571716 .008003 -44.63 0.000 -.3728571 -.341486

|

year#gender |

2019#girl | .0538233 .01095 4.92 0.000 .0323618 .0752848

2021#girl | -.0204444 .011064 -1.85 0.065 -.0421295 .0012407

2023#girl | -.0474183 .0115882 -4.09 0.000 -.0701308 -.0247058

|

gender#degree |

girl#1 | -.0227458 .0112884 -2.01 0.044 -.0448707 -.0006209

|

year#gender#degree |

2019#girl#1 | .0221983 .0150771 1.47 0.141 -.0073523 .051749

2021#girl#1 | .0559221 .0150839 3.71 0.000 .0263581 .085486

2023#girl#1 | .0484191 .0155523 3.11 0.002 .0179372 .078901

|

urbanrural |

semi-urban | .0307072 .0034682 8.85 0.000 .0239096 .0375049

rural | .0596466 .0041396 14.41 0.000 .051533 .0677601

|

immigrant |

One foreign-born parent | -.0556171 .0050755 -10.96 0.000 -.0655648 -.0456694

Born in Finland, foreign-born parents | .0435665 .0095064 4.58 0.000 .0249343 .0621987

Student and parents born abroad | -.0940781 .0084526 -11.13 0.000 -.1106449 -.0775114

|

_cons | 3.839977 .0070612 543.81 0.000 3.826137 3.853816

--------------------------------------------------------------------------------------------------------

. testparm year#schoollevel#degree

( 1) [belonging]2019.year#20.schoollevel#1.degree = 0

( 2) [belonging]2019.year#30.schoollevel#1.degree = 0

( 3) [belonging]2021.year#20.schoollevel#1.degree = 0

( 4) [belonging]2021.year#30.schoollevel#1.degree = 0

( 5) [belonging]2023.year#20.schoollevel#1.degree = 0

( 6) [belonging]2023.year#30.schoollevel#1.degree = 0

chi2( 6) = 18.87

Prob > chi2 = 0.0044

. testparm year#gender#degree

( 1) [belonging]2019.year#2.gender#1.degree = 0

( 2) [belonging]2021.year#2.gender#1.degree = 0

( 3) [belonging]2023.year#2.gender#1.degree = 0

chi2( 3) = 17.04

Prob > chi2 = 0.0007

. glm belonging i.year##i.schoollevel##i.urbanrural i.year##i.gender##i.urbanrural i.degree i.immigrant, fam

> ily(gaussian) link (identity) vce(robust) nolog

Generalized linear models Number of obs = 556,424

Optimization : ML Residual df = 556,372

Scale parameter = .9097968

Deviance = 506185.4548 (1/df) Deviance = .9097968

Pearson = 506185.4548 (1/df) Pearson = .9097968

Variance function: V(u) = 1 [Gaussian]

Link function : g(u) = u [Identity]

AIC = 2.743436

Log pseudolikelihood = -763204.9539 BIC = -6854219

--------------------------------------------------------------------------------------------------------

| Robust

belonging | Coefficient std. err. z P>|z| [95% conf. interval]

---------------------------------------+----------------------------------------------------------------

year |

2019 | -.2772691 .0078958 -35.12 0.000 -.2927446 -.2617937

2021 | -.3138265 .007837 -40.04 0.000 -.3291868 -.2984661

2023 | -.3592887 .0080562 -44.60 0.000 -.3750786 -.3434988

|

schoollevel |

upper secondary | -.0867406 .0078601 -11.04 0.000 -.1021461 -.0713351

vocational | .1625318 .0086349 18.82 0.000 .1456076 .1794559

|

year#schoollevel |

2019#upper secondary | .1643163 .0102354 16.05 0.000 .1442553 .1843774

2019#vocational | .0091836 .0117003 0.78 0.433 -.0137486 .0321158

2021#upper secondary | .1196978 .0101751 11.76 0.000 .099755 .1396406

2021#vocational | .0148171 .0119677 1.24 0.216 -.0086392 .0382733

2023#upper secondary | .2728592 .0102935 26.51 0.000 .2526842 .2930342

2023#vocational | .0837371 .0126214 6.63 0.000 .0589996 .1084746

|

urbanrural |

semi-urban | .0631793 .0127708 4.95 0.000 .038149 .0882097

rural | .0836477 .0139564 5.99 0.000 .0562937 .1110017

|

year#urbanrural |

2019#semi-urban | -.0833984 .0171181 -4.87 0.000 -.1169492 -.0498476

2019#rural | -.0751177 .0190582 -3.94 0.000 -.112471 -.0377644

2021#semi-urban | -.0355309 .0170378 -2.09 0.037 -.0689244 -.0021373

2021#rural | -.0427498 .0187397 -2.28 0.023 -.079479 -.0060207

2023#semi-urban | -.0133391 .0174202 -0.77 0.444 -.0474821 .0208039

2023#rural | -.0078083 .0194642 -0.40 0.688 -.0459575 .0303409

|

schoollevel#urbanrural |

upper secondary#semi-urban | .0481156 .0172582 2.79 0.005 .0142903 .081941

upper secondary#rural | .0803987 .0194098 4.14 0.000 .0423563 .1184412

vocational#semi-urban | -.0055941 .0191616 -0.29 0.770 -.0431501 .0319619

vocational#rural | -.0751222 .0301817 -2.49 0.013 -.1342773 -.0159671

|

year#schoollevel#urbanrural |

2019#upper secondary#semi-urban | .0224468 .0229806 0.98 0.329 -.0225943 .0674879

2019#upper secondary#rural | .0365421 .0263803 1.39 0.166 -.0151624 .0882466

2019#vocational#semi-urban | .0628787 .0266402 2.36 0.018 .0106648 .1150925

2019#vocational#rural | .2267409 .0409116 5.54 0.000 .1465556 .3069262

2021#upper secondary#semi-urban | -.0308629 .0230024 -1.34 0.180 -.0759468 .014221

2021#upper secondary#rural | -.0459385 .0264271 -1.74 0.082 -.0977347 .0058576

2021#vocational#semi-urban | .0706981 .026867 2.63 0.009 .0180397 .1233564

2021#vocational#rural | .1725711 .0414694 4.16 0.000 .0912926 .2538495

2023#upper secondary#semi-urban | -.046478 .0237009 -1.96 0.050 -.0929309 -.0000252

2023#upper secondary#rural | -.0521426 .0271997 -1.92 0.055 -.1054531 .0011679

2023#vocational#semi-urban | .0688412 .0276977 2.49 0.013 .0145547 .1231277

2023#vocational#rural | .121552 .0442126 2.75 0.006 .0348969 .2082071

|

gender |

girl | -.3601916 .0067957 -53.00 0.000 -.373511 -.3468722

|

year#gender |

2019#girl | .0550536 .0089637 6.14 0.000 .0374851 .0726221

2021#girl | .0120901 .0089505 1.35 0.177 -.0054526 .0296328

2023#girl | -.0117575 .0091496 -1.29 0.199 -.0296903 .0061754

|

gender#urbanrural |

girl#semi-urban | -.0333458 .0148555 -2.24 0.025 -.062462 -.0042296

girl#rural | -.009119 .0171287 -0.53 0.594 -.0426905 .0244526

|

year#gender#urbanrural |

2019#girl#semi-urban | .0407154 .0200215 2.03 0.042 .001474 .0799567

2019#girl#rural | .0110644 .0233779 0.47 0.636 -.0347554 .0568843

2021#girl#semi-urban | -.001345 .0199696 -0.07 0.946 -.0404847 .0377946

2021#girl#rural | -.030677 .0232403 -1.32 0.187 -.0762271 .0148731

2023#girl#semi-urban | -.0197707 .0205101 -0.96 0.335 -.0599697 .0204283

2023#girl#rural | -.0591718 .024052 -2.46 0.014 -.1063129 -.0120307

|

1.degree | .0916684 .0026874 34.11 0.000 .0864012 .0969357

|

immigrant |

One foreign-born parent | -.0560085 .0050752 -11.04 0.000 -.0659557 -.0460613

Born in Finland, foreign-born parents | .0413168 .0095012 4.35 0.000 .0226949 .0599387

Student and parents born abroad | -.0957195 .0084487 -11.33 0.000 -.1122787 -.0791603

|

_cons | 3.822218 .0062484 611.71 0.000 3.809971 3.834465

--------------------------------------------------------------------------------------------------------

. testparm year#schoollevel#urbanrural

( 1) [belonging]2019.year#20.schoollevel#2.urbanrural = 0

( 2) [belonging]2019.year#20.schoollevel#3.urbanrural = 0

( 3) [belonging]2019.year#30.schoollevel#2.urbanrural = 0

( 4) [belonging]2019.year#30.schoollevel#3.urbanrural = 0

( 5) [belonging]2021.year#20.schoollevel#2.urbanrural = 0

( 6) [belonging]2021.year#20.schoollevel#3.urbanrural = 0

( 7) [belonging]2021.year#30.schoollevel#2.urbanrural = 0

( 8) [belonging]2021.year#30.schoollevel#3.urbanrural = 0

( 9) [belonging]2023.year#20.schoollevel#2.urbanrural = 0

(10) [belonging]2023.year#20.schoollevel#3.urbanrural = 0

(11) [belonging]2023.year#30.schoollevel#2.urbanrural = 0

(12) [belonging]2023.year#30.schoollevel#3.urbanrural = 0

chi2( 12) = 66.77

Prob > chi2 = 0.0000

. testparm year#gender#urbanrural

( 1) [belonging]2019.year#2.gender#2.urbanrural = 0

( 2) [belonging]2019.year#2.gender#3.urbanrural = 0

( 3) [belonging]2021.year#2.gender#2.urbanrural = 0

( 4) [belonging]2021.year#2.gender#3.urbanrural = 0

( 5) [belonging]2023.year#2.gender#2.urbanrural = 0

( 6) [belonging]2023.year#2.gender#3.urbanrural = 0

chi2( 6) = 19.08

Prob > chi2 = 0.0040

. glm belonging i.year##i.schoollevel##i.immigrant i.year##i.gender##i.immigrant i.urbanrural i.degree, fami

> ly(gaussian) link (identity) vce(robust) nolog

Generalized linear models Number of obs = 556,424

Optimization : ML Residual df = 556,357

Scale parameter = .90927

Deviance = 505878.7427 (1/df) Deviance = .90927

Pearson = 505878.7427 (1/df) Pearson = .90927

Variance function: V(u) = 1 [Gaussian]

Link function : g(u) = u [Identity]

AIC = 2.742884

Log pseudolikelihood = -763036.3263 BIC = -6854327

----------------------------------------------------------------------------------------------------------

| Robust

belonging | Coefficient std. err. z P>|z| [95% conf. interval]

-----------------------------------------+----------------------------------------------------------------

year |

2019 | -.3090957 .0067747 -45.63 0.000 -.3223738 -.2958176

2021 | -.3336115 .0067256 -49.60 0.000 -.3467935 -.3204295

2023 | -.3722672 .0069494 -53.57 0.000 -.3858877 -.3586467

|

schoollevel |

upper secondary | -.0758451 .0068049 -11.15 0.000 -.0891824 -.0625078

vocational | .1521197 .0076591 19.86 0.000 .1371082 .1671313

|

year#schoollevel |

2019#upper secondary | .1763187 .0089918 19.61 0.000 .1586952 .1939422

2019#vocational | .0374032 .0105023 3.56 0.000 .0168191 .0579874

2021#upper secondary | .117222 .0089698 13.07 0.000 .0996416 .1348024

2021#vocational | .0399171 .0107081 3.73 0.000 .0189295 .0609046

2023#upper secondary | .263426 .0091537 28.78 0.000 .245485 .281367

2023#vocational | .1043524 .0113453 9.20 0.000 .082116 .1265887

|

immigrant |

One foreign-born parent | -.068118 .0198077 -3.44 0.001 -.1069404 -.0292956

Born in Finland, foreign-born parents | -.0800284 .0406548 -1.97 0.049 -.1597104 -.0003465

Student and parents born abroad | -.3871224 .0363595 -10.65 0.000 -.4583858 -.3158591

|

year#immigrant |

2019#One foreign-born parent | -.0080503 .026124 -0.31 0.758 -.0592525 .0431519

2019 #|

Born in Finland, foreign-born parents | .0751852 .0520266 1.45 0.148 -.0267851 .1771554

2019#Student and parents born abroad | .1332501 .0460822 2.89 0.004 .0429305 .2235696

2021#One foreign-born parent | -.0167853 .0258671 -0.65 0.516 -.0674839 .0339133

2021 #|

Born in Finland, foreign-born parents | .1055236 .0503925 2.09 0.036 .0067561 .2042911

2021#Student and parents born abroad | .1615131 .0448839 3.60 0.000 .0735423 .249484

2023#One foreign-born parent | -.0512019 .0262621 -1.95 0.051 -.1026748 .0002709

2023 #|

Born in Finland, foreign-born parents | .0578712 .0515156 1.12 0.261 -.0430975 .1588399

2023#Student and parents born abroad | .3016034 .0438463 6.88 0.000 .2156663 .3875405

|

schoollevel#immigrant |

upper secondary#One foreign-born parent | -.0187546 .0271168 -0.69 0.489 -.0719026 .0343934

upper secondary #|

Born in Finland, foreign-born parents | .0525938 .0535867 0.98 0.326 -.0524342 .1576218

upper secondary #|

Student and parents born abroad | .1335628 .0520064 2.57 0.010 .0316321 .2354935

vocational#One foreign-born parent | .0002679 .0331192 0.01 0.994 -.0646446 .0651803

vocational #|

Born in Finland, foreign-born parents | .0194636 .067939 0.29 0.775 -.1136945 .1526216

vocational #|

Student and parents born abroad | .0412411 .0540661 0.76 0.446 -.0647264 .1472086

|

year#schoollevel#immigrant |

2019 #|

upper secondary #|

One foreign-born parent | .0455733 .0348791 1.31 0.191 -.0227885 .1139351

2019 #|

upper secondary #|

Born in Finland, foreign-born parents | -.1347988 .067303 -2.00 0.045 -.2667102 -.0028874

2019 #|

upper secondary #|

Student and parents born abroad | -.0625574 .0642015 -0.97 0.330 -.18839 .0632752

2019#vocational#One foreign-born parent | -.0092266 .0439071 -0.21 0.834 -.0952829 .0768297

2019 #|

vocational #|

Born in Finland, foreign-born parents | -.0251513 .08664 -0.29 0.772 -.1949626 .14466

2019 #|

vocational #|

Student and parents born abroad | .1206972 .0706459 1.71 0.088 -.0177663 .2591607

2021 #|

upper secondary #|

One foreign-born parent | .0299696 .0344232 0.87 0.384 -.0374987 .0974378

2021 #|

upper secondary #|

Born in Finland, foreign-born parents | -.15554 .0659058 -2.36 0.018 -.2847129 -.0263671

2021 #|

upper secondary #|

Student and parents born abroad | -.0987251 .0630766 -1.57 0.118 -.2223531 .0249028

2021#vocational#One foreign-born parent | .021257 .0446894 0.48 0.634 -.0663327 .1088466

2021 #|

vocational #|

Born in Finland, foreign-born parents | -.0842324 .0916506 -0.92 0.358 -.2638642 .0953995

2021 #|

vocational #|

Student and parents born abroad | .1038612 .0711902 1.46 0.145 -.035669 .2433914

2023 #|

upper secondary #|

One foreign-born parent | .0720033 .0346301 2.08 0.038 .0041295 .1398771

2023 #|

upper secondary #|

Born in Finland, foreign-born parents | -.0983705 .0658442 -1.49 0.135 -.2274227 .0306817

2023 #|

upper secondary #|

Student and parents born abroad | -.1413562 .0609963 -2.32 0.020 -.2609067 -.0218056

2023#vocational#One foreign-born parent | .0234582 .0454739 0.52 0.606 -.065669 .1125854

2023 #|

vocational #|

Born in Finland, foreign-born parents | .0306638 .089102 0.34 0.731 -.1439729 .2053005

2023 #|

vocational #|

Student and parents born abroad | .0480747 .0688496 0.70 0.485 -.086868 .1830174

|

gender |

girl | -.3791898 .0058819 -64.47 0.000 -.3907182 -.3676613

|

year#gender |

2019#girl | .0667946 .0078717 8.49 0.000 .0513664 .0822228

2021#girl | .0068842 .0078649 0.88 0.381 -.0085308 .0222991

2023#girl | -.0293364 .0080977 -3.62 0.000 -.0452076 -.0134653

|

gender#immigrant |

girl#One foreign-born parent | .0265772 .0234672 1.13 0.257 -.0194177 .0725722

girl #|

Born in Finland, foreign-born parents | .0723889 .047632 1.52 0.129 -.0209681 .165746

girl#Student and parents born abroad | .2537411 .0430478 5.89 0.000 .1693691 .3381132

|

year#gender#immigrant |

2019#girl#One foreign-born parent | -.0081706 .0306686 -0.27 0.790 -.0682799 .0519387

2019 #|

girl #|

Born in Finland, foreign-born parents | .0363122 .0603248 0.60 0.547 -.0819222 .1545465

2019 #|

girl #|

Student and parents born abroad | -.0922067 .0548069 -1.68 0.092 -.1996263 .0152129

2021#girl#One foreign-born parent | .0127005 .0304309 0.42 0.676 -.0469429 .0723439

2021 #|

girl #|

Born in Finland, foreign-born parents | .0612813 .0592552 1.03 0.301 -.0548568 .1774194

2021 #|

girl #|

Student and parents born abroad | -.0333014 .0535579 -0.62 0.534 -.1382729 .0716701

2023#girl#One foreign-born parent | .0681255 .0307511 2.22 0.027 .0078545 .1283965

2023 #|

girl #|

Born in Finland, foreign-born parents | .1251403 .0597103 2.10 0.036 .0081102 .2421703

2023 #|

girl #|

Student and parents born abroad | -.0370176 .0520078 -0.71 0.477 -.1389511 .0649158

|

urbanrural |

semi-urban | .0304195 .0034672 8.77 0.000 .023624 .037215

rural | .0581155 .004136 14.05 0.000 .050009 .0662219

|

1.degree | .091824 .002686 34.19 0.000 .0865596 .0970884

_cons | 3.845893 .0054203 709.54 0.000 3.83527 3.856517

----------------------------------------------------------------------------------------------------------

. testparm year#schoollevel#immigrant

( 1) [belonging]2019.year#20.schoollevel#2.immigrant = 0

( 2) [belonging]2019.year#20.schoollevel#3.immigrant = 0

( 3) [belonging]2019.year#20.schoollevel#4.immigrant = 0

( 4) [belonging]2019.year#30.schoollevel#2.immigrant = 0

( 5) [belonging]2019.year#30.schoollevel#3.immigrant = 0

( 6) [belonging]2019.year#30.schoollevel#4.immigrant = 0

( 7) [belonging]2021.year#20.schoollevel#2.immigrant = 0

( 8) [belonging]2021.year#20.schoollevel#3.immigrant = 0

( 9) [belonging]2021.year#20.schoollevel#4.immigrant = 0

(10) [belonging]2021.year#30.schoollevel#2.immigrant = 0

(11) [belonging]2021.year#30.schoollevel#3.immigrant = 0

(12) [belonging]2021.year#30.schoollevel#4.immigrant = 0

(13) [belonging]2023.year#20.schoollevel#2.immigrant = 0

(14) [belonging]2023.year#20.schoollevel#3.immigrant = 0

(15) [belonging]2023.year#20.schoollevel#4.immigrant = 0

(16) [belonging]2023.year#30.schoollevel#2.immigrant = 0

(17) [belonging]2023.year#30.schoollevel#3.immigrant = 0

(18) [belonging]2023.year#30.schoollevel#4.immigrant = 0

chi2( 18) = 24.86

Prob > chi2 = 0.1288

. testparm year#gender#immigrant

( 1) [belonging]2019.year#2.gender#2.immigrant = 0

( 2) [belonging]2019.year#2.gender#3.immigrant = 0

( 3) [belonging]2019.year#2.gender#4.immigrant = 0

( 4) [belonging]2021.year#2.gender#2.immigrant = 0

( 5) [belonging]2021.year#2.gender#3.immigrant = 0

( 6) [belonging]2021.year#2.gender#4.immigrant = 0

( 7) [belonging]2023.year#2.gender#2.immigrant = 0

( 8) [belonging]2023.year#2.gender#3.immigrant = 0

( 9) [belonging]2023.year#2.gender#4.immigrant = 0

chi2( 9) = 16.63

Prob > chi2 = 0.0549

.

. glm belonging i.schoollevel i.year##i.gender##i.immigrant i.urbanrural i.degree, family(gaussian) link (id

> entity) vce(robust) nolog

Generalized linear models Number of obs = 556,424

Optimization : ML Residual df = 556,387

Scale parameter = .9109884

Deviance = 506862.121 (1/df) Deviance = .9109884

Pearson = 506862.121 (1/df) Pearson = .9109884

Variance function: V(u) = 1 [Gaussian]

Link function : g(u) = u [Identity]

AIC = 2.744718

Log pseudolikelihood = -763576.618 BIC = -6853741

--------------------------------------------------------------------------------------------------------

| Robust

belonging | Coefficient std. err. z P>|z| [95% conf. interval]

---------------------------------------+----------------------------------------------------------------

schoollevel |

upper secondary | .0726774 .0029263 24.84 0.000 .0669419 .0784129

vocational | .2034435 .0037046 54.92 0.000 .1961826 .2107044

|

year |

2019 | -.2566225 .005606 -45.78 0.000 -.26761 -.245635

2021 | -.2960036 .0056035 -52.82 0.000 -.3069863 -.2850209

2023 | -.2849702 .0058094 -49.05 0.000 -.2963564 -.273584

|

gender |

girl | -.3861065 .0058503 -66.00 0.000 -.3975728 -.3746401

|

year#gender |

2019#girl | .0768966 .0078053 9.85 0.000 .0615985 .0921948

2021#girl | .0119731 .0078015 1.53 0.125 -.0033175 .0272638

2023#girl | -.0168711 .0080433 -2.10 0.036 -.0326358 -.0011065

|

immigrant |

One foreign-born parent | -.0693218 .0168904 -4.10 0.000 -.1024264 -.0362171

Born in Finland, foreign-born parents | -.0612206 .0346935 -1.76 0.078 -.1292187 .0067775

Student and parents born abroad | -.3435781 .030736 -11.18 0.000 -.4038195 -.2833367

|

year#immigrant |

2019#One foreign-born parent | -.0003009 .0221917 -0.01 0.989 -.0437959 .0431941

2019 #|

Born in Finland, foreign-born parents | .0340692 .0441125 0.77 0.440 -.0523897 .1205282

2019#Student and parents born abroad | .1300544 .0388833 3.34 0.001 .0538445 .2062643

2021#One foreign-born parent | -.0094364 .0220779 -0.43 0.669 -.0527083 .0338355

2021 #|

Born in Finland, foreign-born parents | .0525335 .0433523 1.21 0.226 -.0324354 .1375024

2021#Student and parents born abroad | .1485476 .0381713 3.89 0.000 .0737332 .223362

2023#One foreign-born parent | -.0321891 .0224442 -1.43 0.152 -.0761789 .0118008

2023 #|

Born in Finland, foreign-born parents | .0322932 .0439201 0.74 0.462 -.0537886 .1183751

2023#Student and parents born abroad | .2649297 .0369995 7.16 0.000 .1924121 .3374473

|

gender#immigrant |

girl#One foreign-born parent | .0287053 .0234268 1.23 0.220 -.0172103 .0746209

girl #|

Born in Finland, foreign-born parents | .0757933 .0474227 1.60 0.110 -.0171535 .16874

girl#Student and parents born abroad | .259778 .0427165 6.08 0.000 .1760551 .3435008

|

year#gender#immigrant |

2019#girl#One foreign-born parent | -.0091012 .0305576 -0.30 0.766 -.068993 .0507906

2019 #|

girl #|

Born in Finland, foreign-born parents | .0275951 .0599126 0.46 0.645 -.0898314 .1450217

2019 #|

girl #|

Student and parents born abroad | -.0966481 .0543596 -1.78 0.075 -.203191 .0098947

2021#girl#One foreign-born parent | .0104926 .0303309 0.35 0.729 -.0489549 .0699401

2021 #|

girl #|

Born in Finland, foreign-born parents | .0570769 .0590427 0.97 0.334 -.0586447 .1727985

2021 #|

girl #|

Student and parents born abroad | -.0412556 .0531917 -0.78 0.438 -.1455095 .0629983

2023#girl#One foreign-born parent | .0672949 .0306831 2.19 0.028 .0071571 .1274328

2023 #|

girl #|

Born in Finland, foreign-born parents | .1166249 .0593773 1.96 0.050 .0002476 .2330022

2023 #|

girl #|

Student and parents born abroad | -.043475 .0515676 -0.84 0.399 -.1445458 .0575957

|

urbanrural |

semi-urban | .0301707 .0034692 8.70 0.000 .0233712 .0369702

rural | .0583995 .0041377 14.11 0.000 .0502897 .0665092

|

1.degree | .0923079 .0026878 34.34 0.000 .08704 .0975759

_cons | 3.79779 .0047603 797.80 0.000 3.78846 3.80712

--------------------------------------------------------------------------------------------------------

. testparm year#gender#immigrant

( 1) [belonging]2019.year#2.gender#2.immigrant = 0

( 2) [belonging]2019.year#2.gender#3.immigrant = 0

( 3) [belonging]2019.year#2.gender#4.immigrant = 0

( 4) [belonging]2021.year#2.gender#2.immigrant = 0

( 5) [belonging]2021.year#2.gender#3.immigrant = 0

( 6) [belonging]2021.year#2.gender#4.immigrant = 0

( 7) [belonging]2023.year#2.gender#2.immigrant = 0

( 8) [belonging]2023.year#2.gender#3.immigrant = 0

( 9) [belonging]2023.year#2.gender#4.immigrant = 0

chi2( 9) = 16.54

Prob > chi2 = 0.0565

. glm belonging i.year##i.immigrant i.schoollevel i.gender i.urbanrural i.degree, family(gaussian) link (ide

> ntity) vce(robust) nolog

Generalized linear models Number of obs = 556,424

Optimization : ML Residual df = 556,402

Scale parameter = .9117226

Deviance = 507284.2582 (1/df) Deviance = .9117226

Pearson = 507284.2582 (1/df) Pearson = .9117226

Variance function: V(u) = 1 [Gaussian]

Link function : g(u) = u [Identity]

AIC = 2.745497

Log pseudolikelihood = -763808.2288 BIC = -6853517

--------------------------------------------------------------------------------------------------------

| Robust

belonging | Coefficient std. err. z P>|z| [95% conf. interval]

---------------------------------------+----------------------------------------------------------------

year |

2019 | -.2161999 .0039073 -55.33 0.000 -.2238582 -.2085417

2021 | -.2899746 .0039105 -74.15 0.000 -.2976389 -.2823102

2023 | -.2942774 .0040358 -72.92 0.000 -.3021874 -.2863675

|

immigrant |

One foreign-born parent | -.0543757 .0117167 -4.64 0.000 -.0773399 -.0314114

Born in Finland, foreign-born parents | -.0206052 .0236769 -0.87 0.384 -.067011 .0258005

Student and parents born abroad | -.2269145 .0216927 -10.46 0.000 -.2694313 -.1843976

|

year#immigrant |

2019#One foreign-born parent | -.0033337 .0152641 -0.22 0.827 -.0332509 .0265835

2019 #|

Born in Finland, foreign-born parents | .0497653 .0298894 1.66 0.096 -.0088169 .1083474

2019#Student and parents born abroad | .0867491 .0275077 3.15 0.002 .0328351 .1406632

2021#One foreign-born parent | -.0028913 .0151431 -0.19 0.849 -.0325713 .0267887

2021 #|

Born in Finland, foreign-born parents | .0840776 .0294538 2.85 0.004 .0263492 .141806

2021#Student and parents born abroad | .1360559 .0269235 5.05 0.000 .0832868 .188825

2023#One foreign-born parent | .0046723 .0153027 0.31 0.760 -.0253205 .0346651

2023 #|

Born in Finland, foreign-born parents | .0972061 .0295789 3.29 0.001 .0392326 .1551796

2023#Student and parents born abroad | .2517564 .0261124 9.64 0.000 .2005771 .3029357

|

schoollevel |

upper secondary | .0730471 .0029271 24.96 0.000 .06731 .0787841

vocational | .2037801 .0037061 54.99 0.000 .1965163 .2110438

|

gender |

girl | -.3537603 .0025846 -136.87 0.000 -.358826 -.3486945

|

urbanrural |

semi-urban | .0300265 .0034706 8.65 0.000 .0232243 .0368288

rural | .0583306 .0041393 14.09 0.000 .0502178 .0664434

|

1.degree | .091983 .0026889 34.21 0.000 .0867129 .0972532

_cons | 3.780941 .0039168 965.32 0.000 3.773264 3.788617

--------------------------------------------------------------------------------------------------------

. testparm year#schoollevel#immigrant

no such variables;

the specified varlist does not identify any testable coefficients

r(111);

. testparm year#immigrant

( 1) [belonging]2019.year#2.immigrant = 0

( 2) [belonging]2019.year#3.immigrant = 0

( 3) [belonging]2019.year#4.immigrant = 0

( 4) [belonging]2021.year#2.immigrant = 0

( 5) [belonging]2021.year#3.immigrant = 0

( 6) [belonging]2021.year#4.immigrant = 0

( 7) [belonging]2023.year#2.immigrant = 0

( 8) [belonging]2023.year#3.immigrant = 0

( 9) [belonging]2023.year#4.immigrant = 0

chi2( 9) = 122.14

Prob > chi2 = 0.0000

. glm belonging i.year##i.schoollevel##i.degree i.year##i.gender##i.degree i.year##i.immigrant i.year##i.sch

> oollevel##i.urbanrural i.year##i.gender##i.urbanrural, family(gaussian) link (identity) vce(robust) nolog

Generalized linear models Number of obs = 556,424

Optimization : ML Residual df = 556,348

Scale parameter = .9092948

Deviance = 505884.3207 (1/df) Deviance = .9092948

Pearson = 505884.3207 (1/df) Pearson = .9092948

Variance function: V(u) = 1 [Gaussian]

Link function : g(u) = u [Identity]

AIC = 2.742928

Log pseudolikelihood = -763039.394 BIC = -6854202

--------------------------------------------------------------------------------------------------------

| Robust

belonging | Coefficient std. err. z P>|z| [95% conf. interval]

---------------------------------------+----------------------------------------------------------------

year |

2019 | -.3058763 .0110482 -27.69 0.000 -.3275304 -.2842221

2021 | -.3309987 .011038 -29.99 0.000 -.3526327 -.3093647

2023 | -.3869688 .0115086 -33.62 0.000 -.4095252 -.3644125

|

schoollevel |

upper secondary | -.0954078 .0116586 -8.18 0.000 -.1182583 -.0725573

vocational | .1641101 .0106663 15.39 0.000 .1432045 .1850157

|

year#schoollevel |

2019#upper secondary | .1779768 .0156389 11.38 0.000 .147325 .2086285

2019#vocational | .0429214 .0146837 2.92 0.003 .0141418 .071701

2021#upper secondary | .1184567 .0158126 7.49 0.000 .0874647 .1494488

2021#vocational | .0392285 .0150694 2.60 0.009 .0096931 .068764

2023#upper secondary | .2736668 .0163526 16.74 0.000 .2416164 .3057173

2023#vocational | .1291392 .0160799 8.03 0.000 .0976232 .1606553

|

1.degree | .0766576 .0099048 7.74 0.000 .0572447 .0960706

|

year#degree |

2019 1 | .040264 .013167 3.06 0.002 .0144571 .0660709

2021 1 | .0155667 .013065 1.19 0.233 -.0100402 .0411736

2023 1 | .0195527 .0135009 1.45 0.148 -.0069086 .046014

|

schoollevel#degree |

upper secondary#1 | .0146986 .0134764 1.09 0.275 -.0117146 .0411119

vocational#1 | -.0253576 .0154813 -1.64 0.101 -.0557005 .0049853

|

year#schoollevel#degree |

2019#upper secondary#1 | -.0267374 .0179329 -1.49 0.136 -.0618853 .0084105

2019#vocational#1 | -.0550731 .0211123 -2.61 0.009 -.0964525 -.0136937

2021#upper secondary#1 | -.0037346 .0180111 -0.21 0.836 -.0390356 .0315664

2021#vocational#1 | -.0322113 .0214492 -1.50 0.133 -.074251 .0098283

2023#upper secondary#1 | -.0049446 .0185256 -0.27 0.790 -.0412541 .031365

2023#vocational#1 | -.0729426 .0223418 -3.26 0.001 -.1167318 -.0291535

|

gender |

girl | -.3487456 .0092001 -37.91 0.000 -.3667775 -.3307138

|

year#gender |

2019#girl | .0427927 .0124817 3.43 0.001 .018329 .0672563

2021#girl | -.0169676 .0126011 -1.35 0.178 -.0416652 .0077301

2023#girl | -.0332354 .0131643 -2.52 0.012 -.059037 -.0074338

|

gender#degree |

girl#1 | -.02511 .011377 -2.21 0.027 -.0474085 -.0028115

|

year#gender#degree |

2019#girl#1 | .0256746 .0152102 1.69 0.091 -.0041369 .055486

2021#girl#1 | .0538618 .0152193 3.54 0.000 .0240325 .0836911

2023#girl#1 | .04213 .0157094 2.68 0.007 .0113401 .0729198

|

immigrant |

One foreign-born parent | -.0548387 .0117024 -4.69 0.000 -.077775 -.0319024

Born in Finland, foreign-born parents | -.0182331 .023709 -0.77 0.442 -.0647019 .0282358

Student and parents born abroad | -.2403149 .021746 -11.05 0.000 -.2829363 -.1976935

|

year#immigrant |

2019#One foreign-born parent | -.0056425 .0152593 -0.37 0.712 -.0355503 .0242652

2019 #|

Born in Finland, foreign-born parents | .0443746 .0299462 1.48 0.138 -.014319 .1030681

2019#Student and parents born abroad | .104703 .0275433 3.80 0.000 .0507192 .1586868

2021#One foreign-born parent | -.0035479 .0151425 -0.23 0.815 -.0332266 .0261308

2021 #|

Born in Finland, foreign-born parents | .0795914 .0295239 2.70 0.007 .0217256 .1374573

2021#Student and parents born abroad | .1475926 .026989 5.47 0.000 .0946951 .2004901

2023#One foreign-born parent | .0066481 .0152897 0.43 0.664 -.0233192 .0366155

2023 #|

Born in Finland, foreign-born parents | .096572 .0296537 3.26 0.001 .0384519 .1546921

2023#Student and parents born abroad | .2673523 .0261635 10.22 0.000 .2160728 .3186318

|

urbanrural |

semi-urban | .0572676 .0128562 4.45 0.000 .0320698 .0824654

rural | .0768079 .014061 5.46 0.000 .0492488 .1043669

|

year#urbanrural |

2019#semi-urban | -.0751991 .0172436 -4.36 0.000 -.108996 -.0414022

2019#rural | -.0643593 .0192539 -3.34 0.001 -.1020962 -.0266223

2021#semi-urban | -.0290776 .0171699 -1.69 0.090 -.0627299 .0045747

2021#rural | -.0352614 .0189057 -1.87 0.062 -.072316 .0017931

2023#semi-urban | -.0029353 .0175565 -0.17 0.867 -.0373454 .0314749

2023#rural | .0044276 .0196531 0.23 0.822 -.0340918 .0429469

|

schoollevel#urbanrural |

upper secondary#semi-urban | .0506176 .0173481 2.92 0.004 .0166159 .0846192

upper secondary#rural | .0837938 .0195762 4.28 0.000 .0454252 .1221623

vocational#semi-urban | -.0038965 .0191877 -0.20 0.839 -.0415036 .0337107

vocational#rural | -.0735358 .0302281 -2.43 0.015 -.1327818 -.0142898

|

year#schoollevel#urbanrural |

2019#upper secondary#semi-urban | .0184588 .0231022 0.80 0.424 -.0268206 .0637382

2019#upper secondary#rural | .0306985 .0266041 1.15 0.249 -.0214446 .0828416

2019#vocational#semi-urban | .0527903 .0267081 1.98 0.048 .0004433 .1051372

2019#vocational#rural | .2140987 .0409913 5.22 0.000 .1337571 .2944402

2021#upper secondary#semi-urban | -.0322918 .0231319 -1.40 0.163 -.0776294 .0130459

2021#upper secondary#rural | -.0472792 .0266412 -1.77 0.076 -.099495 .0049366

2021#vocational#semi-urban | .0617788 .0269364 2.29 0.022 .0089845 .1145732

2021#vocational#rural | .1632223 .0415407 3.93 0.000 .081804 .2446406

2023#upper secondary#semi-urban | -.0492953 .0238211 -2.07 0.039 -.0959839 -.0026067

2023#upper secondary#rural | -.0580544 .0274401 -2.12 0.034 -.111836 -.0042727

2023#vocational#semi-urban | .0552409 .0277608 1.99 0.047 .0008306 .1096511

2023#vocational#rural | .1063864 .0443197 2.40 0.016 .0195215 .1932513

|

gender#urbanrural |

girl#semi-urban | -.0365979 .014911 -2.45 0.014 -.0658229 -.007373

girl#rural | -.0131048 .0172109 -0.76 0.446 -.0468377 .020628

|

year#gender#urbanrural |

2019#girl#semi-urban | .0441299 .0201069 2.19 0.028 .0047212 .0835387

2019#girl#rural | .0154245 .0235124 0.66 0.512 -.030659 .061508

2021#girl#semi-urban | .0058365 .0200625 0.29 0.771 -.0334853 .0451582

2021#girl#rural | -.0210894 .0233695 -0.90 0.367 -.0668928 .0247141

2023#girl#semi-urban | -.0136755 .0206135 -0.66 0.507 -.0540773 .0267263

2023#girl#rural | -.0518861 .0242116 -2.14 0.032 -.09934 -.0044323

|

_cons | 3.838557 .0082426 465.70 0.000 3.822402 3.854712

--------------------------------------------------------------------------------------------------------

. testparm year#schoollevel#degree

( 1) [belonging]2019.year#20.schoollevel#1.degree = 0

( 2) [belonging]2019.year#30.schoollevel#1.degree = 0

( 3) [belonging]2021.year#20.schoollevel#1.degree = 0

( 4) [belonging]2021.year#30.schoollevel#1.degree = 0

( 5) [belonging]2023.year#20.schoollevel#1.degree = 0

( 6) [belonging]2023.year#30.schoollevel#1.degree = 0

chi2( 6) = 14.65

Prob > chi2 = 0.0232

. testparm year#gender#degree

( 1) [belonging]2019.year#2.gender#1.degree = 0

( 2) [belonging]2021.year#2.gender#1.degree = 0

( 3) [belonging]2023.year#2.gender#1.degree = 0

chi2( 3) = 13.85

Prob > chi2 = 0.0031

. testparm year#schoollevel#urbanrural

( 1) [belonging]2019.year#20.schoollevel#2.urbanrural = 0

( 2) [belonging]2019.year#20.schoollevel#3.urbanrural = 0

( 3) [belonging]2019.year#30.schoollevel#2.urbanrural = 0

( 4) [belonging]2019.year#30.schoollevel#3.urbanrural = 0

( 5) [belonging]2021.year#20.schoollevel#2.urbanrural = 0

( 6) [belonging]2021.year#20.schoollevel#3.urbanrural = 0

( 7) [belonging]2021.year#30.schoollevel#2.urbanrural = 0

( 8) [belonging]2021.year#30.schoollevel#3.urbanrural = 0

( 9) [belonging]2023.year#20.schoollevel#2.urbanrural = 0

(10) [belonging]2023.year#20.schoollevel#3.urbanrural = 0

(11) [belonging]2023.year#30.schoollevel#2.urbanrural = 0

(12) [belonging]2023.year#30.schoollevel#3.urbanrural = 0

chi2( 12) = 59.94

Prob > chi2 = 0.0000

. testparm year#gender#urbanrural

( 1) [belonging]2019.year#2.gender#2.urbanrural = 0

( 2) [belonging]2019.year#2.gender#3.urbanrural = 0

( 3) [belonging]2021.year#2.gender#2.urbanrural = 0

( 4) [belonging]2021.year#2.gender#3.urbanrural = 0

( 5) [belonging]2023.year#2.gender#2.urbanrural = 0

( 6) [belonging]2023.year#2.gender#3.urbanrural = 0

chi2( 6) = 16.72

Prob > chi2 = 0.0104

. testparm year#immigrant

( 1) [belonging]2019.year#2.immigrant = 0

( 2) [belonging]2019.year#3.immigrant = 0

( 3) [belonging]2019.year#4.immigrant = 0

( 4) [belonging]2021.year#2.immigrant = 0

( 5) [belonging]2021.year#3.immigrant = 0

( 6) [belonging]2021.year#4.immigrant = 0

( 7) [belonging]2023.year#2.immigrant = 0

( 8) [belonging]2023.year#3.immigrant = 0

( 9) [belonging]2023.year#4.immigrant = 0

chi2( 9) = 130.71

Prob > chi2 = 0.0000

. margins year#schoollevel#degree

Predictive margins Number of obs = 556,424

Model VCE: Robust

Expression: Predicted mean belonging, predict()

-----------------------------------------------------------------------------------------

| Delta-method

| Margin std. err. z P>|z| [95% conf. interval]

------------------------+----------------------------------------------------------------

year#schoollevel#degree |

2017#lower secondary#0 | 3.656263 .0056456 647.63 0.000 3.645198 3.667328

2017#lower secondary#1 | 3.719636 .0056008 664.12 0.000 3.708658 3.730613

2017#upper secondary#0 | 3.579368 .0086475 413.92 0.000 3.562419 3.596316

2017#upper secondary#1 | 3.657439 .0065143 561.44 0.000 3.644671 3.670207

2017#vocational#0 | 3.811034 .0077745 490.19 0.000 3.795796 3.826272

2017#vocational#1 | 3.849049 .0111178 346.20 0.000 3.827258 3.870839

2019#lower secondary#0 | 3.361534 .0051143 657.28 0.000 3.35151 3.371558

2019#lower secondary#1 | 3.478754 .0046089 754.78 0.000 3.469721 3.487788

2019#upper secondary#0 | 3.469383 .0078684 440.93 0.000 3.453961 3.484804

2019#upper secondary#1 | 3.574564 .0053776 664.72 0.000 3.564024 3.585104

2019#vocational#0 | 3.593481 .0075264 477.45 0.000 3.57873 3.608233

2019#vocational#1 | 3.630271 .0103344 351.28 0.000 3.610016 3.650526

2021#lower secondary#0 | 3.312805 .0050952 650.18 0.000 3.302818 3.322791

2021#lower secondary#1 | 3.420241 .0044378 770.70 0.000 3.411543 3.428939

2021#upper secondary#0 | 3.343285 .0082203 406.71 0.000 3.327173 3.359396

2021#upper secondary#1 | 3.461685 .0052666 657.29 0.000 3.451363 3.472007

2021#vocational#0 | 3.536589 .0080891 437.21 0.000 3.520735 3.552443

2021#vocational#1 | 3.586456 .0106663 336.24 0.000 3.565551 3.607362

2023#lower secondary#0 | 3.258917 .0055244 589.92 0.000 3.248089 3.269744

2023#lower secondary#1 | 3.364132 .0046056 730.44 0.000 3.355105 3.373159

2023#upper secondary#0 | 3.440438 .0089226 385.59 0.000 3.42295 3.457926

2023#upper secondary#1 | 3.555407 .0053382 666.03 0.000 3.544944 3.56587

2023#vocational#0 | 3.564792 .0093127 382.79 0.000 3.546539 3.583045

2023#vocational#1 | 3.571707 .0112727 316.85 0.000 3.549613 3.593801

-----------------------------------------------------------------------------------------

. margins year#gender#degree

Predictive margins Number of obs = 556,424

Model VCE: Robust

Expression: Predicted mean belonging, predict()

------------------------------------------------------------------------------------

| Delta-method

| Margin std. err. z P>|z| [95% conf. interval]

-------------------+----------------------------------------------------------------

year#gender#degree |

2017#boy#0 | 3.844899 .0060465 635.89 0.000 3.833048 3.85675

2017#boy#1 | 3.922161 .005689 689.43 0.000 3.911011 3.933311

2017#girl#0 | 3.48837 .0055776 625.42 0.000 3.477438 3.499302

2017#girl#1 | 3.540522 .0056186 630.15 0.000 3.52951 3.551534

2019#boy#0 | 3.587171 .0055606 645.11 0.000 3.576273 3.59807

2019#boy#1 | 3.688487 .0048043 767.74 0.000 3.67907 3.697903

2019#girl#0 | 3.282776 .0051467 637.84 0.000 3.272688 3.292863

2019#girl#1 | 3.384656 .0046924 721.31 0.000 3.375459 3.393853

2021#boy#0 | 3.552549 .0056633 627.29 0.000 3.541449 3.563649

2021#boy#1 | 3.639452 .0046779 778.01 0.000 3.630284 3.648621

2021#girl#0 | 3.177559 .0052836 601.40 0.000 3.167204 3.187915

2021#girl#1 | 3.293214 .0045936 716.91 0.000 3.284211 3.302217

2023#boy#0 | 3.568976 .0062444 571.55 0.000 3.556737 3.581215

2023#boy#1 | 3.653422 .0048813 748.46 0.000 3.643854 3.662989

2023#girl#0 | 3.17076 .0057382 552.57 0.000 3.159513 3.182006

2023#girl#1 | 3.272225 .0047024 695.87 0.000 3.263009 3.281442

------------------------------------------------------------------------------------

. margins year#schoollevel#urbanrural

Predictive margins Number of obs = 556,424

Model VCE: Robust

Expression: Predicted mean belonging, predict()

--------------------------------------------------------------------------------------------------

| Delta-method

| Margin std. err. z P>|z| [95% conf. interval]

---------------------------------+----------------------------------------------------------------

year#schoollevel#urbanrural |

2017#lower secondary#urban | 3.676851 .0048824 753.08 0.000 3.667281 3.68642

2017#lower secondary#semi-urban | 3.714755 .0090211 411.79 0.000 3.697074 3.732436

2017#lower secondary#rural | 3.746725 .0099327 377.21 0.000 3.727257 3.766193

2017#upper secondary#urban | 3.589649 .0062905 570.65 0.000 3.57732 3.601978

2017#upper secondary#semi-urban | 3.678171 .0125386 293.35 0.000 3.653596 3.702746

2017#upper secondary#rural | 3.743317 .0148239 252.52 0.000 3.714263 3.772371

2017#vocational#urban | 3.826804 .0076851 497.95 0.000 3.811741 3.841866

2017#vocational#semi-urban | 3.860812 .0150816 255.99 0.000 3.831252 3.890371

2017#vocational#rural | 3.823142 .0274992 139.03 0.000 3.769245 3.87704

2019#lower secondary#urban | 3.427762 .0041047 835.09 0.000 3.419717 3.435807

2019#lower secondary#semi-urban | 3.413815 .0080912 421.92 0.000 3.397957 3.429674

2019#lower secondary#rural | 3.441437 .0093515 368.01 0.000 3.423109 3.459766

2019#upper secondary#urban | 3.503609 .0053573 653.99 0.000 3.493109 3.514109

2019#upper secondary#semi-urban | 3.558739 .0110854 321.03 0.000 3.537012 3.580466

2019#upper secondary#rural | 3.631778 .0137895 263.37 0.000 3.604751 3.658804

2019#vocational#urban | 3.589889 .0071843 499.68 0.000 3.575808 3.60397

2019#vocational#semi-urban | 3.624836 .0151206 239.73 0.000 3.5952 3.654472

2019#vocational#rural | 3.744128 .0250749 149.32 0.000 3.694982 3.793274

2021#lower secondary#urban | 3.367903 .0040363 834.40 0.000 3.359992 3.375814

2021#lower secondary#semi-urban | 3.379818 .0079183 426.83 0.000 3.364298 3.395337

2021#lower secondary#rural | 3.391358 .0090319 375.49 0.000 3.373656 3.40906

2021#upper secondary#urban | 3.397073 .0054046 628.55 0.000 3.38648 3.407666

2021#upper secondary#semi-urban | 3.427314 .0113137 302.93 0.000 3.405139 3.449488

2021#upper secondary#rural | 3.457043 .0140725 245.66 0.000 3.429461 3.484624

2021#vocational#urban | 3.539101 .0076121 464.93 0.000 3.524181 3.55402

2021#vocational#semi-urban | 3.608898 .0153643 234.89 0.000 3.578785 3.639012

2021#vocational#rural | 3.652242 .0259342 140.83 0.000 3.601412 3.703073

2023#lower secondary#urban | 3.307278 .0042729 774.02 0.000 3.298904 3.315653

2023#lower secondary#semi-urban | 3.335012 .0083288 400.42 0.000 3.318688 3.351336

2023#lower secondary#rural | 3.354129 .0097314 344.67 0.000 3.335055 3.373202

2023#upper secondary#urban | 3.490983 .0056109 622.18 0.000 3.479986 3.50198

2023#upper secondary#semi-urban | 3.520039 .0123241 285.62 0.000 3.495884 3.544194

2023#upper secondary#rural | 3.563573 .0149967 237.62 0.000 3.53418 3.592966

2023#vocational#urban | 3.545647 .0083832 422.95 0.000 3.529216 3.562078

2023#vocational#semi-urban | 3.624725 .0160249 226.19 0.000 3.593317 3.656133

2023#vocational#rural | 3.625348 .0296608 122.23 0.000 3.567214 3.683482

--------------------------------------------------------------------------------------------------

. margins year#gender#urbanrural

Predictive margins Number of obs = 556,424

Model VCE: Robust

Expression: Predicted mean belonging, predict()

----------------------------------------------------------------------------------------

| Delta-method

| Margin std. err. z P>|z| [95% conf. interval]

-----------------------+----------------------------------------------------------------

year#gender#urbanrural |

2017#boy#urban | 3.866426 .0049519 780.80 0.000 3.856721 3.876132

2017#boy#semi-urban | 3.938232 .0094851 415.20 0.000 3.919641 3.956822

2017#boy#rural | 3.957283 .0115584 342.37 0.000 3.934629 3.979937

2017#girl#urban | 3.503662 .0047015 745.23 0.000 3.494447 3.512876

2017#girl#semi-urban | 3.538869 .0092859 381.10 0.000 3.520669 3.557069

2017#girl#rural | 3.581414 .0111808 320.32 0.000 3.5595 3.603328

2019#boy#urban | 3.638215 .0042337 859.35 0.000 3.629917 3.646513

2019#boy#semi-urban | 3.648218 .0086423 422.13 0.000 3.63128 3.665157

2019#boy#rural | 3.705853 .0109269 339.15 0.000 3.684436 3.727269

2019#girl#urban | 3.332577 .0040379 825.31 0.000 3.324663 3.340492

2019#girl#semi-urban | 3.350112 .008387 399.44 0.000 3.333674 3.366551

2019#girl#rural | 3.402534 .010317 329.80 0.000 3.382314 3.422755

2021#boy#urban | 3.589295 .0042201 850.53 0.000 3.581024 3.597566

2021#boy#semi-urban | 3.631602 .0085921 422.67 0.000 3.614762 3.648442

2021#boy#rural | 3.655141 .0107115 341.24 0.000 3.634146 3.676135

2021#girl#urban | 3.239634 .004042 801.49 0.000 3.231711 3.247556

2021#girl#semi-urban | 3.251179 .008346 389.55 0.000 3.234821 3.267537

2021#girl#rural | 3.271285 .0103312 316.64 0.000 3.251036 3.291534

2023#boy#urban | 3.598072 .0045423 792.13 0.000 3.589169 3.606975

2023#boy#semi-urban | 3.660466 .009078 403.23 0.000 3.642674 3.678259

2023#boy#rural | 3.691901 .0115443 319.80 0.000 3.669275 3.714528

2023#girl#urban | 3.225593 .0042278 762.95 0.000 3.217307 3.23388

2023#girl#semi-urban | 3.237714 .0088845 364.42 0.000 3.220301 3.255127

2023#girl#rural | 3.254432 .0112943 288.15 0.000 3.232295 3.276568

----------------------------------------------------------------------------------------

. margins year#immigrant

Predictive margins Number of obs = 556,424

Model VCE: Robust

Expression: Predicted mean belonging, predict()

-----------------------------------------------------------------------------------------------------------

| Delta-method

| Margin std. err. z P>|z| [95% conf. interval]

------------------------------------------+----------------------------------------------------------------

year#immigrant |

2017#Student and parents born in Finland | 3.705991 .0029592 1252.36 0.000 3.700191 3.71179

2017#One foreign-born parent | 3.651152 .0113156 322.67 0.000 3.628974 3.67333

2017 #|

Born in Finland, foreign-born parents | 3.687757 .0235181 156.81 0.000 3.641663 3.733852

2017#Student and parents born abroad | 3.465676 .0215483 160.83 0.000 3.423442 3.507909

2019#Student and parents born in Finland | 3.493207 .0025908 1348.34 0.000 3.488129 3.498285

2019#One foreign-born parent | 3.432726 .0094397 363.65 0.000 3.414224 3.451227

2019 #|

Born in Finland, foreign-born parents | 3.519348 .0181037 194.40 0.000 3.483866 3.554831

2019#Student and parents born abroad | 3.357595 .0167015 201.04 0.000 3.324861 3.390329

2021#Student and parents born in Finland | 3.419471 .0025893 1320.64 0.000 3.414397 3.424546

2021#One foreign-born parent | 3.361085 .0092533 363.23 0.000 3.342949 3.379221

2021 #|

Born in Finland, foreign-born parents | 3.48083 .0173926 200.13 0.000 3.446741 3.514919

2021#Student and parents born abroad | 3.326749 .0157666 211.00 0.000 3.295847 3.357651

2023#Student and parents born in Finland | 3.414776 .0027745 1230.77 0.000 3.409338 3.420214

2023#One foreign-born parent | 3.366586 .0094544 356.09 0.000 3.348055 3.385116

2023 #|

Born in Finland, foreign-born parents | 3.493115 .0175897 198.59 0.000 3.45864 3.52759

2023#Student and parents born abroad | 3.441814 .0142772 241.07 0.000 3.413831 3.469796

-----------------------------------------------------------------------------------------------------------
