# Supplementary material for: Is social connectedness still in decline after the Covid-19 pandemic? Cohort trends in secondary school students in Finland between 2017 and 2023
Source: BMC Psychol. 2025 Sep 29;13:1071. doi: 10.1186/s40359-025-03394-5 (PMC12482009; doi:10.1186/s40359-025-03394-5)
Supplement: Supplementary file 3 — Supplementary material 3: Data diagnostics and missing data [file 40359_2025_3394_MOESM3_ESM.docx]

Supplementary material 4: Data diagnostics and missing data

Polychoric correlations between number of close friends, feelings of loneliness and sense of belonging at school

. polychoric loneliness closefriend belonging

Polychoric correlation matrix

loneliness closefriend belonging

loneliness 1

closefriend -.42812093 1

belonging -.42516799 .33206563 1

Correlations between the background characteristics in the models

. collin year gender schoollevel degree urbanrural immigrant

(obs=559,461)

Collinearity Diagnostics

SQRT R-

Variable VIF VIF Tolerance Squared

----------------------------------------------------

year 1.01 1.00 0.9910 0.0090

gender 1.00 1.00 0.9984 0.0016

schoollevel 1.02 1.01 0.9805 0.0195

degree 1.03 1.02 0.9694 0.0306

urbanrural 1.04 1.02 0.9617 0.0383

immigrant 1.01 1.00 0.9913 0.0087

----------------------------------------------------

Mean VIF 1.02

Cond

Eigenval Index

---------------------------------

1 5.9842 1.0000

2 0.4311 3.7256

3 0.2232 5.1781

4 0.1966 5.5171

5 0.1200 7.0618

6 0.0449 11.5433

7 0.0000 3221.0230

---------------------------------

Condition Number 3221.0230

Eigenvalues & Cond Index computed from scaled raw sscp (w/ intercept)

Det(correlation matrix) 0.9481

Multicollinearity not evident based on VIF (variance inflation factor) and tolerance values.

Multicollinearity should be inspected when:

- VIF values are large, individual VIF greater than 10 or average VIF greater than 6
- tolerance values are small, close to zero, tolerance less than .1

Source: <http://www.philender.com/courses/categorical/notes2/collin.html>

Proportions missing in outcome by background characteristics

0 = outcome not missing

1 = outcome missing

Number of close friends

. tab year closefriendmi, row chi

| closefriendmi

year | 0 1 | Total

-----------+----------------------+----------

2017 | 132,542 2,360 | 134,902

| 98.25 1.75 | 100.00

-----------+----------------------+----------

2019 | 153,263 1,903 | 155,166

| 98.77 1.23 | 100.00

-----------+----------------------+----------

2021 | 159,117 1,679 | 160,796

| 98.96 1.04 | 100.00

-----------+----------------------+----------

2023 | 148,842 2,382 | 151,224

| 98.42 1.58 | 100.00

-----------+----------------------+----------

Total | 593,764 8,324 | 602,088

| 98.62 1.38 | 100.00

Pearson chi2(3) = 337.0845 Pr = 0.000

. tab gender closefriendmi , row chi

| closefriendmi

gender | 0 1 | Total

-----------+----------------------+----------

boy | 284,642 5,837 | 290,479

| 97.99 2.01 | 100.00

-----------+----------------------+----------

girl | 307,523 2,089 | 309,612

| 99.33 0.67 | 100.00

-----------+----------------------+----------

Total | 592,165 7,926 | 600,091

| 98.68 1.32 | 100.00

Pearson chi2(1) = 2.0e+03 Pr = 0.000

. tab schoollevel closefriendmi , row chi

| closefriendmi

schoollevel | 0 1 | Total

----------------+----------------------+----------

lower secondary | 333,813 5,787 | 339,600

| 98.30 1.70 | 100.00

----------------+----------------------+----------

upper secondary | 170,243 1,147 | 171,390

| 99.33 0.67 | 100.00

----------------+----------------------+----------

vocational | 89,708 1,390 | 91,098

| 98.47 1.53 | 100.00

----------------+----------------------+----------

Total | 593,764 8,324 | 602,088

| 98.62 1.38 | 100.00

Pearson chi2(2) = 910.8171 Pr = 0.000

. tab degree closefriendmi , row chi

| closefriendmi

degree | 0 1 | Total

-----------+----------------------+----------

0 | 249,227 1,374 | 250,601

| 99.45 0.55 | 100.00

-----------+----------------------+----------

1 | 312,950 1,576 | 314,526

| 99.50 0.50 | 100.00

-----------+----------------------+----------

Total | 562,177 2,950 | 565,127

| 99.48 0.52 | 100.00

Pearson chi2(1) = 5.9864 Pr = 0.014

. tab immigrant closefriendmi , row chi

| closefriendmi

immigrant | 0 1 | Total

----------------------+----------------------+----------

Student and parents b | 499,583 2,664 | 502,247

| 99.47 0.53 | 100.00

----------------------+----------------------+----------

One foreign-born pare | 42,021 246 | 42,267

| 99.42 0.58 | 100.00

----------------------+----------------------+----------

Born in Finland, fore | 12,345 127 | 12,472

| 98.98 1.02 | 100.00

----------------------+----------------------+----------

Student and parents b | 20,705 268 | 20,973

| 98.72 1.28 | 100.00

----------------------+----------------------+----------

Total | 574,654 3,305 | 577,959

| 99.43 0.57 | 100.00

Pearson chi2(3) = 242.8107 Pr = 0.000

. tab urbanrural closefriendmi , row chi

| closefriendmi

urbanrural | 0 1 | Total

-----------+----------------------+----------

urban | 422,900 6,279 | 429,179

| 98.54 1.46 | 100.00

-----------+----------------------+----------

semi-urban | 101,112 1,281 | 102,393

| 98.75 1.25 | 100.00

-----------+----------------------+----------

rural | 69,752 764 | 70,516

| 98.92 1.08 | 100.00

-----------+----------------------+----------

Total | 593,764 8,324 | 602,088

| 98.62 1.38 | 100.00

Pearson chi2(2) = 79.6427 Pr = 0.000

Loneliness

. tab year lonelinessmi , row chi

| lonelinessmi

year | 0 1 | Total

-----------+----------------------+----------

2017 | 132,986 1,916 | 134,902

| 98.58 1.42 | 100.00

-----------+----------------------+----------

2019 | 153,638 1,528 | 155,166

| 99.02 0.98 | 100.00

-----------+----------------------+----------

2021 | 159,118 1,678 | 160,796

| 98.96 1.04 | 100.00

-----------+----------------------+----------

2023 | 148,868 2,356 | 151,224

| 98.44 1.56 | 100.00

-----------+----------------------+----------

Total | 594,610 7,478 | 602,088

| 98.76 1.24 | 100.00

Pearson chi2(3) = 293.3735 Pr = 0.000

. tab gender lonelinessmi , row chi

| lonelinessmi

gender | 0 1 | Total

-----------+----------------------+----------

boy | 285,202 5,277 | 290,479

| 98.18 1.82 | 100.00

-----------+----------------------+----------

girl | 307,809 1,803 | 309,612

| 99.42 0.58 | 100.00

-----------+----------------------+----------

Total | 593,011 7,080 | 600,091

| 98.82 1.18 | 100.00

Pearson chi2(1) = 2.0e+03 Pr = 0.000

. tab schoollevel lonelinessmi , row chi

| lonelinessmi

schoollevel | 0 1 | Total

----------------+----------------------+----------

lower secondary | 334,376 5,224 | 339,600

| 98.46 1.54 | 100.00

----------------+----------------------+----------

upper secondary | 170,414 976 | 171,390

| 99.43 0.57 | 100.00

----------------+----------------------+----------

vocational | 89,820 1,278 | 91,098

| 98.60 1.40 | 100.00

----------------+----------------------+----------

Total | 594,610 7,478 | 602,088

| 98.76 1.24 | 100.00

Pearson chi2(2) = 894.2700 Pr = 0.000

. tab degree lonelinessmi , row chi

| lonelinessmi

degree | 0 1 | Total

-----------+----------------------+----------

0 | 249,469 1,132 | 250,601

| 99.55 0.45 | 100.00

-----------+----------------------+----------

1 | 313,342 1,184 | 314,526

| 99.62 0.38 | 100.00

-----------+----------------------+----------

Total | 562,811 2,316 | 565,127

| 99.59 0.41 | 100.00

Pearson chi2(1) = 19.3634 Pr = 0.000

. tab immigrant lonelinessmi , row chi

| lonelinessmi

immigrant | 0 1 | Total

----------------------+----------------------+----------

Student and parents b | 500,258 1,989 | 502,247

| 99.60 0.40 | 100.00

----------------------+----------------------+----------

One foreign-born pare | 42,062 205 | 42,267

| 99.51 0.49 | 100.00

----------------------+----------------------+----------

Born in Finland, fore | 12,374 98 | 12,472

| 99.21 0.79 | 100.00

----------------------+----------------------+----------

Student and parents b | 20,720 253 | 20,973

| 98.79 1.21 | 100.00

----------------------+----------------------+----------

Total | 575,414 2,545 | 577,959

| 99.56 0.44 | 100.00

Pearson chi2(3) = 339.0515 Pr = 0.000

. tab urbanrural lonelinessmi , row chi

| lonelinessmi

urbanrural | 0 1 | Total

-----------+----------------------+----------

urban | 423,480 5,699 | 429,179

| 98.67 1.33 | 100.00

-----------+----------------------+----------

semi-urban | 101,262 1,131 | 102,393

| 98.90 1.10 | 100.00

-----------+----------------------+----------

rural | 69,868 648 | 70,516

| 99.08 0.92 | 100.00

-----------+----------------------+----------

Total | 594,610 7,478 | 602,088

| 98.76 1.24 | 100.00

Pearson chi2(2) = 101.5763 Pr = 0.000

Belonging at school

. tab year belongingmi , row chi

| belongingmi

year | 0 1 | Total

-----------+----------------------+----------

2017 | 127,235 7,667 | 134,902

| 94.32 5.68 | 100.00

-----------+----------------------+----------

2019 | 154,101 1,065 | 155,166

| 99.31 0.69 | 100.00

-----------+----------------------+----------

2021 | 159,859 937 | 160,796

| 99.42 0.58 | 100.00

-----------+----------------------+----------

2023 | 150,651 573 | 151,224

| 99.62 0.38 | 100.00

-----------+----------------------+----------

Total | 591,846 10,242 | 602,088

| 98.30 1.70 | 100.00

Pearson chi2(3) = 1.7e+04 Pr = 0.000

. tab gender belongingmi , row chi

| belongingmi

gender | 0 1 | Total

-----------+----------------------+----------

boy | 283,689 6,790 | 290,479

| 97.66 2.34 | 100.00

-----------+----------------------+----------

girl | 306,613 2,999 | 309,612

| 99.03 0.97 | 100.00

-----------+----------------------+----------

Total | 590,302 9,789 | 600,091

| 98.37 1.63 | 100.00

Pearson chi2(1) = 1.8e+03 Pr = 0.000

. tab schoollevel belongingmi , row chi

| belongingmi

schoollevel | 0 1 | Total

----------------+----------------------+----------

lower secondary | 332,693 6,907 | 339,600

| 97.97 2.03 | 100.00

----------------+----------------------+----------

upper secondary | 170,228 1,162 | 171,390

| 99.32 0.68 | 100.00

----------------+----------------------+----------

vocational | 88,925 2,173 | 91,098

| 97.61 2.39 | 100.00

----------------+----------------------+----------

Total | 591,846 10,242 | 602,088

| 98.30 1.70 | 100.00

Pearson chi2(2) = 1.6e+03 Pr = 0.000

. tab degree belongingmi , row chi

| belongingmi

degree | 0 1 | Total

-----------+----------------------+----------

0 | 248,580 2,021 | 250,601

| 99.19 0.81 | 100.00

-----------+----------------------+----------

1 | 313,017 1,509 | 314,526

| 99.52 0.48 | 100.00

-----------+----------------------+----------

Total | 561,597 3,530 | 565,127

| 99.38 0.62 | 100.00

Pearson chi2(1) = 239.8072 Pr = 0.000

. tab immigrant belongingmi , row chi

| belongingmi

immigrant | 0 1 | Total

----------------------+----------------------+----------

Student and parents b | 499,240 3,007 | 502,247

| 99.40 0.60 | 100.00

----------------------+----------------------+----------

One foreign-born pare | 41,965 302 | 42,267

| 99.29 0.71 | 100.00

----------------------+----------------------+----------

Born in Finland, fore | 12,327 145 | 12,472

| 98.84 1.16 | 100.00

----------------------+----------------------+----------

Student and parents b | 20,545 428 | 20,973

| 97.96 2.04 | 100.00

----------------------+----------------------+----------

Total | 574,077 3,882 | 577,959

| 99.33 0.67 | 100.00

Pearson chi2(3) = 675.4980 Pr = 0.000

. tab urbanrural belongingmi , row chi

| belongingmi

urbanrural | 0 1 | Total

-----------+----------------------+----------

urban | 421,743 7,436 | 429,179

| 98.27 1.73 | 100.00

-----------+----------------------+----------

semi-urban | 100,654 1,739 | 102,393

| 98.30 1.70 | 100.00

-----------+----------------------+----------

rural | 69,449 1,067 | 70,516

| 98.49 1.51 | 100.00

-----------+----------------------+----------

Total | 591,846 10,242 | 602,088

| 98.30 1.70 | 100.00

Pearson chi2(2) = 17.4529 Pr = 0.000
